# Adaptation in Outbred Sexual Yeast is Repeatable, Polygenic and Favors Rare Haplotypes

**DOI:** 10.1093/molbev/msac248

**Published:** 2022-11-11

**Authors:** Robert A Linder, Behzad Zabanavar, Arundhati Majumder, Hannah Chiao-Shyan Hoang, Vanessa Genesaret Delgado, Ryan Tran, Vy Thoai La, Simon William Leemans, Anthony D Long

**Affiliations:** Department of Ecology and Evolutionary Biology, School of Biological Sciences, University of California, Irvine; Department of Ecology and Evolutionary Biology, School of Biological Sciences, University of California, Irvine; Department of Ecology and Evolutionary Biology, School of Biological Sciences, University of California, Irvine; Department of Ecology and Evolutionary Biology, School of Biological Sciences, University of California, Irvine; Department of Ecology and Evolutionary Biology, School of Biological Sciences, University of California, Irvine; Department of Ecology and Evolutionary Biology, School of Biological Sciences, University of California, Irvine; Department of Ecology and Evolutionary Biology, School of Biological Sciences, University of California, Irvine; Department of Biomedical Engineering, School of Engineering, University of California, Irvine; Department of Ecology and Evolutionary Biology, School of Biological Sciences, University of California, Irvine

**Keywords:** yeast, experimental evolution, evolve and resequence, complex traits

## Abstract

We carried out a 200 generation Evolve and Resequence (E&R) experiment initiated from an outbred diploid recombined 18-way synthetic base population. Replicate populations were evolved at large effective population sizes (>10^5^ individuals), exposed to several different chemical challenges over 12 weeks of evolution, and whole-genome resequenced. Weekly forced outcrossing resulted in an average between adjacent-gene per cell division recombination rate of ∼0.0008. Despite attempts to force weekly sex, roughly half of our populations evolved cheaters and appear to be evolving asexually. Focusing on seven chemical stressors and 55 total evolved populations that remained sexual we observed large fitness gains and highly repeatable patterns of genome-wide haplotype change within chemical challenges, with limited levels of repeatability across chemical treatments. Adaptation appears highly polygenic with almost the entire genome showing significant and consistent patterns of haplotype change with little evidence for long-range linkage disequilibrium in a subset of populations for which we sequenced haploid clones. That is, almost the entire genome is under selection or drafting with selected sites. At any given locus adaptation was almost always dominated by one of the 18 founder's alleles, with that allele varying spatially and between treatments, suggesting that selection acts primarily on rare variants private to a founder or haplotype blocks harboring multiple mutations.

## Introduction

A detailed understanding of the genetic mechanisms through which organisms adapt to stressful environments has important implications for a wide range of issues such as climate change, antibiotic resistance, and bioengineering. Studying adaptation in a controlled laboratory environment through experimental evolution has become a mainstay of evolutionary genetics (Garland and Rose 2009). Such studies have furthered our understanding of the dynamics of evolution at both a phenotypic and genotypic level. A powerful approach to studying evolution in a laboratory setting is the evolve and resequence (E&R) paradigm (Turner et al., 2011), in which populations in a controlled setting are sequenced before and during the course of adaptation to a novel environment to better understand the dynamics of adaptation (reviewed in (Long et al. 2015; Schlotterer et al. 2016)).

Advances made in understanding mechanisms of adaptation have come from E&R studies in microbes, flies, and, more recently, worms. Studies in microbes have been instrumental in furthering our understanding of adaptation in a purely asexual context, which has important implications for the evolution of antibiotic resistance (Barlow and Hall 2003; Toprak et al. 2011; Sousa et al. 2012; Chevereau et al. 2015; Lamrabet et al. 2019), cancer tumorigenesis (Sprouffske et al. 2012; Taylor et al. 2013, 2017), and the acquisition of novel phenotypes (Wildenberg and Murray 2014; López-Malo et al. 2015; Wannier et al. 2018). The majority of such studies are initiated from an isogenic haploid asexual population that evolves in a novel environment; thus, adaptation occurs solely via de novo mutations and adaptation is dominated by selective sweeps and clonal interference between beneficial mutations on different genetic backgrounds (Toprak et al. 2011; Lang et al. 2013; Good et al. 2017). Under this asexual isogenic paradigm, forces such as historical contingency (Toprak et al. 2011), genetic parallelism at the level of genes but not (except for rare exceptions, see Toprak et al. (2011)) specific mutations (Toprak et al. 2011; Tenaillon et al. 2012), and diminishing returns epistasis (Toprak et al. 2011; Wang et al. 2016) matter a great deal to the evolutionary process. Similar studies in asexual isogenic diploids have shown that ploidy can influence the dynamics of adaptation, with diploids often adapting more slowly than haploids, likely due to the effects of Haldane's sieve (Zeyl et al. 2003; Sellis et al. 2011, 2016; Gerstein et al. 2014; Fisher et al. 2018; Marad et al. 2018; Johnson et al. 2021). Diploids also appear to accumulate more potentially deleterious mutations (increased mutational load), and continue to adapt longer than haploids, likely due to the effects of mitotic recombination (Forche et al. 2011; Gerstein et al. 2014; Johnson et al. 2021). Despite these differences, general aspects of adaptation are shared with asexual haploids, including clonal interference and the fixation of beneficial mutations.

However, most higher eukaryotes are obligately sexual, and evolution takes place in the presence of standing variation and recombination. As a result, evolution in asexual isogenics may serve as a poor model for the dynamics of evolution in sexual outbreds. In the presence of standing variation and recombination, adaptation appears to proceed more deterministically, with selection acting on alleles already present in the population (Burke et al. 2010, 2014; Hernandez et al. 2011; Phillips et al. 2016), with de novo mutations generally having a minor role in adaptation over short time-scales (Burke et al. 2014). A frequent observation is that sexual populations maintain high levels of heterozygosity (Burke et al. 2010; Phillips et al. 2016; Barghi et al. 2019; O’Connor et al. 2021), even after hundreds of generations of evolution in a constant environment. Further, studies in budding yeast have shown that adaptation with even modest amounts of recombination is capable of making evolution more efficient, both by decoupling deleterious hitch-hiking mutations from beneficial mutations and by combining multiple beneficial mutations onto a single genetic background (Zeyl and Bell 1997; Gray and Goddard 2012; McDonald et al. 2016). An emerging pattern from Drosophila E&R experiments is that adaptation appears to be fairly polygenic with much of the genome responding to selection (Burke et al. 2010; Phillips et al. 2016; Barghi et al. 2019; Barghi and Schlötterer 2020).

The premier system for E&R in sexual outbred is Drosophila, yet despite progress fly experiments are limited in terms of the number of replicates examined, the number of generations over which an experiment is carried out, and the effective population size that can be efficiently handled (*N_e_* is typically ≪1000). Base populations are most often initiated from hundreds of uncharacterized founders, so the tracking of haplotypes in an evolved population is challenging. Finally, although flies are obligate sexuals, recombination only occurs in females and the number of recombination events per female gamete per generation is ∼5. As a result of constraints imposed by the system, we might expect evolution to be dominated by alleles with large selection coefficients (*s*≫*1/N_e_*) and rather large-sized haplotype blocks, especially if rare alleles are being favored. Arguments have been made that observed patterns of allele frequency change support polygenic adaptation (Barghi et al. 2020), but it is difficult to disentangle polygenic adaptation from “traffic”, the phenomena of several long strongly selected haplotypes trying to simultaneously increase in frequency. It is of interest to confirm that patterns of adaptation observed in sexual populations with *N_e_*≪1,000 match those seen in populations with *N_e_* approaching those of natural populations.

Budding yeast is a model system ideally suited to help resolve these fundamental questions, due to their facultatively sexual life cycle and the ease of maintaining very large populations with a high degree of replication for many hundreds of generations. Previous work has been instrumental in describing the dynamics of adaptation in outcrossing populations of budding yeast (Burke et al. 2014; McDonald et al. 2016; Kosheleva and Desai 2018; Leu et al. 2020). We extend previous E&R experiments that used an outcrossing population of budding yeast derived from four highly diverged founder strains (the SGRP-4X) carried through twelve rounds of meiosis followed by random mating to break up linkage blocks (Parts et al. 2011; Cubillos et al. 2013; Burke et al. 2014) by employing a base population derived from 18 different founders (Linder et al. 2020). This population harbors considerably more standing variation than previously studied budding yeast populations and allows us to examine patterns of haplotype change over the 18 different starting alleles. Unlike previous work in outbred sexual yeast, we evolve this base population with replication in the presence of different chemical challenges, modeling how short-term evolution occurs when an organism encounters a novel environment. Weekly outcrossing shuffles evolving haplotypes throughout the course of evolution, allowing us to better distinguish between polygenic adaptation and linked hitch-hiking. This experimental design has several advantages: a large effective population size is maintained (>10^5^), the experiment has a high degree of replication, a wide-range of cellular processes are perturbed, a highly outcrossed base population is employed, and founder haplotypes are known and can be tracked. These features of our model system allow for general inferences about the nature of adaptation in outbred sexual populations.

## Results

**Fig. 1.**
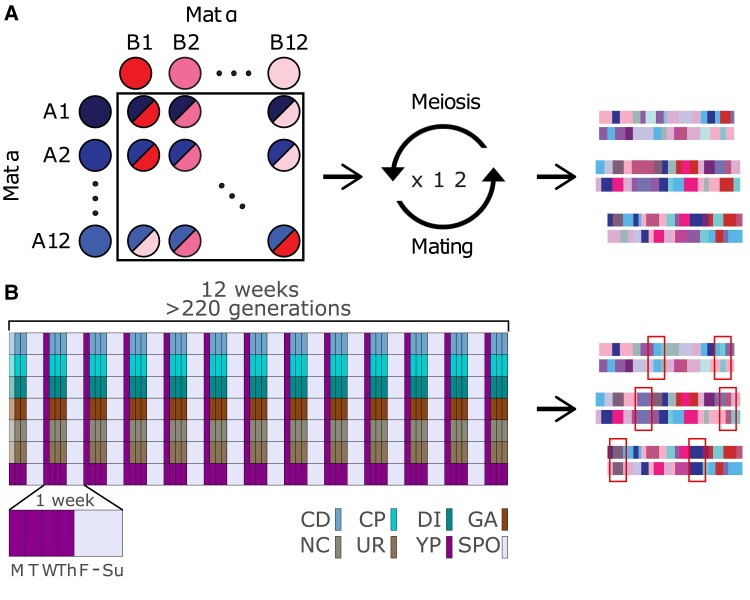
Schematic of the long-term evolution experiment. Panel (*A*) depicts the creation of the base population used in this study. In total, 11 Mat a and 11 Mat @ strains were crossed in a full diallel, followed by 12 rounds of forced outcrossing to create a highly diverse and mosaic population of diploids. Panel (*B*) depicts the long-term evolution experiment. Details of the regimen are in the methods. The first day of the experiment, a different, normally lower dose of the chemical stressor was used to acclimate cells to the chemical.

*Experimental evolution in budding yeast:* Yeast were evolved under a set of conditions chosen to mimic a ∼200 generation burst of adaptation to a novel environment in a recombining diploid outbred population maintaining a large population size. A highly outbred diploid population was used to initiate a 16-fold replicated evolve-and-resequence (E&R) experiment that explored the response to 16 different chemical challenges (supplementary table S1, Supplementary Material online) over 12 weeks. A schematic of the weekly evolution regimen is depicted in figure 1.

Each week populations experienced daily serial passages Tuesday through Friday in media supplemented with the different chemicals. Every Friday populations were sporulated, spores were recovered on Mondays, mated, and diploids recovered on Tuesday mornings. Initial concentrations of drugs were chosen so that near the beginning of the experiment cultures grew roughly 10-fold per 24 h (i.e., a slightly higher drug concentration results in the population not being able to survive daily 10-fold dilutions). We thus estimate near the start of the experiment that the populations were going through roughly 10–12 cell divisions per week in the presence of the chemical challenges (supplementary fig. S1, Supplementary Material online), and another 8 during the Friday through Monday drug-free culturing, and a single meiotic generation. Since budding yeast experience ∼90 cross-overs per meiosis (Mortimer et al. 1991) and there is one meiosis per week the average initial recombination rate per adjacent-gene per cell division is estimated to be roughly 90/(6000*20) = 0.0008. As the yeast adapt and the number of cell divisions per week increase that rate drops (by perhaps as much as 3–4 fold). This initial recombination rate is ∼4-folder higher than the per gene recombination rate experienced in obligately sexual *Drosophila melanogaster* during experimental evolution. Each evolved population was initiated from >1M cells, the serial transfer bottlenecks were estimated to contain ∼10–20M cells, we recovered ∼3M haploid spores per population and ∼750,000 diploids after mating each Tuesday (supplementary fig. S1, Supplementary Material online), although this number undoubtedly vary between replicates and over each week. Despite a huge census population size throughout much of the week, the effective population size of the experiment is likely close to 750,000. This population size is orders of magnitude larger than typical outbred sexual E&R experiments and is even larger than the effective population size of the human species by some estimates (Henn et al. 2012).

To examine the genomic response to adaptation, we carried out short-read Illumina pooled sequencing of each population, with the base population sequenced to 2226X. Although we initially sequenced 221 populations, for most of the analyses carried out in this paper, we focus on 55 populations from 7 chemical treatments that remained “sexual”, their selection is detailed below. These 55 evolved populations were sequenced to an average depth of 111X with a range 38X–867X (Methods and supplementary fig. S2, Supplementary Material online). Since the gDNA used to create the libraries was obtained from ∼10^8^ cells, coverage at the 182,460 queried SNPs estimates the frequency of that SNP in the population with a binomial error almost entirely associated with sequencing coverage, as opposed to sequencing coverage plus error due to a finite pool of individuals (Burke et al. 2010). We used those observed SNP frequencies and a sliding window approach to estimate the 18 founder haplotype frequencies at 11,604 loci spaced every 1 kb throughout the genome for the base population and each evolved population. There is little advantage in estimating haplotype frequencies at a resolution greater than one estimate per 1 kb, as the mean absolute haplotype frequency change between adjacent positions in founder AB3 is close to zero (estimated at ∼0.009, supplementary fig. S3, Supplementary Material online; also c.f. figs. 4 and 9). Our previous work further suggests that the average absolute error in the per position individual haplotype frequency estimates is ∼0.01 at the sequencing coverages we employ. That is, haplotype frequency estimates are associated with much lower errors than would be predicted based solely on coverage and binomial sampling, consistent with other studies (Long et al. 2011; Kessner et al. 2013; Burke et al. 2014; Tilk et al. 2019; Linder et al. 2020). To validate that the haplotype caller remains accurate over many generations of evolution, we sequenced three week 12 populations to an average genome wide depth of 843x and compared the inferred haplotype frequencies to the frequencies of SNPs private to individual founders as was done in (Linder et al. 2020). Supplementary fig. S4, Supplementary Material online shows that the haplotype caller is perhaps even more accurate after multiple rounds of recombination have occurred, validating the utility of our caller over a burst of evolutionary time.

**Fig. 2.**
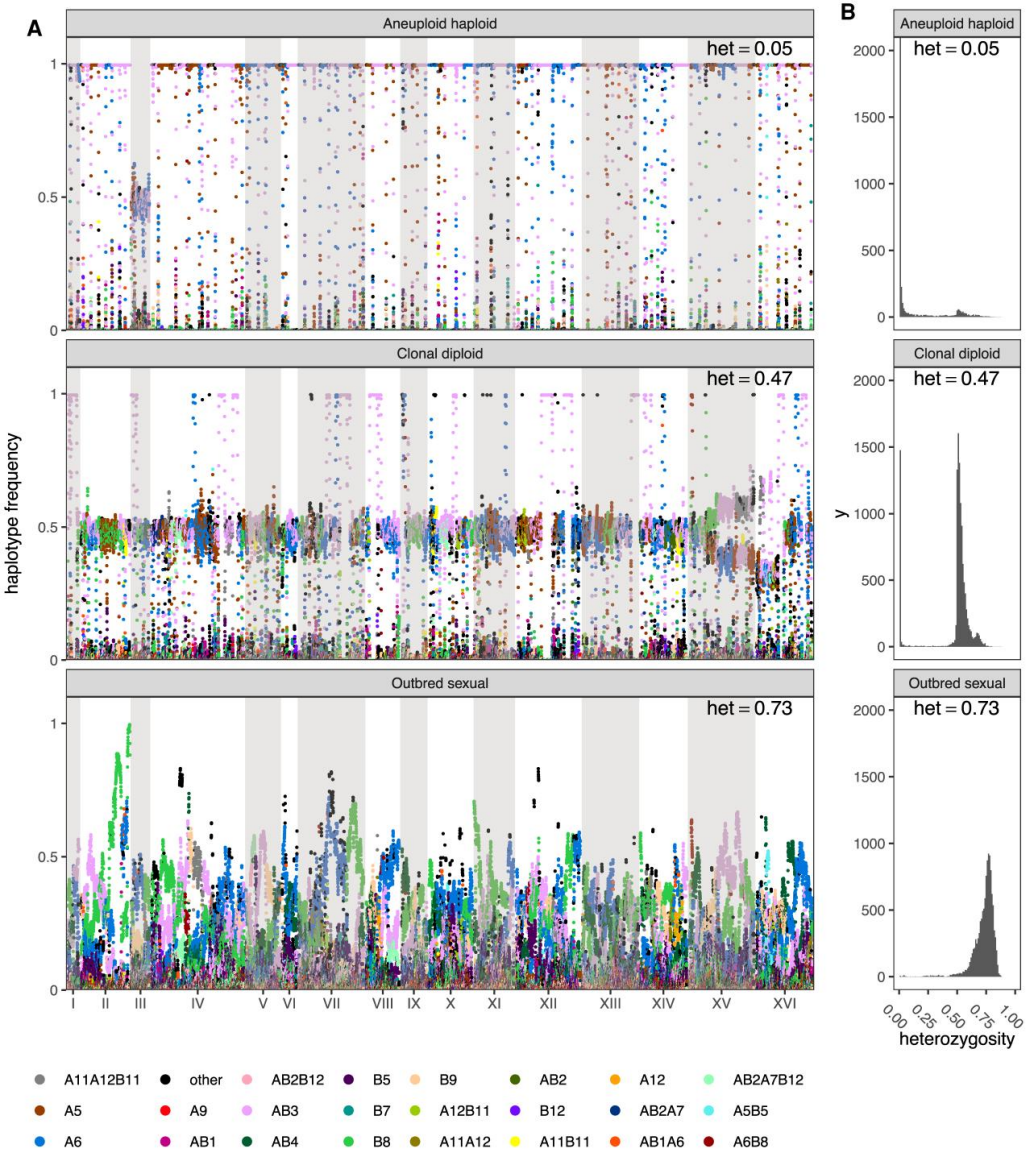
Different classes of evolved populations. In (*A*), raw haplotype frequency plots are shown of (from top to bottom) a caffeine replicate, a glacial acetic acid replicate, and a cadmium chloride replicate classified as aneuploid haploid, heterozygous clonal, and outbred sexual, respectively. In (*B*) are shown histograms of the per-site heterozygosity of the corresponding replicates. The average per-site heterozygosity is shown at the top of each panel.

Since the populations were evolved under conditions that initially forced outcrossing once every ∼20 cell divisions, at the molecular level we expected the population to maintain high levels of diversity and only display localized changes in allele frequencies and losses of heterozygosity associated with adaptation, a prediction of evolutionary models in outbred sexuals (Long et al. 2015). Despite forced outcrossing, asexual single clone “cheaters” came to dominate some populations; recognizable by reduced levels of heterozygosity and founder haplotype frequencies close to 100% (for doubled haploid clones) or 50% (for diploid clones). Manhattan plots of per population heterozygosity and haplotype frequency change confirm the emergence of cheaters for many replicates and drugs (fig. 2). It appears that a combination of selection, forced meiosis, and aggressive spore recovery using chemical and mechanical shearing can favor the emergence of genotypes that can avoid our targeted sexual life-cycle. Despite the emergence of cheaters being an interesting observation, to understand evolution in sexual outbred populations, we identified a subset of chemicals and populations in which cheaters were unable to gain a foothold and sweep to high frequency using a classification metric (see Material and Methods) that allowed us to classify populations into one of three non-overlapping types: aneuploid haploids, clonal diploids, or outbred sexuals (e.g., see fig. 2). We observed 106 drug/population combinations out of 221 (∼48%) that appeared to remain sexual throughout our experiment (supplementary table S2, Supplementary Material online). Different chemical challenges are associated with different rates of cheater emergence. Lower 12-week average OD630 (a measure of growth rate) is associated with lower week 12 heterozygosity measures across different chemical treatments (supplementary fig. S5*A*, Supplementary Material online), a pattern that similarly manifests as a smaller fraction of populations classified as outbred sexuals as a function of decreasing growth rate (supplementary fig. S5*B*, Supplementary Material online). This observation is consistent with the idea that the subset of chemical treatments that present a greater initial selective challenge, as measured by slower growth and lower cell densities, are more prone to invasion by a cheater. We hypothesize that given very strong selective pressure (or an absence of standing variation) mutations/aneuploidies of large effect can be favored irrespective of deleterious side-effects. A corollary is that weaker selective pressure favors adaptation via standing variation.

**Fig. 3.**
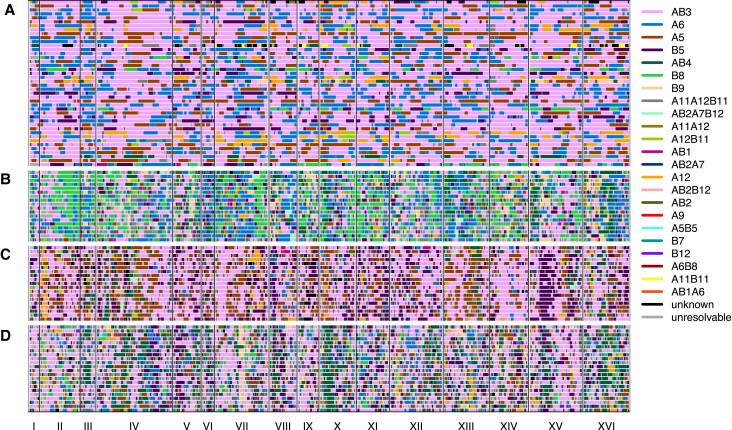
Haploid clones were isolated from the base population (*A*), a week 11 cadmium chloride population (*B*), a week 11 diamide population (*C*) as well as a week 11 sodium chloride population (*D*).

To enable statistical analyses of patterns of evolutionary change in the subset of populations that remained sexual, we additionally required that each chemical treatment retain at least 6 replicate sexual populations (excluding the control YPD treatment) and that genome-wide haplotype frequency estimates for populations within a chemical treatment clustered together compared to different treatments in a distance-based cladogram (supplementary fig. S6, Supplementary Material online). This clustering filter, which removed 23 populations, was an attempt to control for possible cross-contamination events (that cannot be completely eliminated due to the complex weekly transfer regimen). After this aggressive filtering seven chemicals and 55 total replicates (∼25% of our initial starting populations) remained. We focus the remainder of the manuscript on these populations (supplementary fig. S6, Supplementary Material online and table 1). To further show that these populations remained sexual throughout the course of the experiment, we sequenced 59 haploid clones from week 11 across three chemicals, including cadmium chloride, diamide, and sodium chloride as well as 42 haploid clones from the base population (fig. 3). Sequenced week 11 clones show that unrecombined founder haplotype blocks are a median length of 25 kb vs. 87 kb amongst clones derived from the base. The modest number of sequenced clones do not suggest a single high-frequency haplotype dominating the population, an observation consistent with figure 2.

**Table 1.** Chemicals and Replicates Used in This Study.

Chemical Short	Chemical Long	Concentration	# Replicates	Fitness Increase (fold change)^a^
CD	Cadmium chloride	600μM	7	5.0
CP	Chlorpromazine	10μM	8	5.5
DI	Diamide	2.7mM	10	6.7
GA	Glacial acetic acid	120mM	9	3.4
NC	NaCl	950mM	10	3.8
UR	Urea	650mM	6	4.1
YP	YPD	NA	5	0.9

a Fitness increases are calculated as the estimated growth rate at week 12 over the growth rate at week 0.

*Fitness increased substantially after 12 weeks of evolution:* Like previous experimental evolution studies, we observe considerable increases in fitness over the 12 weeks of the experiment, with the exception of the YPD-only condition, which slightly lost fitness at week 12 (table 1), potentially due to a fitness trade-off between mitotic growth and mating efficiency, a phenomenon that has been documented previously ((Lang et al. 2009); also see (Zeyl and Bell 1997; Li et al. 2019) in which there was no observable fitness gain in mitotic growth in the YPD-only condition). That said, it is difficult to estimate cell numbers in these populations due to a tendency of some culture to flocculate, and as a result our fitness estimates are only approximations. Over the six chemical stressors, we observe a week 12 increase in fitness ranging from ∼3-fold to slightly more than 6-fold (see methods for details on measuring growth rate). These fitness increases, if measured without error, imply the number of asexual cell divisions per week by the end of the experiment has increased from roughly 10 to 30–60. The estimated fitness gains compare favorably to an experiment in diploid budding yeast carried out for a similar number of generations with recombination, in which fitness was found to increase by ∼1.5-fold (Kosheleva and Desai 2018) in YPD-only media in a 2-way outcrossed population. Other studies where 2- and 4-way populations of outbred budding yeast were evolved asexually for 50 generations in two different chemicals (rapamycin and hydroxyurea) found fitness gains of ∼1.4-fold and ∼1.5-fold, respectively (Li et al. 2019). Studies in haploid budding yeast have shown smaller overall fitness gains after >100 generations of purely asexual evolution from an isogenic base population (Lang et al. 2011; Kryazhimskiy et al. 2014; Levy et al. 2015; Fisher et al. 2018). Further, after 50,000 generations of purely asexual evolution, (Tenaillon et al. 2016) found an average fitness increase of ∼1.7-fold amongst all replicate *Escherichia coli* populations during adaptation to nutrient limitation. These comparisons suggest that the presence of recombination and high levels of standing variation led to much larger fitness gains than are normally seen in experiments with less variation and/or no recombination, at least over short bursts of evolution in a novel environment, consistent with early studies that have more directly made this observation (Johnson et al., 2021). Although any such inference is necessarily weak, as the experiments described vary in many respects beside the presence of sex and standing variation (e.g., chemostat vs. flask, chemical vs. YPD, initial chemical concentrations, *etc*.).

*De novo mutations and aneuploidies:* Across the seven chemicals used, we detect several different aneuploidies (supplementary table S3, Supplementary Material online). Of note, the entirety of Chromosome II was duplicated in all seven cadmium chloride replicates- the well-known cadmium transporter, *PCA1*, is present on this chromosome, which is a suggestive explanation for this particular aneuploidy. Additionally, a single sodium chloride replicate has the entirety of Chromosomes III and V duplicated, while four urea replicates have an additional copy of Chromosome V. Partial aneuploidies were also detected, including the end of Chromosome XVI in half of the chlorpromazine replicates and one at the beginning of Chromosome XVI in 6 of 9 glacial acetic acid replicates, as well as a duplication of part of Chromosome VI that occurred in all 10 diamide replicates. This last partial duplication had 2-fold increased relative coverage in evolved populations as compared to the base in 9 of 10 replicates, implying that an additional two copies of this region are present in all individuals. Of note, a single partial duplication of the end of Chromosome XVI was detected in all conditions, with the exception of cadmium chloride and urea. Several founders that increased in frequency at the end of Chromosome XVI in the aneuploid populations have naturally occurring duplications that fall within this region, some of which include the spore-specific water channel, *AQY1*, a potential candidate driving this particular aneuploidy. Only two partial aneuploidies were detected in YPD, suggesting that the other aneuploidies detected in the six chemical treatments are likely specifically beneficial for the chemical(s) they are detected in. Despite the existence of several presumably adaptive aneuploidies, it is unlikely these aneuploidies account for the bulk of adaptation as they tend to involve a small number of chromosomes or replicates, and we see strong genome-wide responses to selection.

We also detected 219 single-nucleotide variants (SNVs) that reached frequencies of 20% or higher in the populations they were detected in that likely spontaneously arose during the course of evolution (supplementary table S4, Supplementary Material online; see Methods for how de novo mutations were detected). An average of 31 de novo SNVs were detected per condition, from 14 in cadmium chloride to 56 in sodium chloride. These mutations reached an average frequency of 28% over the 12 weeks of evolution, with 20 total SNVs at a frequency at or greater than 50% and 3 total SNVs reaching a frequency of 75% or greater. The presence of multi-hit genes within a single condition is strongly suggestive of mutations that affect the chemicals they are detected in. In total, we found only one gene which was hit in two different chlorpromazine replicates (*DSF2*, a gene of unknown function). As with aneuploidies, although a subset of the de novo mutations detected are likely adaptive, they are unlikely to explain a large fraction of adaptive change since they generally only occur in a subset of genes and/or replicates. Furthermore, if de novo mutations dominated the selective response we would not expect to see strong convergence of haplotype frequencies over replicates as described below. Nonetheless, in our large effective population size E&R experiment, newly arising mutations seem to play a larger role in adaptation compared to past experiments with much smaller values of *N*_*e*_*μ*.

**Fig. 4.**
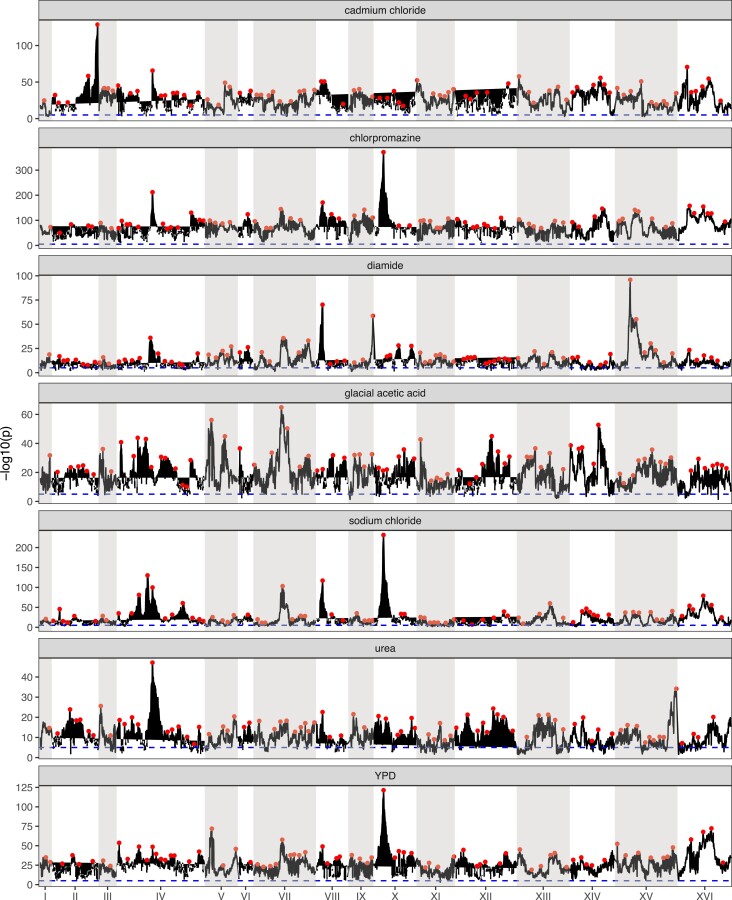
Genome-wide Manhattan plots of the LOD score for all chemicals. Red dots represent putative local peaks. The dashed blue horizontal line marks an LOD score of 5.

*A genome-wide scan for haplotype frequency change suggests highly polygenic adaptation:* A genome-wide scan for genomic regions contributing to adaptation was carried out for each of the seven drug treatments to detect regions showing consistent haplotype change relative to the base population. Figure 4, Manhattan plots of genome-wide −log10(*P*) values for all chemical treatments, shows that across all treatments the vast majority of the genome is above the significance cutoff. Across all treatments, an average of 95.7% of the genome was above the significance threshold (supplementary table S5, Supplementary Material online). A simple linear model regressing the number of replicates per treatment onto the genome-wide average −log10(*P*) value shows that there is no correlation between the number of replicate populations and the average genome-wide significance (supplementary fig. S8*A*, Supplementary Material online), suggesting that our test statistic is not strongly impacted by the number of replicates. Similarly, a regression of the average −log10(*P*) value on the number of local haplotypes (since not all 18 founder haplotypes are represented at all positioning in the genome) contributing to a test (supplementary fig. S8*B*, Supplementary Material online) suggests that our test statistic is not strongly impacted by the number of haplotypes contributing to a test. Overall patterns of haplotype frequency change suggest that adaptation is highly polygenic, with nearly all regions of the genome being either directly under selection or in linkage disequilibrium (LD) with selected regions.

**Fig. 5.**
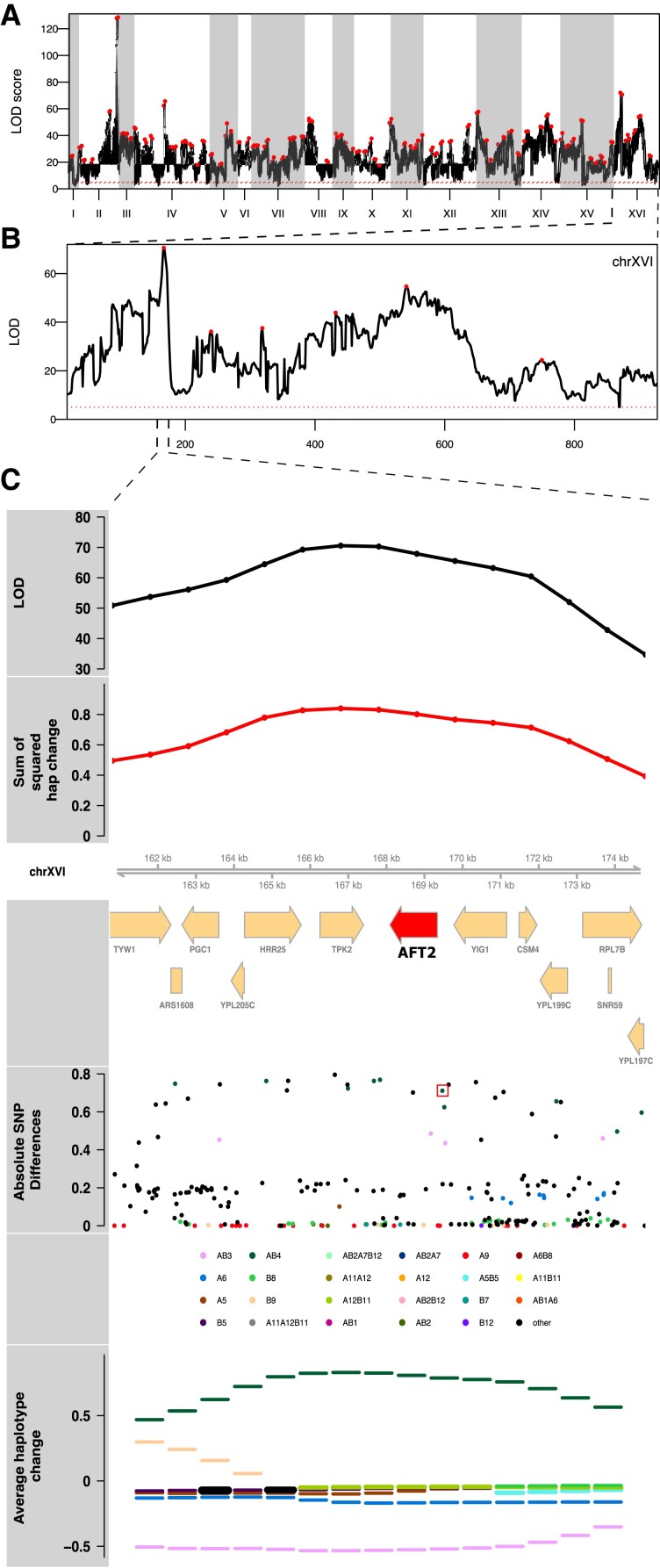
The results of a genome-wide scan for potentially causal genomic regions for cadmium chloride treatment is shown (panel *A*). Panel (*B*) depicts a close-up of chromosome XVI, where one of the highest peaks was detected and which emphasizes the polygenic nature of the adaptive response. Panel (*C*) zooms in under the peak on Chromosome XVI, where there is a candidate causative SNP (red box) just upstream of the candidate gene *AFT2* in the most changed haplotype, AB4, that creates a Yap1*p* binding site (see main text).

*Identifying candidate major effect genes*: Despite much of the genome exhibiting haplotype change, there are clearly sub-regions of the genome not inconsistent with major genes (or gene regions) playing a role in adaptation. Due to the highly recombined and diverse nature of the base population, as well as the number of replicates and population sizes maintained throughout evolution, we are often able to resolve potentially major effect loci to a small number of genes. In the Supplementary Results, Supplementary Material online, we describe candidate genes and/or potentially causative variants under the top three peaks from each condition that suggest targets for future functional validation. In several cases, there are strong a priori or *posteriori* candidate genes contained within the detected interval. Unlike a typical E&R experiment in sexual outbreds, we can track changes in frequency at founder haplotypes and observe that adaptation is often due to the action of a single haplotype (below). As a result, candidate variants unique to that haplotype are strong candidate causative variants. Further, by virtue of the founders being de novo sequenced through both long-read and short-read technology (Linder et al. 2020), we are able to identify both candidate SNPs as well as candidate structural variants private to a particular founder. Figure 5 exemplifies a peak with a single haplotype increasing in frequency identified in cadmium chloride treatment. Panel A depicts the genome-wide LOD score, while panel B zooms in on Chromosome XVI, and panel C shows the LOD scores and the sum of the squared haplotype change for a peak on this chromosome, as well as the genes under the peak, the absolute SNP differences, and the average haplotype change over all cadmium chloride replicates. A red box in panel C highlights a candidate causative SNP among ∼25 private to the most increased haplotype (MIH) that creates a binding site for Yap1p, a transcription factor known to be involved in cadmium chloride tolerance (Wemmie et al. 1994). Supplementary figs. S8 and S9, Supplementary Material online show examples of peaks underlying chlorpromazine, diamide, sodium chloride, and urea tolerance.

**Fig. 6.**
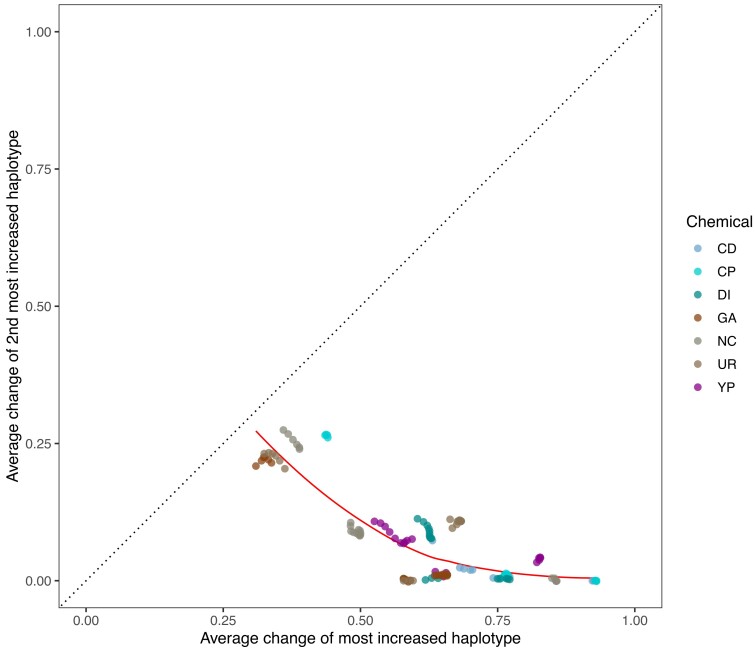
Average change of the most increased haplotype vs. that of the next most increased haplotype at each of the 21 major peaks detected.

*Adaptation appears to be most often due to the action of a single haplotype:* By virtue of our quantifying the dynamics of 18 founder haplotypes, we can identify the founder haplotype(s) showing the most evolutionary change over replicate evolved populations and correlate variation unique to that haplotype (or haplotypes) with the change. Figure 6, which compares the average change in frequency of the most increased haplotype (MIH) vs. the next most increased haplotype per site across the 21 peaks discussed above, reveals that, across all chemical treatments, the most increased haplotype shows much larger gains than the next most increased haplotype. On average, the MIH increases by about 63%, while the next MIH increases by about 7.3%, a more than 8-fold difference. Furthermore, this disparity is consistent when comparing the MIH against the next MIH at each site across the genome, with the MIH increasing by an average of ∼23.8% and the next MIH increasing by an average of ∼9.5%, a ∼2.5 fold difference (supplementary fig. S11*A*, Supplementary Material online). To show that this effect is not solely due to the initial frequency of the founder haplotypes in the population, supplementary fig. S11*B*, Supplementary Material online shows the average change of the MIH vs. the next MIH genome-wide only for sites at which the next MIH starts out at a higher initial frequency than the MIH. The average increase of the MIH at these sites is ∼23%, while the average increase of the next MIH is ∼11%, a slightly more than 2-fold difference. These results show that adaptation across the conditions tested is dominated by a single haplotype increasing in frequency, suggesting that adaptation is often either due to rare variants private to a single founder or due to a block of several mutations defining a local haplotype. There is little evidence that intermediate frequency SNPs, present on multiple haplotypes, individually drive adaptation, as we rarely observe two haplotypes increasing in a coincident fashion. For intermediate frequency SNPs to be important in adaptation in this system, at a minimum, the idea of considerable traffic between closely linked factors needs to be invoked.

**Fig. 7.**
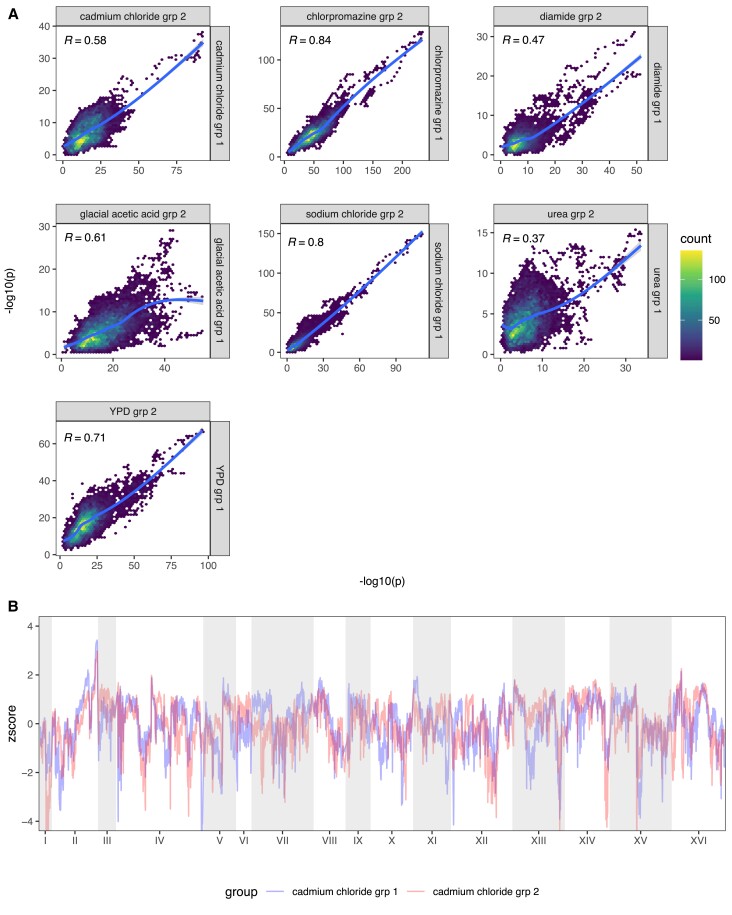
Repeatability of evolution amongst replicate populations. Panel (*A*) depicts the Spearman correlation between the LOD scores of randomly grouped replicates from each chemical treatment. Panel (*B*) shows the genome-wide z-score of each cadmium chloride group superimposed on one another.

**Fig. 8.**
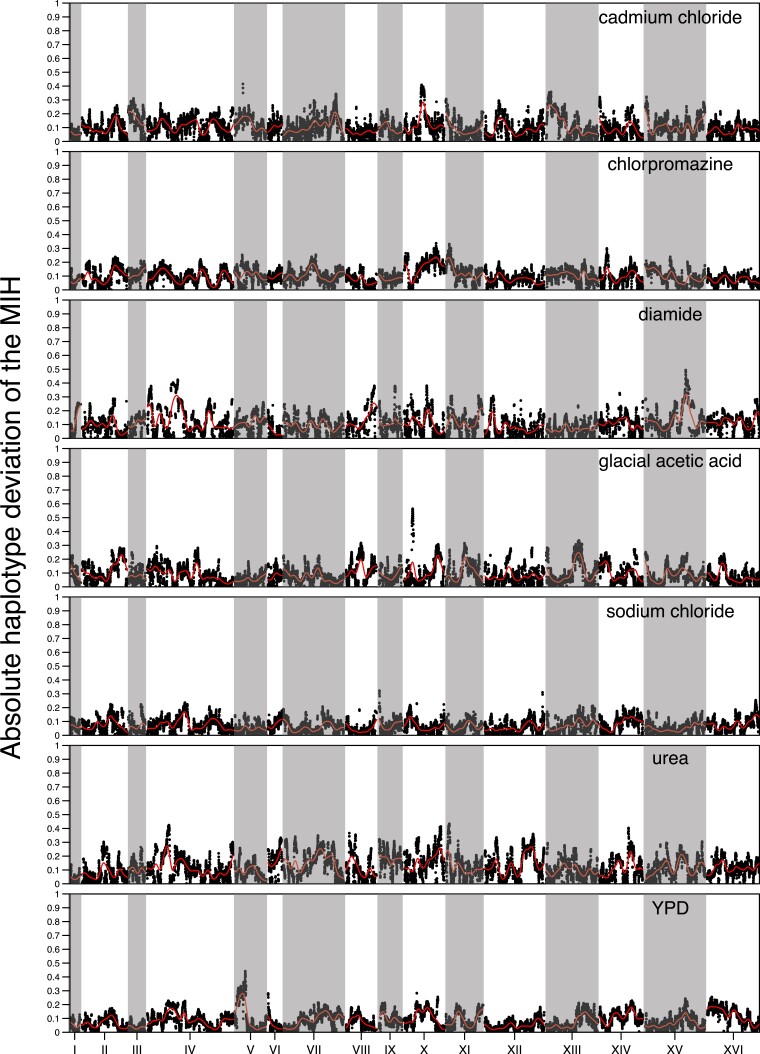
The absolute deviation genome-wide of the MIH frequency of a single replicate from the average of the remaining replicates for each chemical. The red line represents a kernel regression run using the ksmooth() function in R with kernel set at “normal” and bandwidth set at 100,000.

*The repeatability of evolution in a large-sized, outcrossed, sexual population*: We determined the counts of minor alleles for all SNPs private to a single founder in our high sequence coverage base population (supplementary fig. S12, Supplementary Material online). About 23% of private SNPs are not observed in the base population despite it being sequenced to over 2000X coverage, suggesting they were either lost during the 12 rounds of intercrossing or are present at an extremely low frequency (<1/2000). The average MAF of private SNPs is ∼4.4%, with ∼79% of all MAFs greater than 0.4% and 100% of all MAFs at a frequency of greater than 0.04%. Given these minor allele frequencies and *N_e_* = 750,000 in our evolution experiment, theory predicts that almost all variants should be visible to selection and evolution should be essentially deterministic (given a primarily additive genetic architecture). To verify this, we calculated Spearman's rho between haplotype frequencies for all pairs of replicates within a treatment at each locus in the genome. Supplementary fig. S13, Supplementary Material online depicts the average such value for all pairs of populations within treatment and shows that the extent to which adaptive strategies are correlated is dependent on the specific chemical stressor employed. Figure 7*A* looks at the correlation in −log10(*P*) values between two equal sized groups of replicates within chemicals across all loci and shows that regions of the genome showing more dramatic changes often share those changes across evolutionary replicates, with more subtle changes being less consistent. Chlorpromazine, sodium chloride, glacial acetic acid, YPD, and cadmium chloride all exhibit highly correlated genomic responses, while diamide and urea exhibit moderately correlated responses. To examine spatial patterns in correlated responses we transformed the −log10(*P*) values to z-scores (to somewhat mute the impact of major genes) for two groups of three different cadmium chloride replicates that typify responses and display those scores as a Manhattan plot (fig. 7*B*). The figure suggests that evolution is extremely replicable over much of the genome, with only a small number of regions showing noticeable differences in adaptive outcomes. A final metric that seems to indicate that the within treatment replicates are behaving similarly is to examine the absolute deviation of the first replicate of each chemical from the mean haplotype frequency of the remaining replicates at each position across the genome for the MIH (fig. 8). The absolute deviations are similar in magnitude across all chemicals, for the most part falling between 0 and 0.2, again suggesting that changes in haplotype frequency across replicates are similar across most of the genome; although a handful of local peaks can indicate regions of the genome where the responses were less coordinated. This point is further illustrated by supplementary fig. S14, Supplementary Material online, which looks at haplotype frequency changes amongst cadmium chloride replicates at a single chromosome for the MIH at each position and shows that the same founder haplotype is generally increasing in the majority of replicate populations.

We measure within drug repeatability as the average absolute deviation between the frequency of the MIH for each replicate and the mean of the remaining replicates (100% repeatability would take on a value of 0 under this metric). We observed that average heterozygosity at 12 weeks is positively correlated with this measure of repeatability (supplementary fig. S15, Supplementary Material online). That is, evolution appears most deterministic in populations that maintain higher amounts of genetic variation and presumably experience softer selection over the course of evolution. supplementary fig. S16, Supplementary Material online shows that although populations maintain high levels of heterozygosity overall, heterozygosity does become depleted at regions that experience the largest amount of change across the different chemical treatments.

*The deterministic trajectory of evolution in large, outcrossed populations*: Due to the large population sizes and relatively large amount of recombination experienced by our populations, we are in a unique position to examine how deterministic allele frequency changes are under the different selection regimens imposed. Supplementary fig. S17*A*, Supplementary Material online shows that, for the 21 leading factor peaks examined, there is no correlation between the initial frequency of the MIH and the average change in haplotype frequency, while supplementary fig. S17*B*, Supplementary Material online shows that there is a moderate relationship between the initial haplotype frequency and the average change in the next MIH. Similarly, when looking at the entire genome (supplementary fig. S18*A*, Supplementary Material online and B), there appears to be no relationship between the initial haplotype frequency and average haplotype change in both the MIH and the next MIH. This suggests that the selective pressure is high enough that evolution is highly deterministic across a wide range of initial frequencies in the base population.

**Fig. 9.**
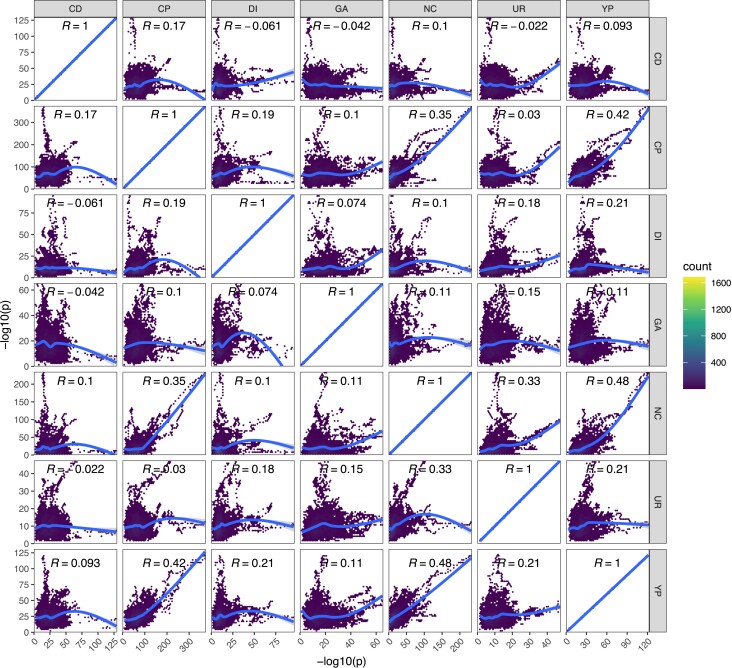
Detecting pleiotropy. Shown are the Spearman correlations of the genome-wide LOD scores for each pair of chemical treatments.

*We observe some instances of strong pleiotropy, despite it being rare overall:* By virtue of evolving our population to several different stressors with replication with all populations initiated at the same time from the same base population, we can test for pleiotropy by identifying regions of the genome showing similar responses between treatments relative to pure within treatment replicates. Although an examination of the average −log10(*P*) values by chemical treatment (fig. 4) suggests the existence of several regions responding to multiple stressors, and hence exhibiting pleiotropy (discussed below), pleiotropy is not a general feature of the experiment. Figure 9 shows the correlation between average −log10(*P*) values for each pairwise combination of drugs.

**Fig. 10.**
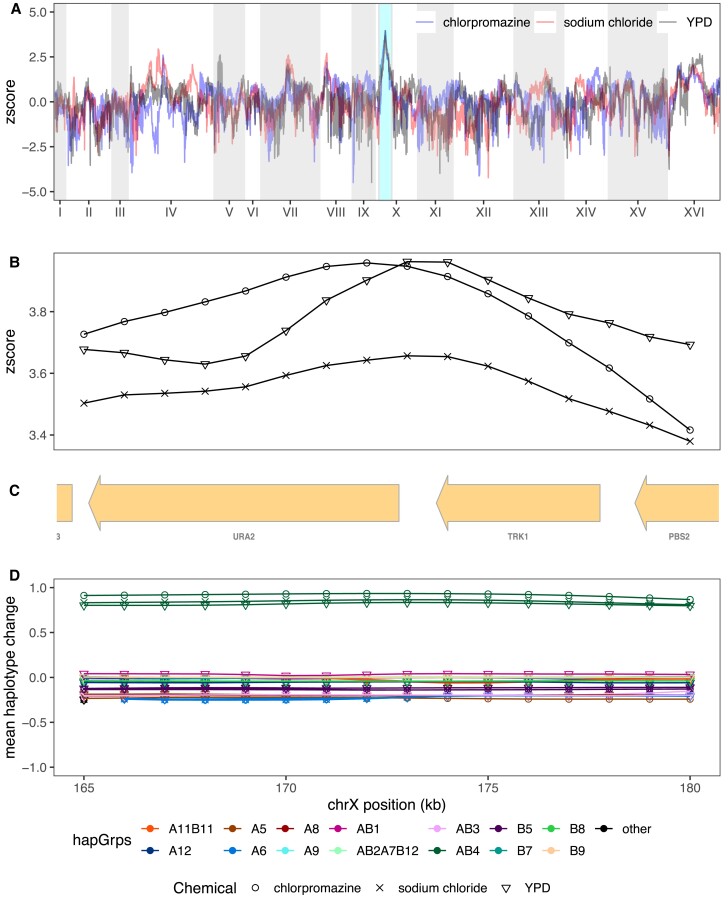
The most striking example of pleiotropy was detected on chromosome X. Panel (*A*) depicts the superimposed genome-wide z-scores of chlorpromazine, sodium chloride, and YPD, with the shared peak on Chromosome X highlighted in teal. Panel (*B*) zooms in on z-scores at the shared peak, with panel (*C*) showing the genes present in this region and panel (*D*) depicting the mean haplotype change amongst the three different chemicals.

Although pure replicates are highly correlated in their evolutionary response (c.f. fig. 7*A*) this is not the case for between treatment comparisons in general, with most loci showing a response specific to the stressor they were exposed to. Several chemicals appear to elicit moderately correlated responses with one another, including YPD, sodium chloride, chlorpromazine, and urea, whereas cadmium chloride, diamide, and glacial acetic acid all exhibited more unique genome-wide patterns of selection. As detailed above, the parameters of this experiment enable us to identify relatively narrow intervals containing potentially pleiotropic gene regions, which are discussed in detail in the Supplementary Results, Supplementary Material online. One striking example of pleiotropy occurs on Chromosome X for a subset of three chemicals (chlorpromazine, sodium chloride, and YPD). All exhibit very similar evolutionary dynamics (fig. 10*A*and*B*) and a single founder (AB4) shows a large increase in frequency over the course of the experiment (fig. 10*D*). The peak change occurs at the three genes *TRK1, URA2,* and *PBS2* (fig. 10*C*), all three harbor SNPs private to AB4, and all are potential functional candidates based on annotation. It is notable that our base population is a *URA3* null (Linder et al. 2020), and perhaps *URA2* compensates for some subtle lost function in non-minimal media.

## Discussion

*Yeast as a model for evolution in outbred sexual species*: Evolve and Resequence (E&R) experiments have emerged as a paradigm for understanding evolutionary change. Under this paradigm, replicate populations are lab evolved and next-generation sequenced, often as a pooled sample, to test evolutionary models. E&R experiments have been carried out in a variety of systems, including asexual microbes or more rarely microbes with occasional bouts of sex initiated from a single isogenic individual, or facultative or obligate sexual populations evolving with sex starting from a variable base population (referred to as “sexual outbreds”). Highly replicated large *N_e_* experiments can be carried out in microbes lacking sex and/or variation (Tenaillon et al. 2012), but evolution is then due entirely to newly arising mutations and clonal interference dominates evolutionary dynamics (Lang et al. 2013). It is not clear asexual systems effectively model evolution in outbred sexuals, and previous work has shown that adaptation can occur more efficiently in the presence of standing variation and recombination (Zeyl and Bell 1997; Gray and Goddard 2012; McDonald et al. 2016). Sexual outbreds are of special interest to evolutionary biology, since virtually all multicellular eukaryotes fall into this category. Evolution in sexual outbreds has been most effectively modeled using *Drosophila melanogaster*, since flies are naturally outbred and are obligate sexuals. But the logistics of maintaining fly populations dictate fly E&R experiments have a modest number of replicate populations, tens as opposed to hundreds of generations of evolution, and effective population sizes (*N_e_*) generally less than one thousand individuals. In E&R experiments selected sites (or local haplotypes) must have selection coefficients larger than ∼1/*N_e_* for selection to efficiently act on them. The result is that regions responding to selection in Drosophila E&R experiments are likely associated with selection coefficients of between 0.1% and 1%. To what extent would larger population sizes affect experimental outcomes?

In a previous paper, we manipulated yeast to create an outbred diploid highly recombined base population (Linder et al. 2020), and here we evolve that population under a set of conditions that mimic a burst of adaptation to a novel environment in an outbred sexual population. We evolved populations at a large effective population size (*N_e_* estimated to be 750,000) for 216 generations under a regime where sex is forced roughly once every 18 generations. Such a scheme results in an initial per gene per generation recombination rate ∼4X that achieved in *Drosophila*. The large effective population size approximates that of some natural populations, allows for intermediate frequency alleles with selection coefficients approaching 0.0001% to be visible to natural selection, and the high rate of recombination and smaller potential selective coefficients, in theory, should reduce the hitch-hiking footprint length associated with alleles responding to selection. We finally adapted the yeast to several different chemical challenges in parallel. Despite some limitations of the system (discussed below), it is a powerful model for understanding evolution in large populations of diploid outbred eukaryotes.

*Limitations to using budding yeast as a model of obligate sexual evolution:* Unlike obligate sexual species (such as *D. melanogaster* and humans), *S. cerevisiae* is a facultatively sexual organism, capable of reproducing both asexually and sexually. In nature, budding yeast are diploid and predominantly divide asexually, with meiosis induced under harsh conditions, most likely to increase chances of survival (Ruderfer et al. 2006; Tsai et al. 2008). We manipulated yeast to create a large outbred diploid population which we forced to adapt to several environmental stressors, with adaptation occurring at large population sizes with frequent recombination. This artificial situation was designed to mimic adaptation in outbred sexuals, but clearly is not natural for yeast. Despite stringent selection for sporulation followed by random mating, ∼50% of our populations experienced the emergence and dominance of what appear to be asexual “cheaters’ (i.e., individuals capable of making it through the evolution regimen without sporulating/mating). The propensity of cheaters to emerge and dominate a population correlated with the intensity of the selective pressure, with populations experiencing more intense selection being more likely to evolve cheaters. One potential explanation is that mutations of large effect are favored under these conditions, and the background in which such mutations occur can very quickly reach a high frequency in a population. Another potential explanation is that the small number of asexual cells divisions during permissive recovery of diploids are relatively more favorable to cheaters when growth in the presence of a drug is extremely slow. Furthermore, the forced outcrossing procedure itself imparts a large fitness cost on the adapting populations, as evidenced by the nearly 600-fold drop in viable cell numbers from the beginning of sporulation to just after random mating. This substantial fitness cost associated with sporulation, spore isolation and dispersal, and mating results in strong selective pressure for individuals to evolve strategies that “game the system”. One potential means by which this could take place is through the construction and maintenance of fortified cell walls capable of withstanding the chemical and mechanical forces spores face during isolation and dispersal. It may even be possible that vegetative cells evolve that have the 4-layered cell walls of haploid spores without going through the process of meiosis. However, confirming the exact mechanism(s) of how asexual “cheaters” survive is beyond the scope of this paper. Despite the emergence of cheaters, after aggressive filtering we were able to identify 55 replicate populations across seven chemical conditions that appear to have remained sexual throughout the experiment. These populations can serve as a model of evolution in sexual outbreds.

*Comparison to other long-term evolution experiments in facultatively sexual microbes:* Some of the earliest experiments to document the advantages of recombination with standing variation used the facultatively sexual algae *Chlamydomonas reinhardtii* (da Silva and Bell 1996; Colegrave et al. 2002). These ground-breaking studies found that even a small amount of recombination can lead to long-term fitness gains compared to populations maintained purely asexually. However, only a few (up to 3) rounds of recombination were carried out and the molecular mechanisms behind the adaptive gains remain unknown. With the advent of next-generation sequencing technologies, the ability to resequence the genomes of evolved populations emerged as a powerful means to uncover the genetic changes underlying adaptation. Budding yeast have become a powerful model system to interrogate the genetic underpinnings of adaptation in the presence of sex and/or standing variation. Initial studies used highly outcrossed populations (that had gone through 12 rounds of recombination) generated from two or four founder strains (Parts et al. 2011; Cubillos et al. 2013, 2017; Vázquez-García et al. 2017; Li et al. 2019). Very large, replicated diploid populations were exposed to high temperature, hydroxyurea, or rapamycin and were propagated solely asexually for between 25 and 54 generations before whole-genome resequencing of evolved and ancestral populations. These studies were pivotal in dissecting the role of standing variation on initial adaptation in outbred diploids but were carried out for a small number of generations without recombination during the selective regimen. In contrast, two studies evolved large, replicate populations of budding yeast (on the order of ∼10^5^ cells, with between 3 and 4 replicates sequenced per condition) with regular rounds of recombination interspersed between periods of mitotic growth (McDonald et al. 2016; Kosheleva and Desai 2018; Leu et al. 2020). These populations evolved for many more generations than previous work (≥ 1,000 generations) with 11 or 24 interspersed rounds of recombination and showed that recombination alters the dynamics of adaptation. This being said, McDonald et al. (2016) and Leu et al. (2020) initiated their experiment from an isogenic base population with all adaptation due to newly arising mutations, so standing variation was absent. At present, only two studies in budding yeast we are aware of have evolved populations with recombination in the presence of standing variation. One of these varied the frequency of recombination with replicate diploid populations of ∼10^5^ cells for nearly 1,000 generations (Kosheleva and Desai 2018), but evolution was only initiated from two genetically diverged founder strains. The other study took a 4-way population through 540 generations of mitotic evolution with 18 rounds of recombination (Burke et al. 2014) with populations maintained at ∼10^6^ cells. Both studies propagated budding yeast in rich media under a lab domestication regimen. Our study builds off these experiments by evolving populations with large effective population sizes with several replicates per condition for 216 mitotic generations interspersed with 12 rounds of recombination. Unlike previous studies in budding yeast, this study was initiated with much higher levels of standing variation, thus enabling us to better model long-term evolution in an outbred sexual species. And indeed, the higher levels of initial standing variation were associated with large fitness gains. Further, by evolving replicate populations in parallel to many different environments, we can make general inferences about the characteristics of adaptation. Finally, by virtue of the base population of this experiment being initiated from 18 different trackable founder alleles we can characterize the starting frequencies of variants important in adaptation.

*Comparison to other long-term evolution experiments in obligate sexuals:* Most E&R studies to date have utilized *Drosophila spp.* to model evolution in sexual outbred diploids, with *Caenorhabditis elegans* and/or *remanei* perhaps emerging as an alternate model (Chelo and Teotónio 2013; O’Connor et al. 2021). A large advantage of these studies is the high levels of genetic diversity segregating in the evolving populations. Drosophila E&R studies are often initiated from >100 founders, in stark contrast to sexual yeast populations initiated from four or fewer founders. Here, we employ 18 founders, still far fewer than fly experiments, but much more variation than previous budding yeast E&R experiments. The bulk of previous E&R work in *Drosophila* has been carried out for fewer than 100 generations (Orozco-terWengel et al. 2012; Remolina et al. 2012; Turner and Miller 2012; Huang et al. 2014; Jalvingh et al. 2014; Martins et al. 2014; Tobler et al. 2014; Franssen et al. 2015; Jha et al. 2015; Barghi et al. 2017; Griffin et al. 2017; Michalak et al. 2017; Hardy et al. 2018; Barghi et al. 2019; Kelly and Hughes 2019; Sørensen et al. 2020; Shahrestani et al. 2021), with a handful of studies carried out for between 100 to 200 generations (Turner et al. 2011; Zhou et al. 2011; Hoedjes et al. 2019; Iranmehr et al. 2021; Kawecki et al. 2021) and only a few studies maintained for between 400 to greater than 900 generations (Burke et al. 2010; Phillips et al. 2016, 2018). Minimum census population sizes in these studies have ranged from hundreds to greater than 2,000 individuals (Shahrestani et al. 2021), with this work maintaining effective population sizes likely one to two orders of magnitude greater. The majority of studies in flies have evolved 2–5 replicate populations under 2–3 selection regimens, with the exception of a recent study in *D. simulans* which used 10 replicates (Barghi et al. 2019) as well as a recent study in *D melanogaster* which used 6 replicates (Kawecki et al. 2021). The physical size of haplotype blocks changing in concert in fly E&R experiments varies among experiments (but is difficult to estimate as individuals are rarely sequenced), but a few studies have suggested that such blocks can extend over several megabases (Franssen et al. 2015; Barghi et al. 2019), a significant fraction of the 120 Mb Drosophila genome. The smaller size of haplotype blocks in our base population, with a median block size in the base population estimated at 66 kb (Linder et al. 2020), along with higher rates of recombination per gene per generation, and an ability to track founder haplotypes allow the yeast system to detect adaptive genomic regions at a resolution much higher than in flies.

*Aneuploidies and* de novo *mutations are part of the adaptive response:* Previous studies in budding yeast using populations with high levels of standing variation report conflicting results about the role of de novo mutations in adaptation, with two studies (both carried out with recombination) determining that mutations play a negligible role (Burke et al. 2014; Kosheleva and Desai 2018), while another study showed that the role of mutations can depend strongly on the type of stressor used (Li et al. 2019). Of the 219 total de novo SNP/small INDEL mutations detected here only 20 reached a frequency ≥50% and only three reached a frequency ≥75%. This suggests that de novo mutations had a limited role in the adaptive response, which is further supported by the lack of multi-hit genes within a chemical treatment (only one gene was hit twice in a single condition). Aneuploidies have been shown to have an important role in the adaptive response to a plethora of stressors, especially during the first few hundred generations of adaptation (reviewed in Gilchrist and Stelkens (2019)). We detect seven total whole-chromosome duplications (across five different chromosomes) as well as 41 (30 unique) total segmental duplications. Of the whole-chromosome duplications, five were present in only a single replicate in each of the chemicals they were detected in, while one was present in four replicates and another in all seven cadmium chloride replicates. Of the 41 total segmental duplications detected, 29 were detected only in a single replicate. These patterns suggest that aneuploidies can play an important role in adaptation, even in populations undergoing recombination. This is likely because some isolates of budding yeast are known to tolerate aneuploidies well (Gilchrist and Stelkens 2019; Hose et al. 2020). Further, the presence of aneuploidies does not seem to have affected our ability to map potentially causal loci, implying that recombination is still active in these regions. Aneuploidies do, however, seem to have a limited role in the adaptive response as all chromosomes showed extensive change in haplotype frequencies, and some replicates do not seem to harbor aneuploidies, yet have evolved.

*Long-term adaptation is highly polygenic:* A large number of studies in flies claim that adaptation in outbred sexuals is highly polygenic, with many loci throughout the genome responding to selection (Clark et al. 2007; Coop et al. 2009; Burke et al. 2010; Turner et al. 2011; Orozco-terWengel et al. 2012; Remolina et al. 2012; Turner and Miller 2012; Chandler 2014; Huang et al. 2014; Tobler et al. 2014; Jha et al. 2015; Kang et al. 2016; Phillips et al. 2016; Barghi et al. 2017; Griffin et al. 2017; Hardy et al. 2018; Barghi et al. 2019; Hoedjes et al. 2019; Sørensen et al. 2020; Kawecki et al. 2021; Shahrestani et al. 2021; Tusso et al. 2021), but to some extent the inference of polygenicity is based on a visual inspection of Manhattan plots. *Very few studies attempt to estimate the minimum number of factors consistent with observed results*. Although hundreds of factors are consistent with observations, so are as few as 20–30 (Kelly and Hughes 2019). Part of the problem is that E&R experiments start from limited numbers of haplotypes and selection coefficients are potentially >1%, so physically large haplotypes (≫1 Mb) can hitch-hike with a selected site (Nuzhdin and Turner 2013; Franssen et al. 2015; Barghi et al. 2019). The only other studies that evolved yeast with recombination that started from highly recombined populations are (Burke et al. 2014; Kosheleva and Desai 2018) and both identified many regions of potentially small-effect throughout the genome, although major factors were also observed in (Burke et al. 2014). In this study, we find that almost the entire genome appears to respond to each of the seven different selective conditions, implying that most of the genome is either directly under selection or in linkage with regions under selection.

The size of unrecombined haplotype blocks centered on a selected site over a short evolutionary burst is proportional to the selection coefficient divided by the recombination rate per basepair per cell division (Kaplan et al. 1989). For a fixed selection coefficient, we thus predict the physical length of unrecombined hitch-hiking blocks is ∼20X smaller in yeast than flies. Furthermore, since the effective population size is perhaps two orders of magnitude larger in yeast than flies, many additional factors with much smaller selection coefficients should be visible to selection, and those sites would be associated with smaller unrecombined blocks still (perhaps 200–2000X smaller). The large population size, high recombination rate, and observation of the entire genome responding to selection requires a much larger minimum number of factors to explain the apparent polygenic response in yeast relative to flies. This being said, more explicit simulations would be of value as the demography of the yeast evolution experiment involves phases of asexual growth at massive population sizes punctuated by highly sexual episodes and there is some evidence that recombination occurs at hotspots in yeast (Illingworth et al. 2013). Either of these forces could result in larger than expected blocks and fewer factors required to explain the results.

*But also includes genes of large effect:* Although there is a clear genome-wide polygenic response to selection, not all regions of the genome are responding equally, suggesting an important role for genes of large effect. We identify several such regions, many of which are associated with excellent candidate genes, and in many cases patterns of haplotype variation suggest candidate causative polymorphisms. Major factors challenge the dichotomy of the genetic architecture of adaptation being highly polygenic versus more oligo-genetic. Perhaps a more useful metric is to ask: how much of the observed change in fitness is due to the largest five factors (or what is the minimum number of factors required to explain half the fitness change)? This was the initial justification for QTL mapping; although thousands of segregating genes may impact a complex trait, it was believed that identifying the leading few was both possible and had practical value (Thoday and Thompson 1976; Lander and Botstein 1989). Unfortunately, we cannot estimate the relative contribution of these leading factors to total observed fitness gain. It is possible that high-throughput competition experiments employing thousands of barcoded gene replacements could be used to answer this question (Roy et al. 2018; Sadhu et al. 2018), although these experiments remain challenging if the factors being selected harbor multiple potentially interacting causative sites (see below) or if they ignore private alleles. Observed patterns of evolutionary change starting from relatively simple synthetic populations over short evolutionary bursts display complex enough dynamics that they are not trivial to model.

*Selection seems to act predominantly on a single beneficial haplotype:* E&R experiments initiated from an outbred base population typically measure changes in the frequency of SNPs observed following adaptation (Burke et al. 2010; Turner et al. 2011; Zhou et al. 2011; Orozco-terWengel et al. 2012; Remolina et al. 2012; Turner and Miller 2012; Huang et al. 2014; Jalvingh et al. 2014; Martins et al. 2014; Tobler et al. 2014; Franssen et al. 2015; Jha et al. 2015; Phillips et al. 2016, 2018; Barghi et al. 2017, 2019; Griffin et al. 2017; Michalak et al. 2017; Hardy et al. 2018; Hoedjes et al. 2019; Kelly and Hughes 2019; Sørensen et al. 2020; Iranmehr et al. 2021; Kawecki et al. 2021; Shahrestani et al. 2021). Such studies have several strengths, but it is difficult to distinguish SNPs that are drivers versus responders. Other E&R experiments have been initiated from a small number of founder alleles (Cubillos et al. 2013; Burke et al. 2014; Chandler 2014; Li et al. 2019; Tusso et al. 2021), in these cases all selection is always acting on common variants. In a 4-way cross from which asexual selection was carried out and founder haplotype frequencies tracked there was some evidence that the majority of adaptation was due to one founder versus three (Cubillos et al. 2013). This was a curious observation as it suggested selection was, for the most part, not acting on common variants. In this study, initiated from 18 founders, whose frequencies were tracked, the selective response is almost always associated with a single founder haplotype that is favored over all the others. It is clear from Manhattan plots showing the change in frequency of the most increased haplotype that the particular haplotype dominating change varies throughout genome for any given drug, and similarly differs between drugs for any given region. This suggests that selection is almost never acting on evolutionary older intermediate frequency SNPs shared among several founders (or we would expect to see multiple founders increasing in frequency at similar rates). Instead, results suggest that selection is almost always acting on a multi-causative mutation haplotype or a rare allele private to a founder. Our observation that almost all adaptation is associated with single haplotypes is difficult to reconcile with the idea that evolution acts primarily on intermediate frequency causative alleles.

*Long-term evolution appears highly repeatable:* We observed high levels of repeatability across evolutionary replicates within drugs, with responses over drugs being different from one another. The two previous studies in budding yeast starting from an outbred base appeared highly repeatable (Burke et al. 2014; Kosheleva and Desai 2018) as have several studies from flies (Burke et al. 2010; Zhou et al. 2011; Remolina et al. 2012; Turner and Miller 2012; Jalvingh et al. 2014; Martins et al. 2014; Tobler et al. 2014; Jha et al. 2015; Kang et al. 2016; Phillips et al. 2016, 2018; Michalak et al. 2017; Hoedjes et al. 2019; Kelly and Hughes 2019; Iranmehr et al. 2021; Kawecki et al. 2021). Contradictory results have occasionally been reported in flies, in which the level of repeatability is much lower across replicate populations (Griffin et al. 2017; Hardy et al. 2018; Barghi et al. 2019). A classic result in population genetics is that once an allele reaches a frequency of greater than 2/*N_e_s* its dynamics are largely deterministic. As a result, causative alleles at frequencies in our base population greater than 3%, 0.3%, and 0.03% with selective coefficients as small as 0.01%, 0.1%, and 1% are visible to natural selection and should behave somewhat deterministically. Since the majority of SNPs have frequencies in the yeast base population greater than 0.4% (and virtually all SNPs have frequencies greater than 0.04%) we expect evolution to be highly repeatable. In contrast, fly E&R experiments are often initiated from ∼200–500 unknown founder haplotypes and effective population sizes are modest at ≪1,000 individuals. As a result, only alleles present in multiple founder backgrounds have frequencies greater than 2/*N_e_s* and can act deterministically. A corollary is that rare variants present in fly E&R base populations are not visible to selection unless they have large selective coefficients or they drift or draft to a higher allele frequency. The result is that it is difficult for adaptation to be due to rare alleles in Drosophila experiments, and experiments may appear unrepeatable if adaptation is often due to alleles with frequencies close to 2/*N_e_s*. It is unclear if repeatability is a prediction of a highly polygenic model; individual selection coefficients under such a model are ≪ 0.1%, selection cannot distinguish individual causative sites, and selection is instead acting on haplotype blocks that happen to contain tens to hundreds of causative sites whose net effect is visible. The outcome is that precise predictions of the polygenic model with respect to the repeatability of adaptation perhaps depend on the details of the distribution of selection coefficients and whether causative sites are clustered in the genome.

*Pleiotropy appears rare*: In total, seven regions were detected as potentially pleiotropic, with four of these regions showing the same Most Increased Haplotype (MIH) across treatments. As only regions with −log10(*P*) values at least 1.96 standard deviations away from the mean were considered, all potentially pleiotropic sites likely have a large effect on fitness in the environments they were detected in. Theory predicts that such large-effect mutations should only affect a small number of phenotypes, as most such effects should be deleterious (Fisher 1930; Orr 2000), which would, at first glance, appear to run counter to what we see. In agreement with theory, previous work has shown that mutations that increase fitness in the environment they were detected in (the “home” environment) can have similar effects on fitness in subtly different environments, but can have drastically different effects in different environments (Kinsler et al. 2020). This occurs because, although mutations do affect several phenotypes, only a small number of phenotypes are relevant to the condition mutations evolve in. However, in rare instances, mutations could have similarly beneficial effects in very different environments as well. Another study in budding yeast has shown that although evolution tends to lead to specialization in the home environment, leading to smaller fitness gains or losses when individuals from that population are exposed to a very different environment, exceptions can occur (Jerison et al. 2020). Together, these results point to the conclusion that, while rare, alleles can have large beneficial effects in multiple different environments. This is consistent with what we observe, as only a handful of potentially large-effect sites have similarly large effects in different environments. Alternatively, overlapping aspects of the different environments used in this study may cause a phenotype important to adaptation in one environment to be important in other environments as well, leading to the same variant having similar effects across environments.

*The difficulty of validating candidate variants*: Validation of potentially causative candidate genes is beyond the scope of the current study. Gene knock-outs would not necessarily additionally implicate proposed candidate genes (since genes are often candidates due to their known loss of function phenotype), so more difficult allele replacement experiments are required. That evolution is primarily due to single rare haplotypes suggests that in many cases the candidate “allele” is not a single mutational event. Furthermore, showing that a single base change confers a selective advantage does not show that this change explains the observed haplotype change. To demonstrate this, a single base change would need to be competed against alleles in which a larger region is substituted. Despite the high levels of recombination we attempt to maintain, we were unable to determine the length of haplotypes that are responding to selection. Validation via saturated replacement of all SNPs, singly and in combination, present on selected haplotype blocks is becoming possible using emerging high-throughput CRISPR/Cas9 methods (Akhmetov et al. 2018; Roy et al. 2018; Sadhu et al. 2018), although if selected haplotypes are associated with dozens of mutations (Pritchard 2001; Thornton et al. 2013) these approaches may fail to explain haplotypic change. High-throughput methods that compete entire regions in otherwise standard backgrounds hold some promise for future work.

## Conclusion

We manipulated yeast to serve as a model system for understanding evolution in outbred sexual diploid species harboring high levels of standing variation (i.e., virtually all higher eukaryotes). We evolved replicate yeast populations in the presence of several chemical stressors for 216 generations with forced sex approximately once every 18 generations, at effective populations sizes approaching those of some natural populations. Like previous studies in Drosophila, we observe major genes important in selective response, with much of the genome responding to selection. Patterns of genetic change appear more repeatable than fly E&R experiments, are highly repeatable within drug treatments, but show little convergence across drugs. We estimate the initial rate of recombination per generation per individual per basepair (gene) to be approximately 20X (4X) higher in our yeast compared to fly E&R experiments. This greatly expanded recombinational map makes it increasingly difficult to explain genome-wide responses to selection as being due to a modest number of causative factors and traffic due to linkage disequilibrium. We make the novel observation that genome-wide haplotype change is almost always and replicably driven by only one of the 18 founder haplotypes, suggesting that selection is acting primarily on alleles private to single founders or multi-mutation haplotypes, and rarely intermediate frequency SNPs present on multiple founder haplotypes. The observation that selection almost always acts on a single haplotype likely rules out some polygenic models for the architecture of adaptation, although current models need to be more explicit and capable of making precise predictions.

## Materials and Methods

*Strains and media:* The base population used in this study is a multi-parent population (MPP) derived from a full diallel cross of 18 highly characterized natural haploid founding strains (Cubillos et al. 2009) intercrossed for 12 generations to break up haplotype blocks. This population, 18F12v2, and the 18 founding strains are described in detail elsewhere (Linder et al. 2020). But briefly, we cross 11 Mata and 11Matα strains in a full diallel, of which four strains (AB1-AB4) entered the cross as both mating types and are the founders of the SGRP-4X (Parts et al. 2011). Details of propagation are in the Supplement.

*Long-term evolution of diploid budding yeast:* The schematic of the long-term evolution regimen is shown in figure 1. Details of media and handling are in the Supplement, although in general concentrations of drugs used throughout the experiment were chosen so that in week one the populations could only just grow 10-fold over 24 h. On the first Tuesday of the entire experiment only, a lower dose of most chemicals was used to acclimate cells to the chemical challenge (to allow cells to physiologically adapt). For the remainder of the experiment, an increased dose was used to maintain a high degree of selective pressure. On Tuesdays, Wednesdays, and Thursdays, 10x serial dilutions of cells were transferred to newly thawed chemical plates using a custom-built liquid-handling robot. Every Thursday, the OD630 of all cultures were read and glycerol stocks were created to maintain a fossil record of evolution. On Fridays, sporulate in deep 24-well plates. On Mondays, sporulation efficiency was checked, cells spheroplasted, and mechanical disruption was further carried out to break open the ascii. The resulting spores were mated and incubated overnight to select for diploids, after which the entire process was repeated for a new week. In total, evolution was carried out for 12 weeks, with an estimated 18 mitotic and 1 meiotic generation occurring each week for a total of 216 mitotic and 12 meiotic generations.

*Whole-genome sequencing of the evolved populations:* Archived week 12 evolved populations were thawed and incubated in deep 96-well plates in fresh YPDamp O/N at 30°C at 175 RPM. Cells were then harvested, DNA prepared, and Nextera Flex reactions carried out (with modifications; cf. Linder et al. 2020). Libraries were sequenced on the HiSeq4000 with PE100 reads. Coverage per sample ranged from 5x to 867x, with a mean coverage of 67x (supplementary fig. S2, Supplementary Material online).

*Isolation of haploid clones:* Three archived week 11 evolved populations (cadmium chloride R04, diamide R01, and sodium chloride R02) as well as the base population were inoculated directly into 2 mL of YPDamp, cells were sporulated, and ascii disrupted to obtain haploid spores. Spores were further disrupted via sonication to separate sister spores and immediately plated onto YPD + cloNAT plates to select for Mat*a* haploids. Haploid clones were grown, pinned onto omnitrays supplemented with mating-type testers as described in Linder et al. (2020). Confirmed Mat *a* haploids were grown, DNA prepared, and additional Nextera libraries made and sequenced.

*Estimation of daily bottlenecks during a week of the long-term evolution regimen:* To estimate bottlenecks and the number of divisions experienced by our populations during a typical week of evolution, a single sexual (see below) YPD population was sampled daily, starting Monday and ending Friday during week 11 of evolution (YPD replicate R10—see supplementary fig. S1*A/B*, Supplementary Material online). Sampling consisted of making serial dilutions, plating, and counting colonies. The maximal amount of bottle-necking experienced by the populations over the course of the week is observed in counts of diploid cells immediately after mating, as this time-point integrates over several previous steps in which spores are killed (due to breaking the ascus, vigorous dispersion, and/or vegetative cell killing steps) and/or are unable to mate or result in inviable diploids.

*Tracking cultures through time via weekly OD630 measurements:* To keep track of which populations went extinct over the course of the experiment, OD630 measurements were taken every Thursday. As a side-effect of measuring the total number of cells through a typical week of evolution (described above), we are able to estimate that cultures likely had not reached saturation at this point, as saturated cultures of *S. cerevisiae* normally reach concentrations of over 5 × 10^8^ cells/mL (Chan et al. 2013).

*Estimation of fitness gains after 12 weeks of evolution:* To estimate the fitness gains of week 12 populations, a single evolved week 12 replicate population from each chemical was recovered in YPD and reacclimated to their respective chemicals of interest. During incubation plates were removed at several time-points over the 24 h period, including: 2.5 h, 8 h, 9.5 h, 12 h, 14 h, 16 h, and 24 h, to estimate the growth rate of each of the week 12 populations and the ancestral population in YPD (OD readings could not be used due to cell clumping), cells separated via sonication, and serial dilutions plated to estimate cell densities. Due to the tendency of these populations to cell clump, it is difficult to estimate cell counts for these populations via traditional methods. To estimate fitness in the base population, we assumed cultures grown in the presence of chemicals initially barely survive the daily 10X dilution at the start of the experiment, as the initial dose of each chemical was chosen to be close to the maximum possible tolerated whilst avoiding extinction. Under this assumption, the population growth rate per hour for week zero, derived from the equation *N*_*t*_ = *N*_0_*e*^*rt*^, is $r_{0}=\frac{\ln\;(10)}{24}$ (since at time zero a population grows very close to 10-fold per 24 h). The growth rate at week 12 can similarly be estimated as *r*_12_ = ((log_10_*N* − log_10_*N*_0_)*e)/(*t* − *t*_0_), which is the same as *e* times the slope (*m*) from the regression of log10 counts on time in hours. From this, the change in population growth rate (as a proxy for fitness gain) is $\frac{r_{12}}{r_{0}}=24m\;e\;/\ln\;(10)≅28.3m$, where *m* is the slope obtained from the 12-week evolved population. For estimating change in population growth rate for the YPD-only condition, the growth rate at week 0 (*r*_0_) was estimated in the same way, using the same time-points, as for week 12.

*Haplotype calling in Illumina sequenced evolved populations:* A custom in-house haplotype caller was used to impute sliding window founder haplotype frequencies genome-wide, as described in (Linder et al. 2020). Briefly, we slide through the genome in 1 kb steps, considering a 60 kb window for each step. In total, we estimate haplotype frequencies at 11,604 loci spaced every 1 kb throughout the genome. This procedure results in highly accurate haplotype frequency estimates with absolute per haplotype frequency errors of ∼0.01 at the coverages employed in this study (Linder et al. 2020). Only samples with genome-wide coverage greater than or equal to 5x were used for downstream analyses, while the 55 sexual populations that are the focus of this paper received a minimum of 38x genome-wide coverage.

Due to the unique mosaic-like genome structure of many natural yeast isolates (Liti et al. 2009; Schacherer et al. 2009; Peter et al. 2018), there are many tens to hundreds of kilobase-sized regions of the genome over which two or more of the 18 founder haplotypes cannot be distinguished from one another. In the case where two (or more) founder haplotypes cannot be distinguished from one another we are unable to estimate the frequency of those two founders for that region, but the sum of their frequencies is estimated as well as any other haplotype. In our previous work, we thus estimated the frequency of *f* founders that cannot be distinguished for some window as the sum of their frequencies divided by *f*. When looking at regions important in adaptation, in some cases, this averaging approach is misleading, so here we took the different approach of defining new “synthetic founders”. For example, for a region at which founders A11 and A12 cannot be distinguished, we create a founder called “A11A12' for that window and track its frequency. This results in a more accurate picture of how regions are evolving, at the price of tracking a variable number of founder haplotypes for each region of the genome. Over all windows, replicates, and treatments the 23 most common synthetic haplotype combinations account for an average of 94.3% of the observed haplotype frequencies.

*Peak calling:* We created a χ^2^ test statistic to identify regions of the genome most changed due to selection within any given chemical treatment. At any given position (in 1 kb steps) we identified the *K* haplotypes (including possibly synthetic haplotypes) with a starting frequency of at least 0.5% in the base population (on average, ∼10 haplotypes per position meet this cutoff). At that position for the *R* replicate evolved populations within the *k*^th^ haplotype, we calculate a normalized deviate from the expected change in allele frequency as

$$\Delta_{k}=\frac{\bar{\delta_{k}}-0}{\sqrt{\mathrm{var}(\bar{\delta_{k}})}}$$

where $\bar{\delta_{k}}$ is the average change in arcsin square root transformed haplotype frequency between the base and the R evolved replicate populations. Since Δ_*k*_ is distributed as a unit normal, the sum of $\Delta_{k}^{2}$ over the K haplotypes is distributed as a Chi-squared with *k* degrees of freedom. Although $\mathrm{var}(\bar{\delta_{k}})$ is unknown, we assume it is constant over haplotypes and estimate it as:

$$\mathrm{var}(\;\bar{\delta_{k}})\;=\left[\bar{\mathrm{var}(\delta_{k})}+\;R^{*}\;\left[\epsilon_{B}^{2}\;+\;\epsilon_{E}^{2}\right]\right]/R$$

where *ε*_*B*/*E*_ is the average error in the haplotype frequency estimates in the Base or Evolved populations, which we estimate as 0.004 or 0.01, respectively (Linder et al. 2020). That is, the average variance between experimental replicates plus the variance due to haplotype estimation error in the base and evolved populations. The different haplotype frequency errors are due to their being estimated from >2000x coverage sequencing in the base population as opposed to an average 53x in the evolved populations (with a range of 9x to 145x, supplementary fig. S2, Supplementary Material online). However, it is important to note that the 55 evolved populations that are the focus of this paper were sequenced to an average of 112x (with a range of 38x to 867x). Further, we show in (Linder et al. 2020) that the error in haplotype frequency estimation is not strongly affected by sequence coverage, consistent with other such reports in the literature (Tilk et al. 2019). For each of the *k* degree of freedom χ^2^ tests suggested above, we obtain a *P*-value and represent the resulting support for change as a −log10(*P*-value). Since much of the genome for all chemicals is above the nominal significance threshold suggested by the χ^2^ test, we attempted to call local peaks using an algorithm that finds local minima and maxima in a vector with an adjustable threshold (Friedland, 2017). This peak caller uses as input the vector of calculated −log10(*P*) values for each drug treatment, which is calculated at 1 kb steps, and calls local maxima separated by at least 50 steps (or 50 kb) on either side of a local maxima (see github for details). As a result, peaks are at least 100 kb apart.

*Per-site heterozygosity deviation*: The per-site haplotype heterozygosity was calculated at each of the 11,604 loci for the base and each evolved population as one minus the sum of the squared haplotype frequencies. We then calculated the average reduction in per-site heterozygosity by summing the differences in heterozygosity per-site between the base and each evolved population and dividing by the number of loci.

*Classification of evolved replicate populations:* Following the sequencing of 221 evolved populations we observed different types of evolved replicates after 12 weeks of evolution (fig. 2). Closer analysis led us to believe that some populations had been invaded by asexual “cheaters’ that rose to a high population frequency. We believe this was driven by individuals exploiting a strategy in which they did not sporulate and/or randomly mate, while being able to survive our stringent spore isolation protocol. The observed ∼600-fold reduction in the number of mated spores by Monday evening relative to the number of diploids going into sporulation on Friday makes it clear there was a huge fitness cost to “playing by the rules”. We observed two types of cheater populations. One cheater type was characterized by very low per-site heterozygosity and appeared haploid at all but the mating-type locus (where heterozygosity was maintained), with relative sequence coverage supporting haploids having either a second copy of the entire Chromosome III or just the mating-type locus itself. The other cheater type appears to have fixed a single, heterozygous highly recombinant diploid clone (see supplementary Note S1, Supplementary Material online). In both cases, it is possible the clone that came to dominate the population was able to both exploit our scheme for enforcing outcrossing, and perhaps had a mutation of large effect that allowed it to survive the chemical challenge. The remaining populations appear to have evolved in a manner typical of sexual outbreds, with considerable genome-wide heterozygosity and raw haplotype frequencies varying in frequency across the genome. All evolved populations were thus classified into one of three classes: aneuploid haploid, clonal diploid, or outbred sexual. Per-site heterozygosity and genome-wide haplotype frequency profiles were used as metrics for classification. Specifically, populations with per-site heterozygosity profiles close to 0 with a single haplotype fixed genome-wide (except at the mating-type locus) were classified as aneuploid haploids. Populations with a bimodal per-site heterozygosity profile (with peaks centered close to 0 and 0.5) and with per-site haplotype frequencies either fixed for a single haplotype or split evenly between two haplotypes were classified as clonal diploid. The remaining populations were characterized by per-site heterozygosity profiles with a unimodal distribution and negative skew, while haplotype frequencies were highly heterogeneous throughout the genome and were classified as outbred sexual. Finally, we required any given drug to have six non-cheater replicates (except YPD) to allow for statistical inference.

*Determining recombination rate in budding yeast and D. melanogaster:* Two closely related metrics for estimating the recombination rate in *S. cerevisiae* vs. *D. melanogaster* are used. The first estimates the average initial generation recombination rate per generation per Mb. This is calculated as 90*(1/20)/12 = 0.38, due to an estimated 90 cross-overs occurring in budding yeast during meiosis with a single meiosis every ∼20 mitotic generations and a genome size of approximately 12 Mb. For *D. melanogaster*, the sex-averaged recombination rate is calculated as 5*(1/2)/120 = 0.02, due to an estimated five cross-overs occurring per generation in females only and a genome size of approximately 120 Mb. The per-gene recombination rate is estimated similarly, with size of the genome replaced by the number of genes- approximately 14,000 in *D. melanogaster* for a recombination rate of ∼0.0002 per generation per gene as compared to approximately 6,275 genes in budding yeast for a recombination rate of ∼0.0008 per generation per gene. Unlike experiments in obligately sexual flies, in the yeast populations as the experiment progresses and the yeast adapt, they complete more asexual cell divisions per week (or per meiosis) and the above rate drops. For a fitness increase of 5X for example the recombination rate per cell division per week drops to 90 * (1/(5*10 + 10))/12 = 0.125 or about 3-fold (since the fitness increase is likely mostly realized during the asexual growth phase in the presence of drugs).

*Determining sequence-level conservation at potentially functional variants:* Sequence-level conservation information was taken from the UCSC genome browser based on a phylogenetic hidden Markov model (phastCons) comparing seven species of the genus *Saccharomyces*. Sequences were called as highly conserved if the level of conservation was at least 60%.

*Detecting chromosomal or segmental duplications:* To scan for the presence of large-scale duplications within the 55 evolved populations that passed all filters, coverage was determined in 2 kb non-overlapping intervals throughout the genome in the base and evolved populations. The relative coverage of each site was computed and the ratio of relative coverage of the evolved populations over the base population was calculated to determine the normalized fold-coverage at each site. Sites with normalized fold-coverage greater than or equal to 1.25 (which signifies that the relative coverage in the evolved population is at least 25% greater than in the base population at a particular site) were used as input for a Hidden Markov Model to predict the bounds of large, duplicated regions, which was carried out in R using a custom script.

*Detecting* de novo *single-nucleotide variants:* To scan for the presence of de novo mutations in all evolved populations, SNPs found in our evolved populations were considered as potential candidate de novo SNVs if they passed a series of filters, including: excluding SNPs present in any of the original founders or base population, including clones directly derived from the base population (Linder et al. 2020), excluding sites with less than 10x sequencing coverage, and only including SNVs at which at least 20% of the reads were called as the mutant allele. Further, to filter out spurious mutations that may have resulted as artifacts of the culturing conditions in general, mutations that showed up in more than five different chemical treatments were excluded. SNPeff (Cingolani et al. 2012b) and SNPsift (Cingolani et al. 2012a) were used to predict the impact of all de novo mutations detected that occurred in coding regions of annotated genes. Intergenic SNVs were then further annotated based on genomic overlap with features other than genes using the saccharomyces_cerevisiae_R64-2-1_20150113.gff file downloaded from the SGD website. Intergenic SNVs that did not overlap any known annotated genomic features were further analyzed by using them as input for the R package motifbreakR (Coetzee et al. 2015), which predicts whether or not SNPs disrupt predicted transcription factor binding sites. The list of de novo SNVs were finally filtered to only include the 55 evolved populations analyzed throughout this study.

## Supplementary Material

msac248_Supplementary_Data

## Data Availability

Raw sequencing reads have been uploaded to the SRA under BioProject PRJNA748708. We host some useful processed files (SNP and haplotype calls, supplementary tables S3 and S4, Supplementary Material online, and populations included in the study) at: ​​http://wfitch.bio.uci.edu/∼tdlong/sandvox/publications.html. The haplotype caller is described in a previous publication (Linder et al., 2020), but the github is here: https://github.com/tdlong/yeast_resource. The code for creating all figures and tables used in this paper can be found here: https://github.com/rlinder02/Linder-et-al-2022.
