# Supplementary material for: Adaptation in Outbred Sexual Yeast is Repeatable, Polygenic and Favors Rare Haplotypes
Source: Mol Biol Evol. 2022 Nov 11;39(12):msac248. doi: 10.1093/molbev/msac248 (PMC9728589; doi:10.1093/molbev/msac248)
Supplement: msac248_Supplementary_Data [file msac248_supplementary_data.pdf]

Supplementary Information

Table S1. Chemicals and doses used in this study

Chemical	Day 1 dose	Days 2+ dose	General Impact	Effects in budding yeast
Cadmium chloride	400uM	600uM	Widespread environmental contaminant and type I carcinogen	Induces oxidative stress and heavy metal toxicity
Caffeine	16mM	18mM	Widely used legal stimulant that is highly addictive	Inhibits homologous recombination and extends lifespan in yeast by inhibiting RAD51 and mTORC1, respectively
Chlorpromazine	8uM	10uM	Commonly prescribed anti-psychotic with idiosyncratic toxicity	Induces oxidative stress and the unfolded protein response, inhibits protein synthesis, alters membrane integrity, and inhibits intracellular protein trafficking
Cisplatin	400uM	600uM	Frequently used chemotherapuetic agent with high rates of intrinsic and acquired resistance	Induces DNA damage by crosslinking with purine bases
Diamide	2.5mM	2.7mM	Widely used insecticide	Induces oxidative stress
DMSO	8.50%	7%	Widely used organic solvent	Induces oxidative stress
Ethanol	12.50%	12.50%	Alcohol commonly encountered in nature	Induces oxdidative stress
Fluconazole	100uM	5uM	Anti-fungal	Interferes with formation of the cell membrane
Glacial acetic acid	100mM	120mM	Accumulates in yeast culture during stationary phase	Induces oxidative stress and causes intracellular acidification
Sodium chloride	900mM	950mM	Commonly used preservative and food additive	Induces osmotic stress
Nicotinamide	220mM	225mM	NAD+ precursor shown to protect against obeisity and extend lifespan in mice	Protects against DNA damage

Nicotine	7mM	9mM	Widely used legal stimulant that is highly addictive	Induces oxidative stress
Sodium sulphite	85mM	90mM	Used as a preservative in winemaking	Causes ATP depletion
Tunicamycin	0.8uM	1uM	Inhibits tumor cell growth	Inhibits protein glycosylation and formation of N-acetylglucosamine lipid intermediates
Urea	900mM	650mM	Inovolved in nitrogen excretion and used in fertilizers	Denatures proteins
YPD	NA	NA	Rich media used for yeast cell culturing	Supports rapid growth of budding yeast

Table S2. Count of populations per chemical detected in each class

Drug	Outbred sexual	Clonal diploid	Aneuploid haploid
Sodium chloride (NC)	12	1	0
Cadmium chloride (CD)	10	5	0
Chlorpromazine (CP)	11	4	0
Diamide (DI)	10	4	0
Glacial acetic acid (GA)	10	5	0
Flucanozole (FL)	8	8	0
Nicotine (NT)	8	7	0
Nicotinamide (NM)	8	6	1
Urea (UR)	6	7	0
YPD (YP)	6	3	0
DMSO (DM)	5	7	1
Ethanol (ET)	4	9	0
Cisplatin (CI)	3	12	0
Sodium sulfite (SO)	2	11	2
Caffeine (CA)	1	5	6
Tunicamycin (TU)	1	10	2
Average of those kept	9	4	0
Average of those discarded	4	8	1

*Values are rounded to the nearest whole number.

Table S3. Large-scale duplications detected in this study.

Chemical	chr	Interval	Total reps	Replicate(fold change)	Duplication type
CD	II	NA	7	R04(1.4), R05(1.4), R06(1.3), R08(1.4), R09(1.4), R10(1.4), R11(1.2)	whole
CD	III	208,813-300,813	7	R06(1.1)	partial
CD	VIII	522,750-554,750	7	R05(1.9)	partial
CD	VIII	540,750-554,750	7	R04(2.6), R06(2.6), R08(2.5), R09(2.5), R10(2.5), R11(2)	partial
CP	VIII	540,750-554,750	8	R02(2), R03(1.5), R06(1.8), R07(2.2), R08(1.8), R10(2.7)	partial
CP	XVI	920,842-930,842	8	R01(1.3), R02(1.4), R03(1.4), R08(1.3)	partial
DI	I	NA	10	R12(1.1)	whole
DI	III	NA	10	R12(1.1)	whole
DI	III	150,813-226,813	10	R10(1.4)	partial
DI	III	152,813-224,813	10	R16(1.4)	partial
DI	V	559,329-565,329	10	R12(1.4)	partial
DI	V	9,329-49,329	10	R12(1.2)	partial
DI	VI	206,139-264,139	10	R01(2)	partial
DI	VI	206,387-264,387	10	R02(1.9), R03(1.8), R04(2), R05(2), R06(2), R08(1.9), R10(1.9), R16(1.9)	partial
DI	VI	76,387-264,387	10	R12(1.3)	partial

DI	VIII	472,750-554,750	10	R12(1.2)	partial
DI	VIII	522,750-554,750	10	R04(1.8)	partial
DI	VIII	540,750-554,750	10	R02(3), R03(3.2), R05(2.8), R06(2.7), R08(2), R10(2.4), R16(2.1)	partial
DI	IX	NA	10	R12(1)	whole
DI	X	620,954-726,954	10	R12(1.2)	partial
DI	XIII	14,402-110,402	10	R12(1.1)	partial
DI	XIII	656,402-916,402	10	R12(1)	partial
DI	XIV	558,018-776,018	10	R12(1.1)	partial
DI	XV	1,031,245-1,073,245	10	R12(1.2)	partial
DI	XVI	892,842-930,842	10	R12(1.2)	partial
DI	XVI	920,842-940,842	10	R01(1.3)	partial
GA	III	208,813-300,813	9	R16(1.1)	partial
GA	VIII	540,750-554,750	9	R01(2.7), R03(2.7), R04(3.2), R05(2.5), R06(2.3), R07(3.2), R09(2.6), R11(2.8), R16(4)	partial
GA	IX	407,044-431,044	9	R16(1.2)	partial
GA	X	700,954-726,954	9	R16(1.2)	partial
GA	XIV	766,018-776,018	9	R16(1.4)	partial
GA	XVI	12,842-366,842	9	R04(1.2)	partial
GA	XVI	12,842-370,842	9	R06(1.3), R07(1.4), R09(1.4), R11(1.4), R16(1.4)	partial
GA	XVI	924,842-930,842	9	R09(1.6), R16(1.5)	partial
NC	III	NA	10	R11(1.4)	whole

NC	III	198,813-300,813	10	R03(1.4), R04(1.1), R07(1.4)	partial
NC	V	NA	10	R11(1.3)	whole
NC	VIII	524,750-554,750	10	R05(1.9)	partial
NC	VIII	540,750-554,750	10	R01(1.9), R02(2), R03(2.3), R04(3.2), R06(2.6), R07(2.9), R09(2.7), R12(2.6)	partial
NC	IX	377,044-431,044	10	R04(1.3)	partial
NC	XV	1,051,245-1,073,245	10	R04(1.3)	partial
NC	XVI	920,842-930,842	10	R02(1.4)	partial
UR	II	797,487-803,487	6	R15(1.4)	partial
UR	V	NA	6	R08(1.5), R09(1.4), R14(1.3), R15(1.2)	whole
UR	VIII	540,750-554,750	6	R05(2), R08(2.1), R09(2.7), R11(1.8), R14(2.1), R15(2.1)	partial
YP	VIII	522,750-554,750	5	R05(1.6)	partial
YP	VIII	540,750-554,750	5	R02(2), R08(1.9)	partial
YP	XVI	920,842-930,842	5	R08(1.4)	partial

Table S4. Single nucleotide variants detected in this study.

Chemical	chr	Pos	Total reps	Replicate (frequency)	mutation	Mutation type	Gene hit
CD	I	106,071	7	R04(0.2)	C>A	TFBS_Disruption	LTE1
CD	IV	1,924	7	R06(0.2)	T>C	Synonymous	COS7
CD	IV	169,930	7	R10(0.2)	T>A	TFBS_Disruption	MHF2
CD	IV	865,025	7	R04(0.3)	A>T	Missense	UME6
CD	V	520,777	7	R06(0.3)	G>A	Intergenic	ARS521
CD	VI	6,810	7	R05(0.2)	C>T	Synonymous	COS4
CD	VII	611,564	7	R06(0.2)	G>T	Intergenic	ERG25

CD	XI	61,163	7	R09(0.2)	G>T	Missense	TOR2
CD	XII	873,696	7	R11(0.2)	C>T	TFBS_Disruption	PSY3
CD	XIII	472,636	7	R06(0.3)	T>A	TFBS_Disruption	YMR102C
CD	XIII	559,094	7	R06(0.2)	G>A	Intergenic	TIF34
CD	XIII	865,417	7	R10(0.2)	C>T	TFBS_Disruption	DYN3
CD	XIV	196,170	7	R05(0.6)	A>C	Intergenic	ARS1412
CD	XVI	287,581	7	R05(0.2)	A>T	Missense	MKK2
CP	II	166,036	8	R10(0.2)	A>T	TFBS_Disruption	YBL029W
CP	II	251,994	8	R02(0.2)	G>T	TFBS_Disruption	DSF2
CP	II	251,995	8	R02(0.2)	A>T	TFBS_Disruption	DSF2
CP	II	251,999	8	R02(0.2)	G>T	Intergenic	DSF2
CP	II	505,129	8	R02(0.7)	G>A	Synonymous	CKS1
CP	III	1,902	8	R06(0.2)	G>A	Missense	YCL076W
CP	IV	693,336	8	R02(0.3)	G>A	TFBS_Disruption	TRM1
CP	V	492,019	8	R07(0.2)	G>T	TFBS_Disruption	BUR6
CP	IX	415,829	8	R02(0.4)	A>T	TFBS_Disruption	DAL3
CP	X	173,267	8	R06(0.3)	C>A	Intergenic	URA2
CP	X	176,417	8	R02(0.2)	A>T	Missense	TRK1
CP	X	176,418	8	R02(0.2)	A>T	Missense	TRK1
CP	X	193,334	8	R02(0.3)	T>A	Synonymous	PHO86
CP	X	193,335	8	R02(0.2)	T>A	Missense	PHO86
CP	X	193,336	8	R02(0.3)	T>A	Premature_stop	PHO86
CP	X	675,076	8	R10(0.9)	T>C	Synonymous	SGM1
CP	XI	286,208	8	R04(0.3)	A>T	Stop_lost	VMA5
CP	XI	398,709	8	R06(0.3), R08(0.2)	C>A	Missense	MAK11
CP	XI	450,307	8	R01(0.3)	G>A	TFBS_Disruption	YKR005C
CP	XI	489,811	8	R03(0.2)	A>T	TFBS_Disruption	GCN3
CP	XII	122,034	8	R10(0.6)	A>C	Intergenic	PUF3
CP	XII	533,006	8	R02(0.2)	C>T	Missense	ATG26
CP	XII	547,549	8	R10(0.2)	G>A	Missense	NOP56
CP	XII	587,566	8	R02(0.2)	G>A	Missense	YLR224W
CP	XII	1,042,117	8	R10(0.3)	A>T	Premature_stop	RIF2
CP	XIII	225,438	8	R02(0.5)	T>C	TFBS_Disruption	YML6
CP	XIII	677,460	8	R02(0.2)	C>T	Synonymous	HFA1
CP	XIII	677,461	8	R02(0.3)	A>T	Missense	HFA1
CP	XV	37,749	8	R02(0.3)	A>G	Intergenic	YOL153C
CP	XV	227,560	8	R06(0.3)	T>A	TFBS_Disruption	tG(GCC)O1

CP	XV	1,079,939	8	R08(0.3)	T>A	TFBS_Disruption	HSP33
CP	XV	1,079,940	8	R08(0.3)	T>A	TFBS_Disruption	HSP33
CP	XVI	814,209	8	R10(0.2)	G>T	Intergenic	LOA1
DI	I	662	10	R02(0.2)	C>T	Missense	YAL068W-A
DI	I	45,628	10	R01(0.2)	C>T	TFBS_Disruption	ACS1
DI	I	220,215	10	R08(0.4)	C>T	Synonymous	YAR064W
DI	I	226,241	10	R06(0.2)	C>T	Missense	PHO11
DI	II	45,232	10	R05(0.2)	A>T	TFBS_Disruption	ROX3
DI	II	69,628	10	R02(0.3)	C>A	Intergenic	CDC27
DI	II	645,983	10	R01(0.2)	G>A	Intergenic	ERV15
DI	IV	60,293	10	R12(0.3)	C>T	Missense	HBT1
DI	IV	60,294	10	R12(0.3)	G>T	Missense	HBT1
DI	IV	383,478	10	R06(0.3)	T>C	Missense	PRM7
DI	IV	1,442,247	10	R12(0.2)	A>T	Missense	PUF6
DI	IV	1,517,694	10	R02(0.2)	A>T	TFBS_Disruption	IRC4
DI	V	148,091	10	R04(0.2)	G>A	TFBS_Disruption	GIM4
DI	V	386,140	10	R01(0.3)	C>A	TFBS_Disruption	SWI4
DI	V	492,007	10	R01(0.2)	A>T	Intergenic	BUR6
DI	V	516,605	10	R03(0.2)	C>T	Missense	DNF1
DI	VII	687,607	10	R12(0.3)	T>C	TFBS_Disruption	ESP1
DI	VII	1,011,014	10	R01(0.2)	G>A	TFBS_Disruption	RAD2
DI	VIII	66,694	10	R01(0.4)	A>G	Synonymous	OPI1
DI	VIII	266,228	10	R12(0.2)	C>T	Missense	YHR080C
DI	VIII	266,233	10	R12(0.2)	C>T	Synonymous	YHR080C
DI	IX	139,397	10	R12(0.4)	G>C	Intergenic	RHO3
DI	IX	155,254	10	R12(0.2)	A>T	Intergenic	COX5B
DI	IX	204,002	10	R12(0.2)	G>A	Missense	CAB2
DI	IX	210,821	10	R08(0.2)	C>T	TFBS_Disruption	tI(AAU)I2
DI	X	436,041	10	R12(0.2)	T>C	TFBS_Disruption	AVT1
DI	X	533,912	10	R12(0.2)	T>A	TFBS_Disruption	BFA1
DI	X	636,907	10	R05(0.2)	C>T	TFBS_Disruption	YJR111C
DI	X	738,407	10	R10(0.3)	T>A	Missense	MPH3
DI	XI	581,078	10	R04(0.2)	G>A	TFBS_Disruption	YKR075C
DI	XII	545,956	10	R01(0.2)	A>T	TFBS_Disruption	NOP56
DI	XII	751,305	10	R05(0.2)	A>T	Missense	IMH1
DI	XIII	58,787	10	R01(0.3)	A>T	TFBS_Disruption	SEC65

DI	XIII	559,094	10	R16(0.2)	G>A	Intergenic	TIF34
DI	XIII	822,630	10	R08(0.2)	A>T	TFBS_Disruption	PRM15
DI	XIV	79,576	10	R12(0.2)	G>A	Missense	RIM21
DI	XIV	551,173	10	R01(0.2)	A>T	Missense	COG6
DI	XIV	551,174	10	R01(0.2)	G>T	Missense	COG6
DI	XV	908,360	10	R01(0.2)	G>T	Intergenic	ARS1528
DI	XV	930,496	10	R05(0.2)	G>A	TFBS_Disruption	MYO2
DI	XV	958,391	10	R12(0.2)	C>T	Missense	UBC11
DI	XVI	868,344	10	R12(0.3)	T>C	TFBS_Disruption	ORC4
DI	M	43,971	10	R05(0.3)	T>A	TFBS_Disruption	COB-OLI1
GA	I	662	9	R07(0.3)	C>T	Missense	YAL068W-A
GA	I	42,085	9	R09(0.4)	G>A	Intergenic	ARS105
GA	II	7,420	9	R09(0.3)	C>T	TFBS_Disruption	YBL108W
GA	II	28,453	9	R11(0.2)	G>T	Synonymous	YBL100W-C
GA	II	537,692	9	R11(0.2)	C>A	Intergenic	YSW1
GA	V	141,253	9	R16(0.2)	T>A	Intergenic	YEL008C-A
GA	VI	107,628	9	R06(0.2)	A>T	TFBS_Disruption	YFL015C
GA	VII	121,047	9	R09(0.2)	T>A	Intergenic	MCM6
GA	VIII	324,276	9	R16(0.2)	G>A	Missense	GRE3
GA	VIII	370,610	9	R09(0.3)	A>T	TFBS_Disruption	ECM14
GA	IX	231,393	9	R16(0.2)	T>A	Intergenic	MAM33
GA	IX	310,742	9	R16(0.3)	G>T	TFBS_Disruption	YKE4
GA	IX	391,333	9	R07(0.6)	A>G	Synonymous	FLO11
GA	IX	407,727	9	R05(0.8)	A>G	TFBS_Disruption	DAL1
GA	X	461,703	9	R16(0.3)	T>A	TFBS_Disruption	TMA22
GA	X	537,895	9	R16(0.2)	C>T	Intergenic	YJRCdelta16
GA	X	580,729	9	R16(0.2)	G>T	Premature_stop	AIM24
GA	XI	219,865	9	R16(0.7)	A>C	Intergenic	VPH2
GA	XI	635,512	9	R11(0.2)	G>T	Intergenic	UBP11
GA	XII	11,774	9	R04(0.2)	C>T	Missense	YLL065W
GA	XIII	163,384	9	R16(0.3)	C>A	Intergenic	IMD4
GA	XIV	753,650	9	R16(0.2)	G>T	Missense	YNR065C
GA	XV	21,713	9	R09(0.2)	T>A	Intergenic	ENB1
GA	XV	444,518	9	R04(0.2)	A>T	TFBS_Disruption	YOR062C
GA	XV	470,717	9	R05(0.2)	G>T	Missense	SKI7
GA	XV	711,067	9	R16(0.2)	G>T	Missense	PEX27
GA	XV	711,068	9	R16(0.2)	A>T	Missense	PEX27
GA	XV	908,360	9	R09(0.3)	G>T	Intergenic	ARS1528

GA	XV	908,361	9	R09(0.2)	G>T	Intergenic	ARS1528
GA	XV	1,079,760	9	R05(0.3)	G>A	Intergenic	HSP33
GA	XVI	198,563	9	R16(0.2)	C>A	Intergenic	MRN1
NC	I	105,416	10	R09(0.2)	G>T	Missense	LTE1
NC	II	407,753	10	R05(0.2)	C>T	TFBS_Disruption	UBC4
NC	II	477,371	10	R12(0.3)	C>A	TFBS_Disruption	TKL2
NC	III	150,045	10	R04(0.7)	C>T	Intergenic	tM(CAU)C
NC	IV	99,505	10	R04(0.3)	T>G	Intergenic	TRM8
NC	IV	169,868	10	R06(0.2)	T>A	Intergenic	MHF2
NC	IV	551,805	10	R06(0.3)	G>A	TFBS_Disruption	HEM12
NC	IV	688,415	10	R04(0.4)	A>G	Synonymous	VBA4
NC	IV	1,254,549	10	R01(0.2)	G>T	Intergenic	SAC7
NC	IV	1,523,881	10	R06(0.2)	A>C	Intergenic	PAU10-YDR543C
NC	V	86,707	10	R09(0.2)	A>T	Intergenic	YEL034C-A
NC	V	148,091	10	R03(0.3)	G>A	TFBS_Disruption	GIM4
NC	V	274,150	10	R05(0.3)	T>A	Intergenic	FCY21
NC	VI	4,981	10	R12(0.3)	G>A	Intergenic	YFL063W
NC	VII	627,235	10	R06(0.2)	C>T	Synonymous	YGR069W
NC	VII	784,148	10	R02(0.2)	G>A	Intergenic	ECL1
NC	VII	965,741	10	R04(0.2)	A>T	TFBS_Disruption	YGR237C
NC	VII	1,072,826	10	R09(0.2)	C>A	TFBS_Disruption	MAL13-MAL11
NC	VIII	456,255	10	R03(0.2)	C>A	Intergenic	YHR177W
NC	VIII	528,898	10	R01(0.5)	G>A	Intergenic	FLO5-YHR212C
NC	IX	241,023	10	R07(0.2)	G>A	Intergenic	RNR3
NC	X	90,461	10	R12(0.5)	A>C	TFBS_Disruption	ATG27
NC	X	196,100	10	R06(0.3), R12(0.3)	A>T	TFBS_Disruption	ASF1
NC	X	389,869	10	R04(0.2)	A>T	Missense	VPS53
NC	X	525,764	10	R03(0.3)	T>A	TFBS_Disruption	ANB1
NC	X	586,416	10	R05(0.2)	C>A	Missense	YJR087W
NC	XI	1,436	10	R04(0.3)	A>G	Intergenic	YKL223W
NC	XI	255,762	10	R04(0.5)	T>C	Synonymous	UTP11
NC	XII	51,985	10	R11(0.3)	G>A	Intergenic	FPS1
NC	XII	86,696	10	R12(0.2)	G>A	TFBS_Disruption	ISA1
NC	XII	97,693	10	R04(0.7)	G>A	TFBS_Disruption	SSA2

NC	XII	150,971	10	R09(0.3), R11(0.5)	A>T	Intergenic	ARS1208
NC	XII	150,974	10	R09(0.2), R11(0.4)	A>T	Intergenic	ARS1208
NC	XII	683,211	10	R07(0.3)	G>A	Missense	YLR271W
NC	XII	845,918	10	R04(0.2)	G>T	Intergenic	VPS38
NC	XII	875,494	10	R01(0.2)	C>T	TFBS_Disruption	FBP1
NC	XIII	234,792	10	R12(0.3)	C>T	Synonymous	YML018C
NC	XIII	276,372	10	R12(0.2)	G>A	Missense	TAF4
NC	XIII	371,021	10	R04(0.2)	A>T	Intergenic	ARS1312
NC	XIII	775,210	10	R11(0.2)	C>T	TFBS_Disruption	YMR252C
NC	XIII	822,630	10	R12(0.3)	A>T	TFBS_Disruption	PRM15
NC	XIII	892,720	10	R02(0.2)	G>A	TFBS_Disruption	PSE1
NC	XIV	172,410	10	R04(0.3)	G>A	Intergenic	MRPL17
NC	XIV	172,411	10	R04(0.2)	G>A	TFBS_Disruption	MRPL17
NC	XIV	174,753	10	R04(0.2)	G>T	TFBS_Disruption	NRD1
NC	XIV	191,318	10	R02(0.2)	G>T	Intergenic	ATG2
NC	XIV	573,696	10	R03(0.2)	A>T	Missense	YNL033W
NC	XIV	690,093	10	R04(0.2)	G>T	TFBS_Disruption	SOL1
NC	XIV	758,691	10	R09(0.6)	G>C	Missense	DSE4
NC	XV	243,362	10	R09(0.3)	C>T	TFBS_Disruption	LDS2
NC	XV	348,674	10	R09(0.2)	C>A	TFBS_Disruption	TIR2-AUS1
NC	XV	759,543	10	R05(0.2)	A>T	Intergenic	snR35
NC	XVI	352,487	10	R04(0.2)	A>T	TFBS_Disruption	SSE1
NC	XVI	381,201	10	R06(0.2)	A>T	TFBS_Disruption	RLM1
NC	XVI	835,344	10	R09(0.2)	C>T	Intergenic	PIN3
NC	M	37,760	10	R11(0.6)	A>G	Missense	BI2
UR	II	347,317	6	R09(0.3)	C>T	TFBS_Disruption	PRP6
UR	IV	210,699	6	R05(0.2)	G>T	TFBS_Disruption	RPO21
UR	IV	561,086	6	R09(0.3)	G>T	Intergenic	DBF4
UR	IV	1,061,955	6	R09(0.2)	C>T	Synonymous	PRO1
UR	VII	717,188	6	R09(0.2)	C>T	TFBS_Disruption	SHY1
UR	VII	1,063,279	6	R05(0.2)	C>A	Intergenic	ZUO1
UR	X	151,178	6	R09(0.2)	C>A	Premature_stop	RPB4
UR	X	579,823	6	R09(0.2)	G>A	Missense	BNA2
UR	X	716,443	6	R09(0.3)	C>T	Intergenic	DAN4

UR	X	716,444	6	R09(0.3)	C>T	Intergenic	DAN4
UR	X	716,445	6	R09(0.2)	C>T	Intergenic	DAN4
UR	XI	160,597	6	R09(0.2)	A>T	Missense	RSM22
UR	XI	321,880	6	R09(0.3)	A>T	Intergenic	YKL063C
UR	XI	398,709	6	R08(0.3)	C>A	Missense	MAK11
UR	XII	517,858	6	R05(0.2)	G>A	Intergenic	VTA1
UR	XII	751,305	6	R09(0.2)	A>T	Missense	IMH1
UR	XII	911,891	6	R09(0.3)	A>T	Missense	VPS33
UR	XIII	5,743	6	R05(0.4)	T>C	TFBS_Disruption	YML133C-COS3
UR	XIII	26,589	6	R15(1)	A>T	TFBS_Disruption	PHO84
UR	XIII	772,685	6	R05(0.2)	C>T	Intergenic	ARS1328
UR	XIV	552,709	6	R09(0.3)	G>A	Intergenic	COG6
UR	XIV	782,992	6	R08(0.2)	G>A	TFBS_Disruption	PAU6-YNR077C
UR	XVI	17,238	6	R09(0.2)	A>T	Intergenic	YPL277C
UR	XVI	469,773	6	R09(0.3)	G>A	TFBS_Disruption	NOP4
UR	XVI	774,801	6	R09(0.2)	C>T	Missense	CLB5
YP	III	173,565	5	R02(0.7)	T>C	TFBS_Disruption	RIM1
YP	III	302,896	5	R08(0.3)	G>C	Synonymous	YCR101C
YP	IV	163,869	5	R03(0.5)	C>T	Synonymous	FAP7
YP	IV	212,555	5	R08(0.3)	C>T	Intergenic	ARS409
YP	IV	838,154	5	R04(0.2)	C>T	TFBS_Disruption	SLY1
YP	IV	838,155	5	R04(0.2)	A>T	TFBS_Disruption	SLY1
YP	IV	838,156	5	R04(0.2)	G>T	Intergenic	SLY1
YP	IV	1,523,881	5	R08(0.4)	A>C	Intergenic	PAU10-YDR543C
YP	IX	217,788	5	R03(0.3)	A>T	TFBS_Disruption	RPN2
YP	X	524,681	5	R08(0.2)	C>A	TFBS_Disruption	tS(AGA)J
YP	XI	266,177	5	R04(0.3)	A>T	Missense	BUD2
YP	XIII	420,846	5	R03(0.3)	A>T	Intergenic	PDS5
YP	XIII	420,847	5	R03(0.3)	A>T	Intergenic	PDS5
YP	XIII	865,417	5	R08(0.2)	C>T	TFBS_Disruption	DYN3
YP	XIV	118,963	5	R02(0.3)	G>T	Intergenic	YNL276C
YP	XIV	576,646	5	R03(0.2), R04(0.3)	C>T	TFBS_Disruption	HHT2
YP	XV	1,048,427	5	R04(0.7)	T>C	Intergenic	ATF1-AMF1

Table S5. Percent of genome with a LOD score greater than 5 per chemical

Chemical	LOD > 5
cadmium chloride	98.8
chlorpromazine	100
diamide	91.4
glacial acetic acid	98.4
sodium chloride	93.4
urea	88.3
YPD	99.9

Table S6. Top 3 peaks detected in each chemical treatment.

chemical	ID	chr	start (bp)	length (kb)	# genes	LOD at peak	candidate gene
CD	CD1	II	795500	5	3	129	**PCA1**
UR	UR1	III	37800	5	5	26	NA
NC	NC1	IV	535100	10	5	130	**ENA1**
UR	UR2	IV	619100	9	5	47	YDR090C
CP	CP1	IV	620100	6	2	212	YDR089W
CD	CD2	IV	622100	4	2	66	**YDR089W**
GA	GA1	V	116300	7	10	56	NA
YP	YP1	V	117300	9	11	72	NA
GA	GA2	VII	484700	9	6	65	NA
DI	DI1	VIII	107800	9	10	70	**QCR10**
NC	NC2	VIII	111800	5	7	117	GPA1
CP	CP2	VIII	113800	3	6	171	GPA1
DI	DI2	IX	425000	2	1	59	**YIRCdelta6**
NC	NC3	X	170000	4	2	231	**TRK1**
CP	CP3	X	171000	3	2	371	NA

YP	YP2	X	171000	4	2	121	NA
GA	GA3	XIV	497000	5	6	53	NA
DI	DI3	XV	265200	4	3	96	**SIL1**
UR	UR3	XV	1067200	2	1	34	**PHR1**
CD	CD3	XVI	164800	4	3	71	**AFT2**
YP	YP3	XVI	588800	2	1	72	**YPR015C**

* Bolded genes have previously been shown to be involved in the response to the specific chemical they were detected in or are the only annotated feature in the interval.
** Underlined genes have functions closely related to the chemical of interest.

Table S7. Potentially pleiotropic regions

<table>
<tr><th>ID</th><th>chemicals</th><th>chr</th><th>start</th><th>interval(kb)</th><th>genes</th></tr>
<tr><td>PL1</td><td>GA, NC</td><td>IV</td><td>503100</td><td>13</td><td>RAD28|MIX14|PST2|ARS450|MRH1|LYS14|YDRCdelta2|YDRCdelta3|YDRCdelta4|YDRCTy2-1|YDR034C-D</td></tr>
<tr><td>PL2</td><td>CD, CP, UR, DI, NC</td><td>IV</td><td>622100</td><td>4</td><td>YDR089W|YDR090C</td></tr>
<tr><td>PL3</td><td>GA, YP</td><td>V</td><td>105300</td><td>21</td><td>YEL025C|RIP1|YEL023C|GEA2|URA3|YEL020C-B|TIM9|RPR1|YELCtau1|YEL020C|MMS21|YEL018C-A|EAF5|PMP2|GTT3|NPP2</td></tr>
<tr><td>PL4</td><td>GA, YP, DI, NC</td><td>VII</td><td>497700</td><td>7</td><td>EFM5|SWC4|CUL3|PEX31|TFG2</td></tr>
<tr><td>PL5</td><td>CP, DI, NC</td><td>VIII</td><td>102800</td><td>17</td><td>YHL002C-A|HSE1|RPL14B|CEN8|OSH7|QCR10|LEU5|TCD1|NEM1|GPA1|TIM10|tT(AGU)H|ARS806|YHRCdelta3|YHRCdelta4|STP2</td></tr>
<tr><td>PL6</td><td>CP, YP, NC</td><td>X</td><td>162000</td><td>17</td><td>YJL132W|AIM23|URA2|TRK1|PBS2</td></tr>
</table>

PL7	CP, YP, NC	XVI	439800	23	YPLCTy4-1|YPL060C-A|YPLCtau2|GRX5|PDR12|SUR1|LCL1|LGE1|LEE1|ARS1633|KTR6|OAZ1|ARL3|MNN9|DIG1

Supplemental Figures

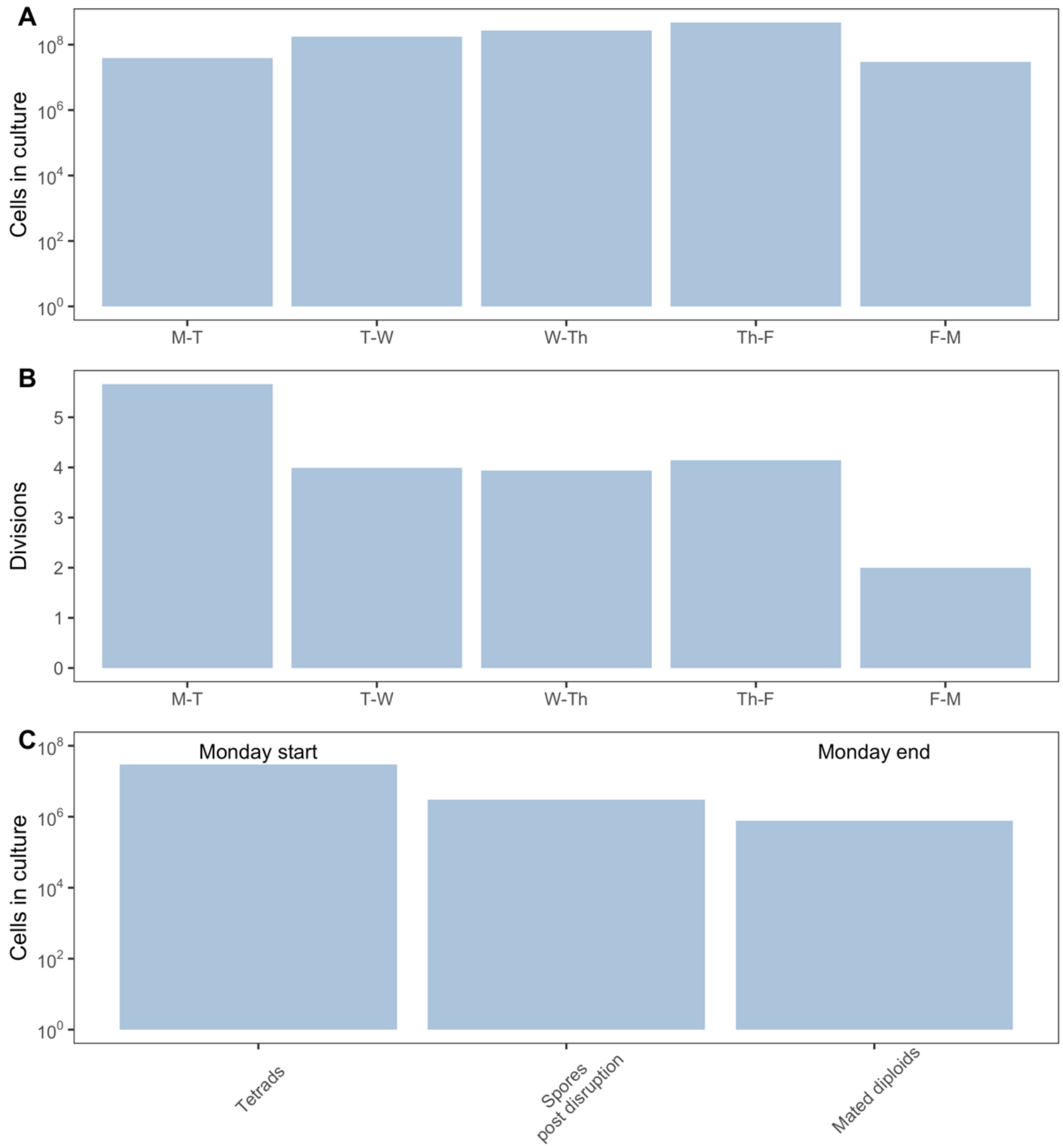

Figure S1. Panel (A) shows the estimated number of cells present in a YPD culture at the time of transfer going into the next day for a week of evolution. The number of mitotic and meiotic generations estimated to occur each day are shown in panel (B). One mitotic and one meiotic generation occur from Friday to Monday. Panel (C) shows the estimated total number of tetrads/cells present in a population at different stages of the Monday regimen, starting with the initial sporulated culture, then the viable spores remaining after killing unsporulated cells, followed by the viable diploids which result after random mating of isolated spores (methods). The vigorous disruption of unsporulated cells leads to a dramatic loss of viable cells (~10-fold), with an additional ~4-fold loss after random mating; nonetheless, over 750,000 viable individuals are left by the end of the day, which is our estimate of the population bottleneck during a typical week of evolution.

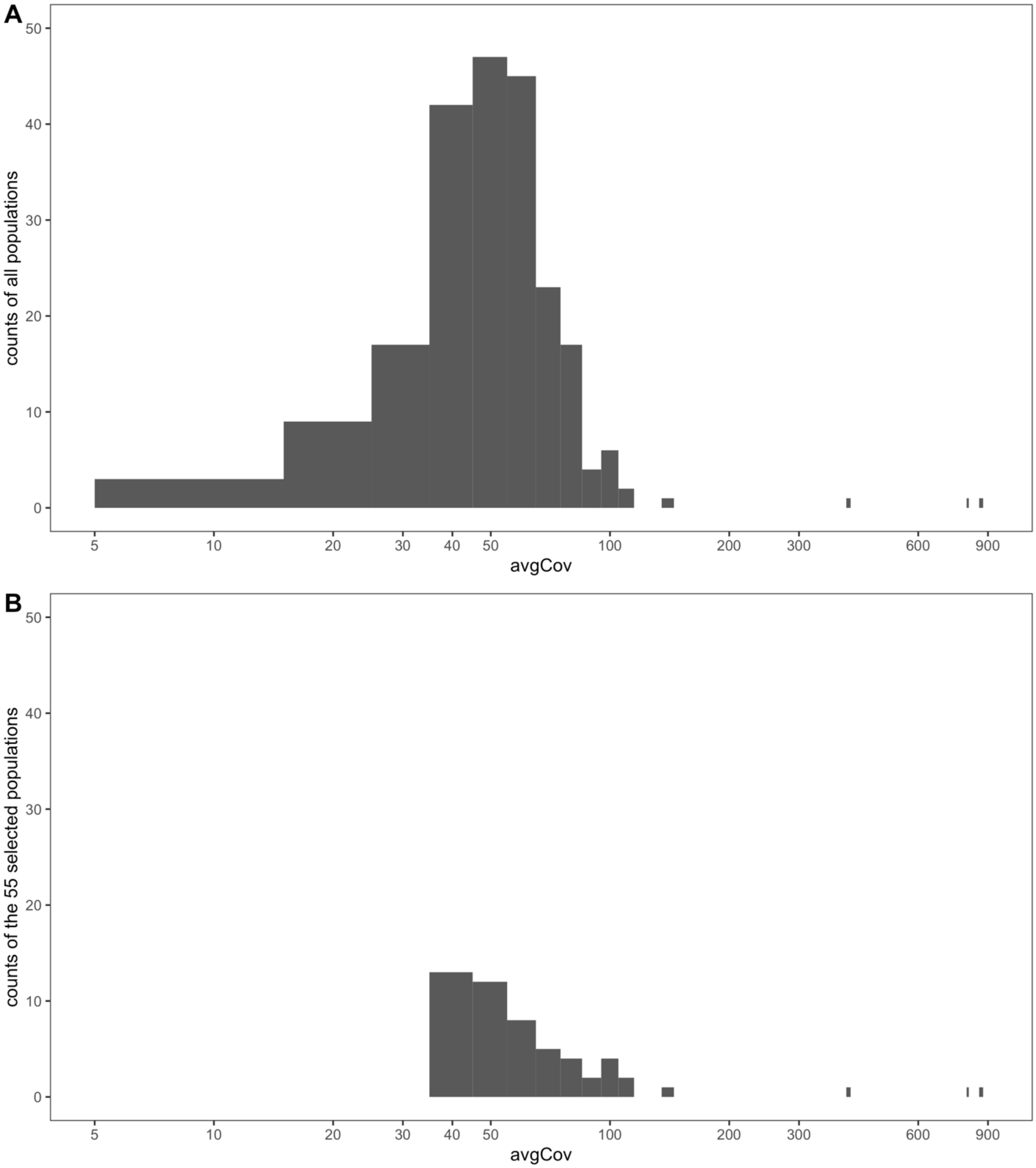

Figure S2. Average genome-wide short read coverage over all populations (excluding the base which was sequenced at 2226X coverage) is shown in Panel (A), while Panel (B) shows coverage for the 55 outbred sexual populations that are the focus of this paper (methods).

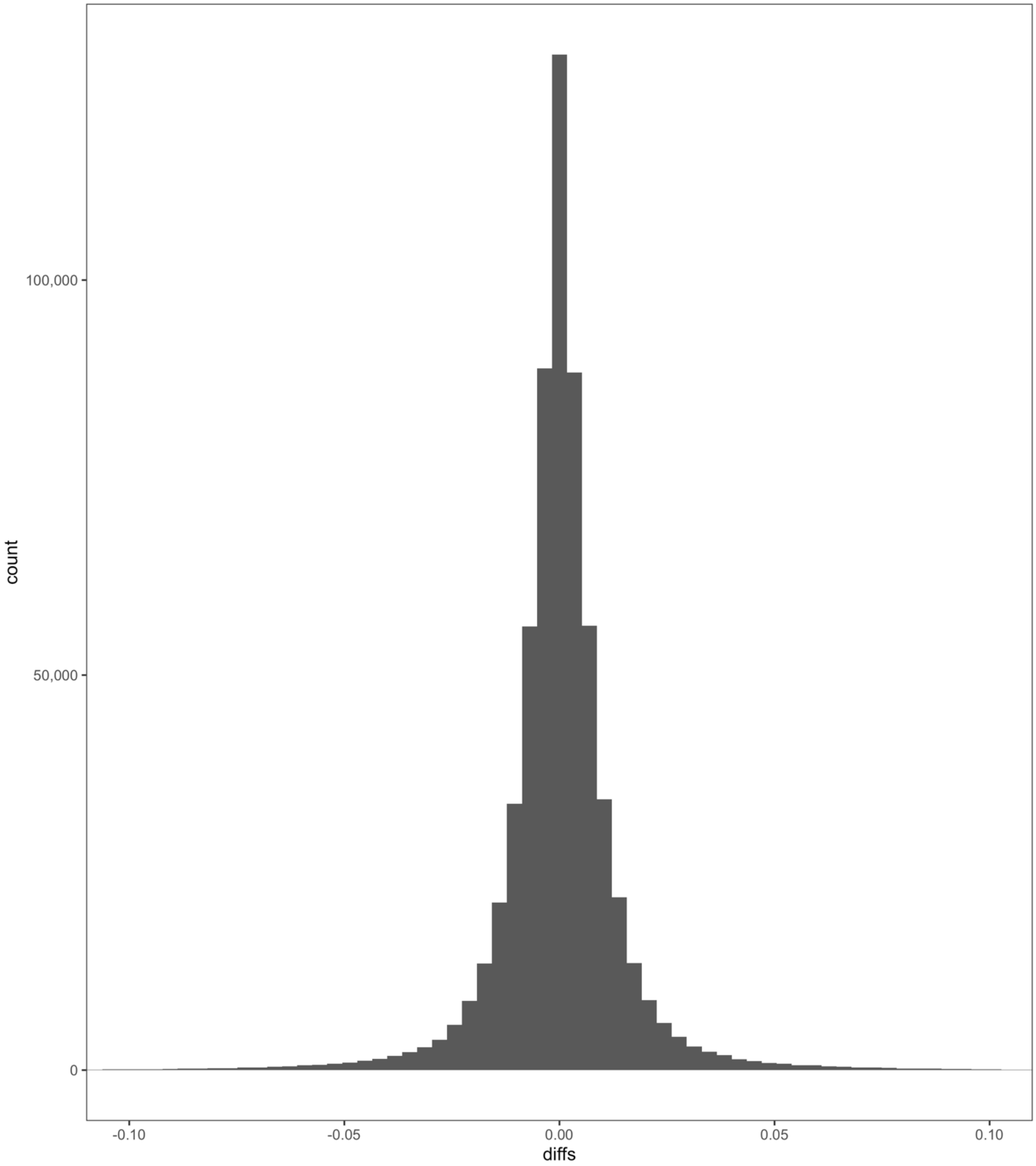

Figure S3. Haplotype frequency change between all adjacent intervals estimated for founder AB3. Only the 55 populations classified as outbred sexual and that clustered together from the seven chemicals used in downstream analyses were used for this analysis.

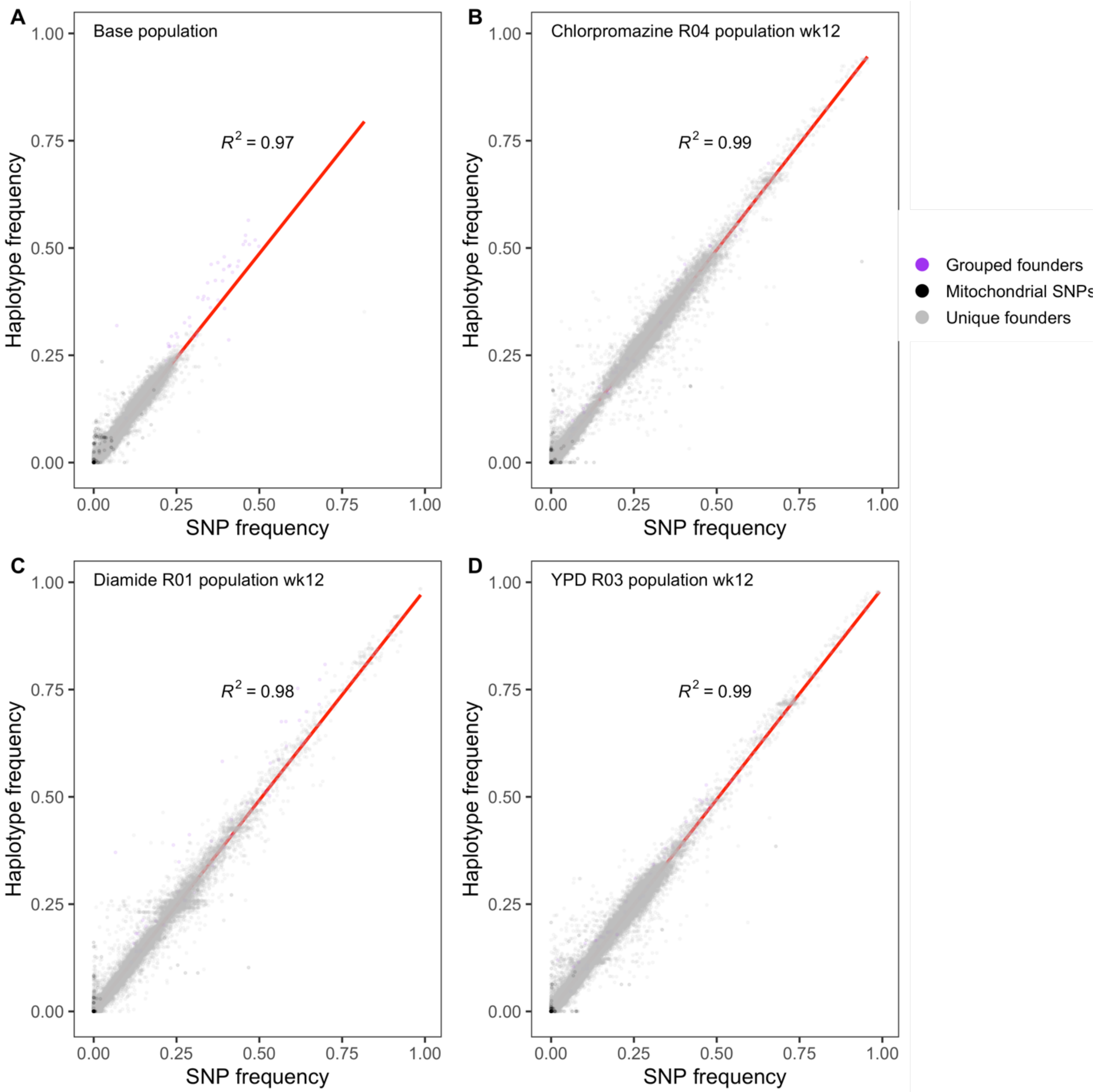

Figure S4. The correlation between inferred haplotype frequencies and the frequencies of SNPs private to a single founder is shown for the base population (panel A), a week 12 chlorpromazine population (panel B), a week 12 diamide population (panel C), and a week 12 YPD-only population (panel D).

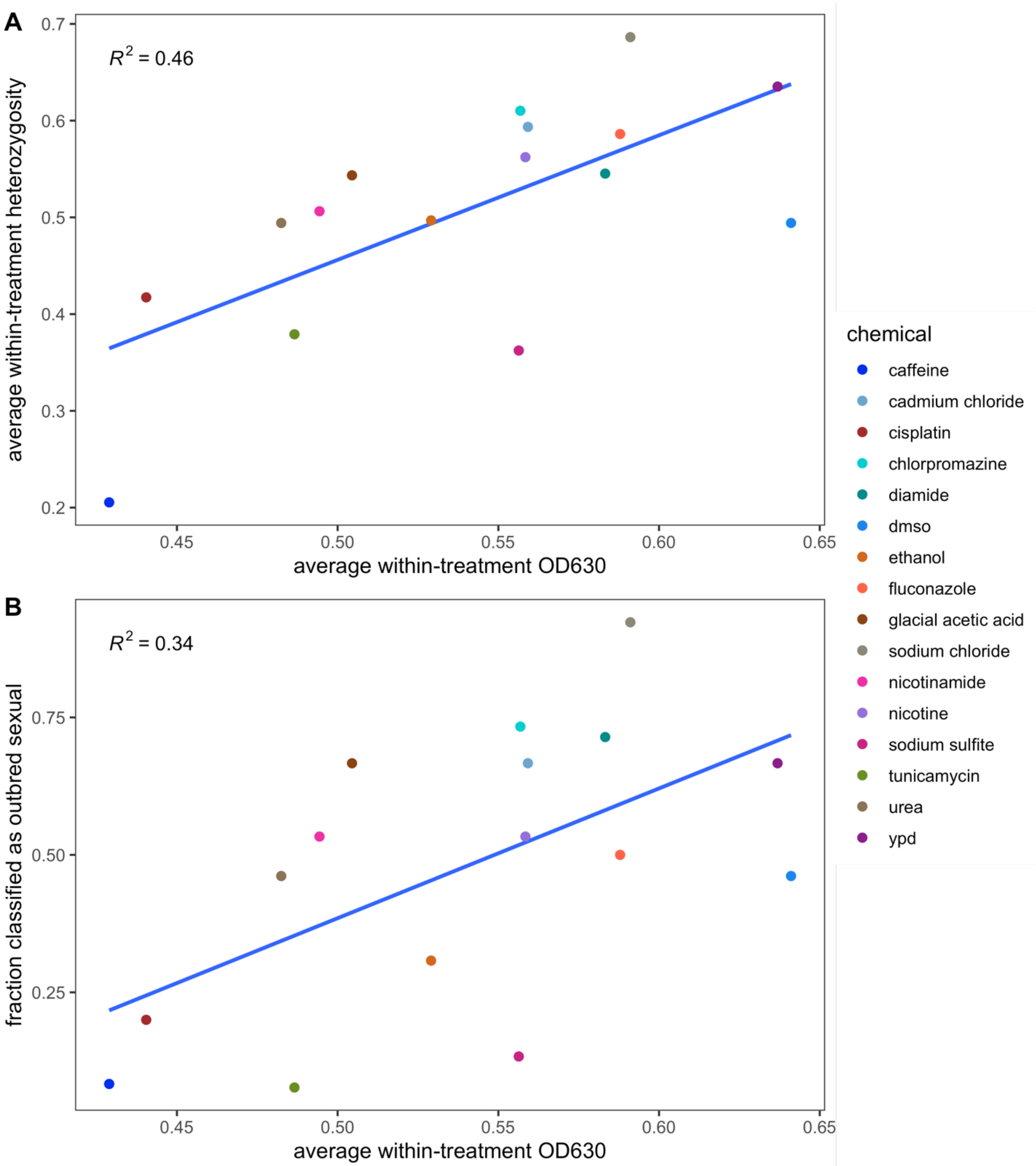

Figure S5. The 12-week straight average OD630 plotted against the mean per-site heterozygosity at week 12 (panel A) and the fraction of populations classified as outbred sexual (panel B) for each chemical is shown. A simple linear model regressing heterozygosity or classification on OD630 is shown. OD630 measurements were taken once a week on Thursdays for all cultures after between 23-25h of growth, at which point cultures had not yet become fully saturated.

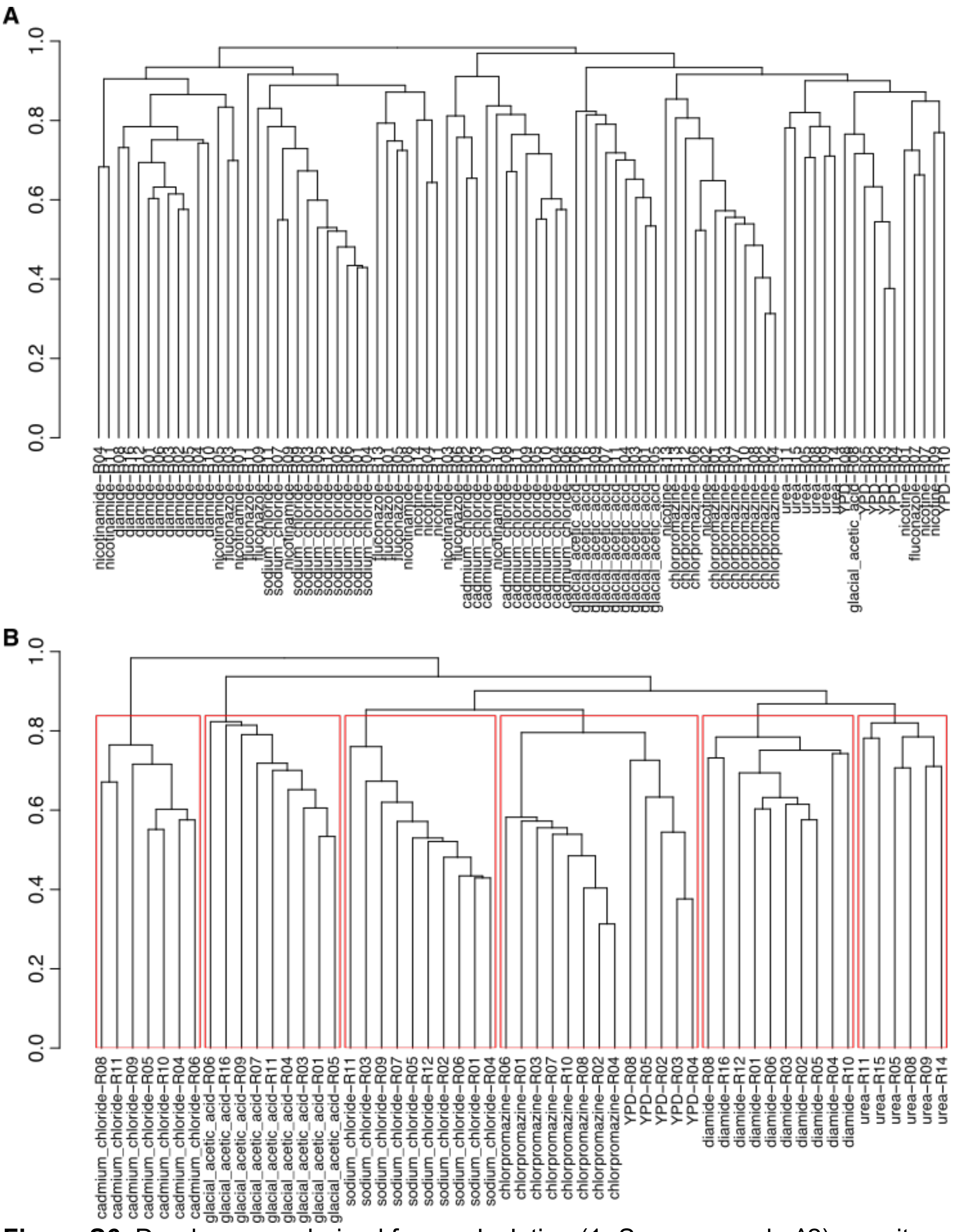

Figure S6. Dendrograms derived from calculating (1- Spearmans rho^2) per-site between each pair of replicates across all chemical treatments. Panel (A) shows replicates that passed the initial heterozygosity filter in chemicals with at least 6 outbred sexual replicates left, while panel (B) shows replicates that could be clustered into distinct groups by chemical stressor.

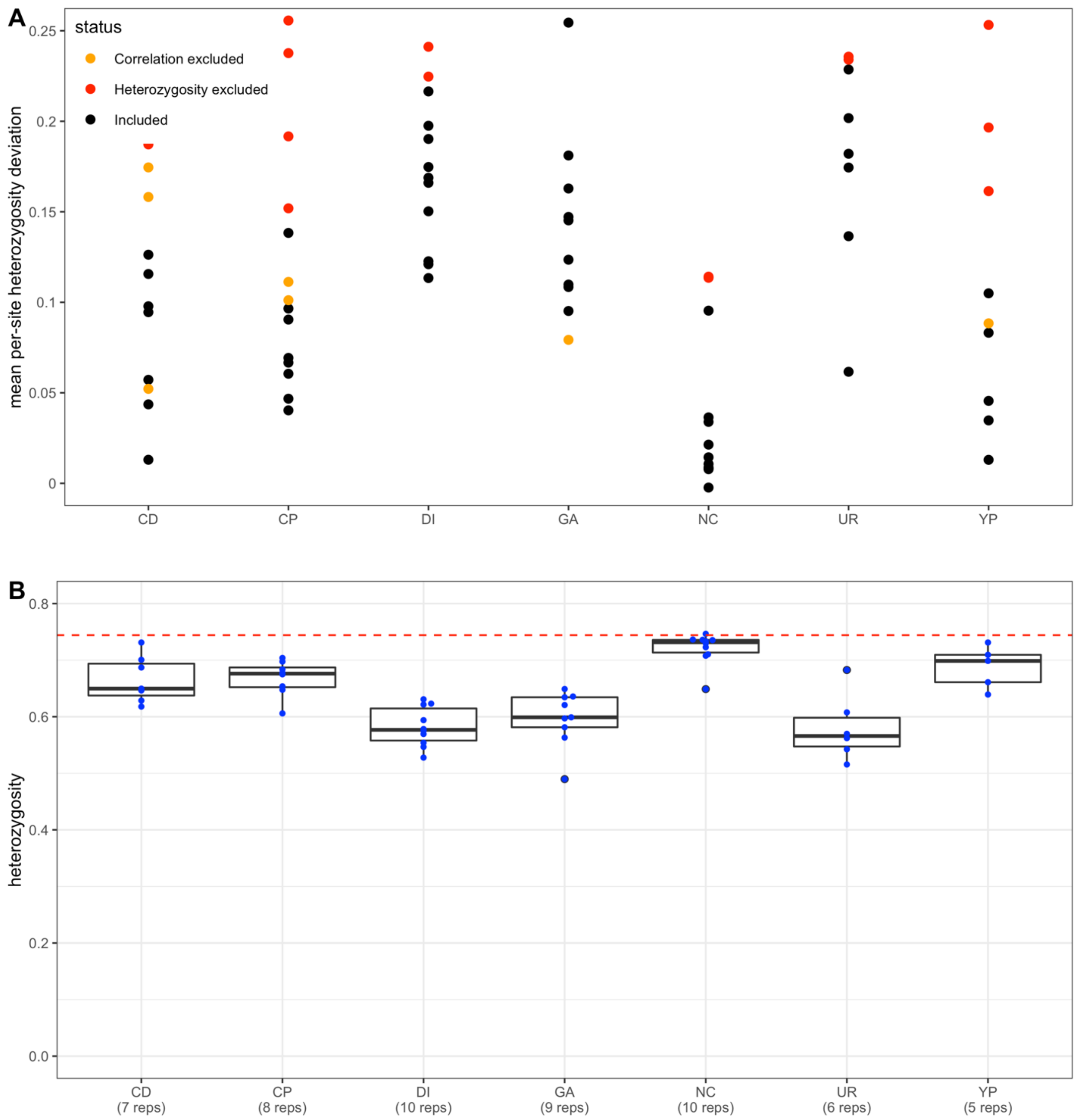

Figure S7. Panel (A) depicts the mean per-site heterozygosity deviation of each replicate population across the seven chemicals analyzed in this study. Replicates excluded due to either abnormal per-site heterozygosity profiles or a lack of correlation with the majority of replicates within a chemical treatment are colored red and orange, respectively. Panel (B) shows the mean per-site heterozygosity of each replicate population for each chemical treatment, with the dashed red line depicting the mean per-site heterozygosity of the base population.

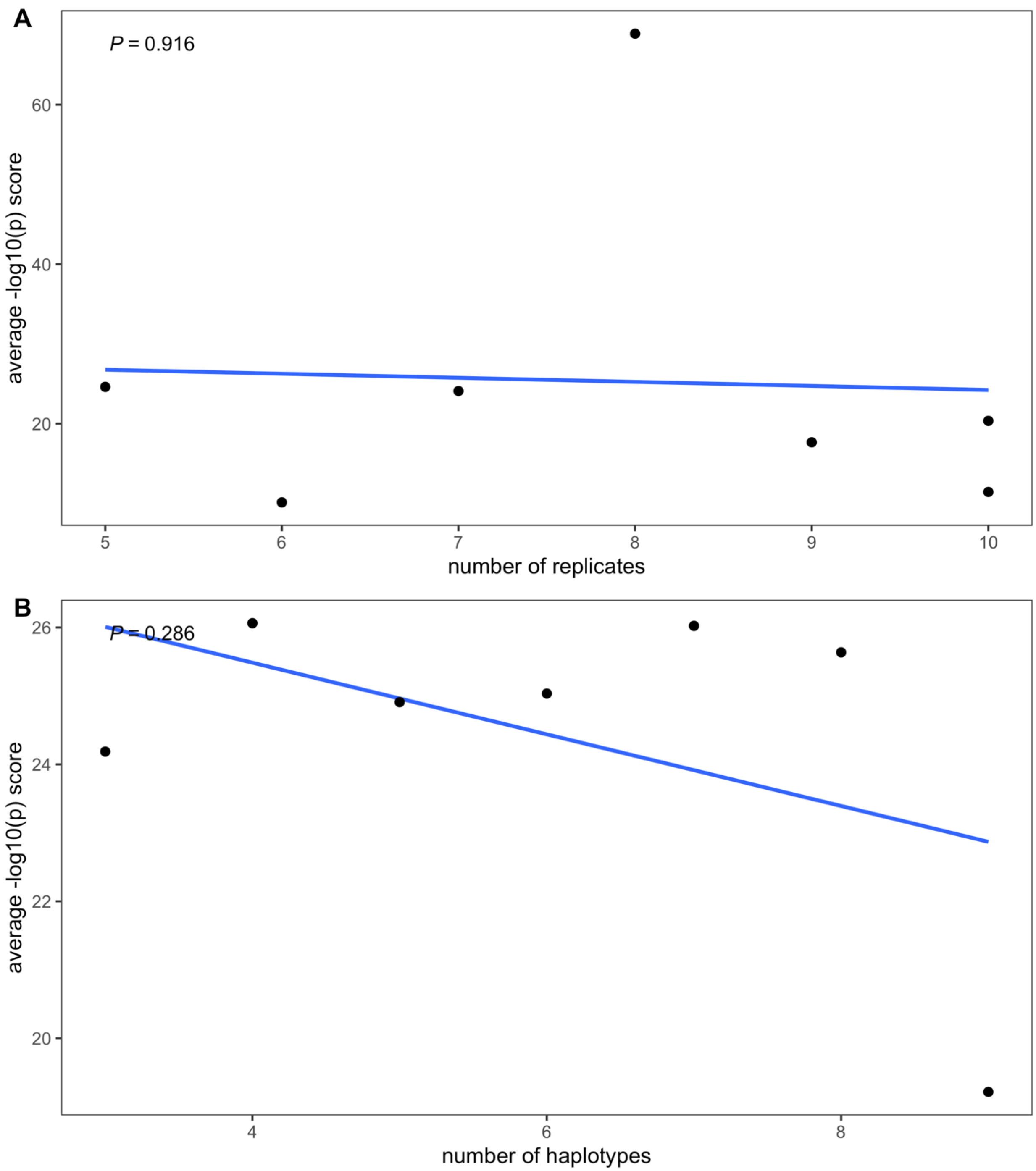

Figure S8. Panel (A) shows the relationship between the number of replicate populations and the average genome-wide LOD score, calculated by regressing the average LOD score on the number of replicates for each chemical. Panel (B) shows the result of a similar analysis regressing average LOD score on the number of haplotypes included for analysis at each locus over all 7 chemicals.

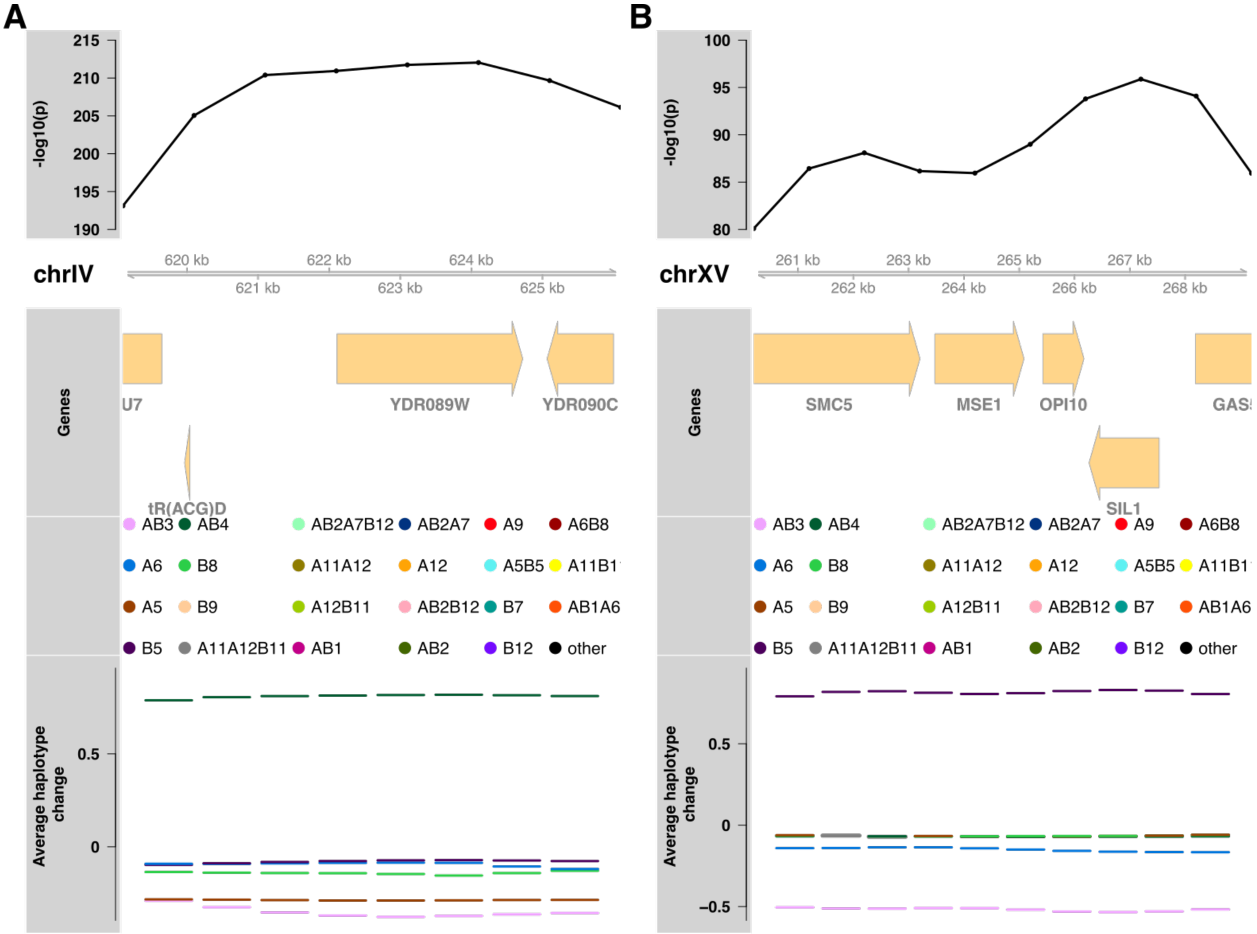

Figure S9. Panel (A) shows genes in a 8kb region underlying a top 3 peak in chlorpromazine and panel (B) a 10 kb region for a top 3 peak in diamide.

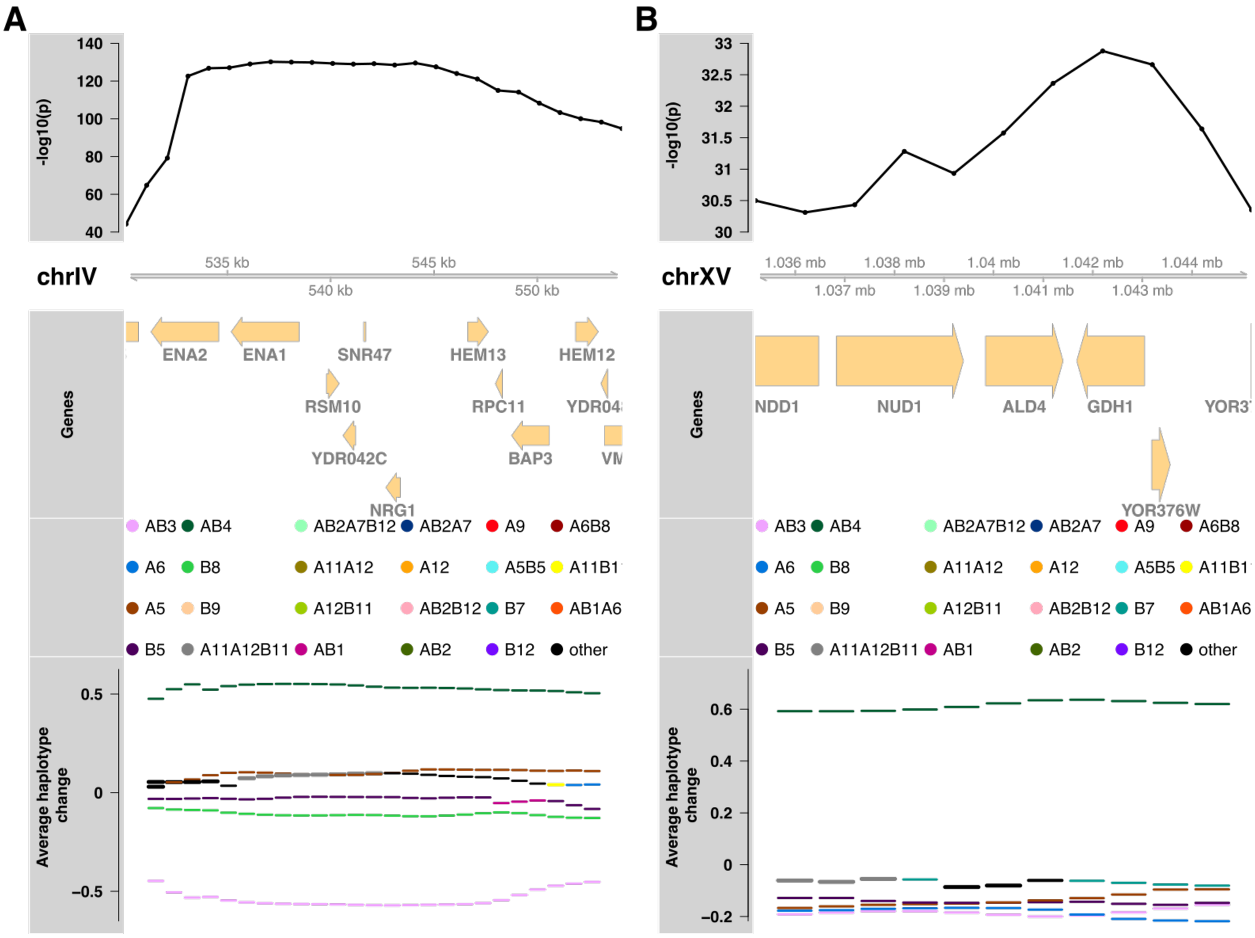

Figure S10. Panel (A) shows genes in a 25kb region underlying a top 3 peak in sodium chloride and panel (B) a 11kb region for a peak in urea.

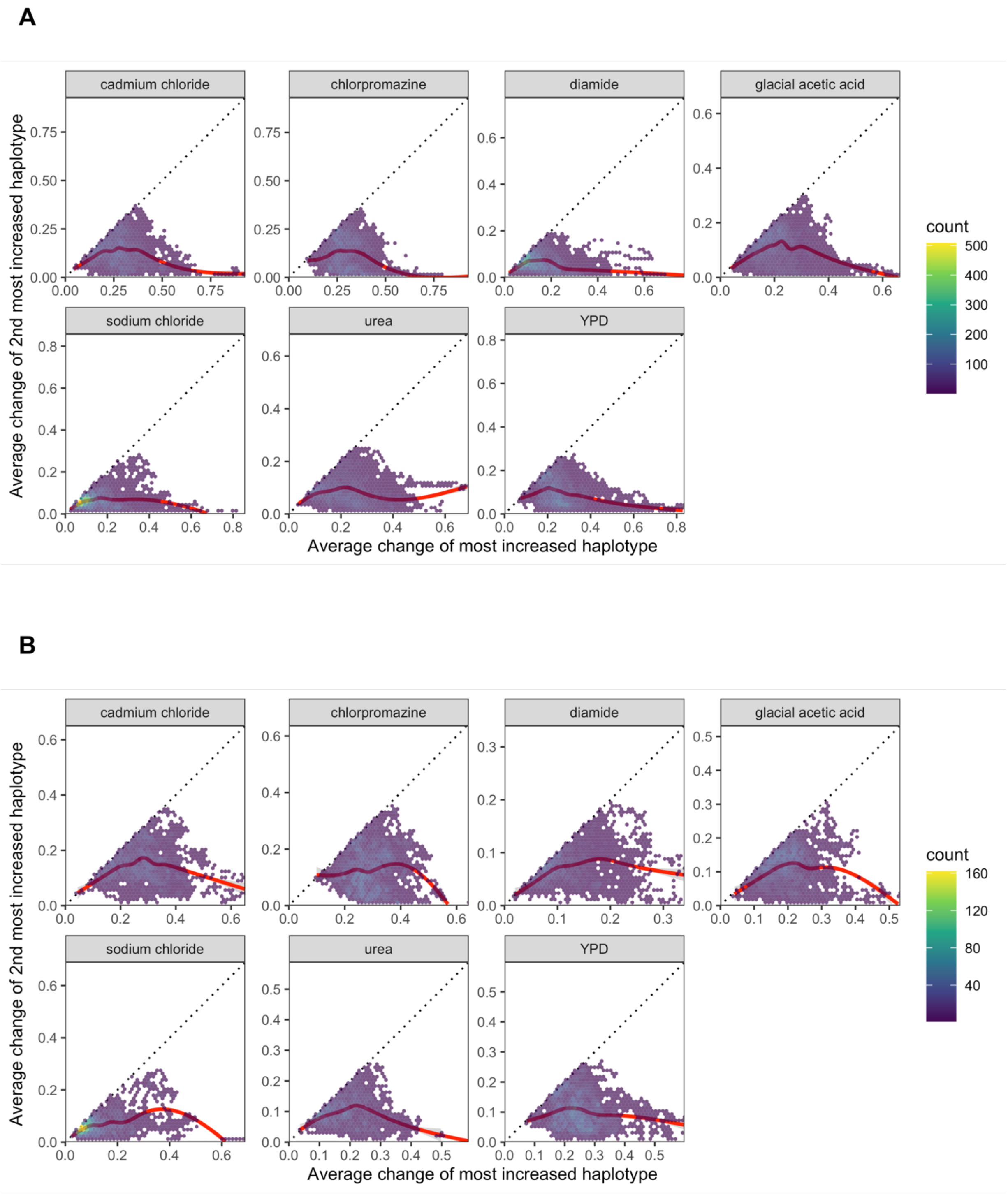

Figure S11. Panel (A) shows the average change of the Most Increased Haplotype (MIH) vs that of the second most increased haplotype within each chemical treatment across all loci. Panel (B) is similar except that only loci at which the second most increased haplotype was at a higher starting frequency in the base population than the MIH are plotted. Loess smoothing is represented as red lines.

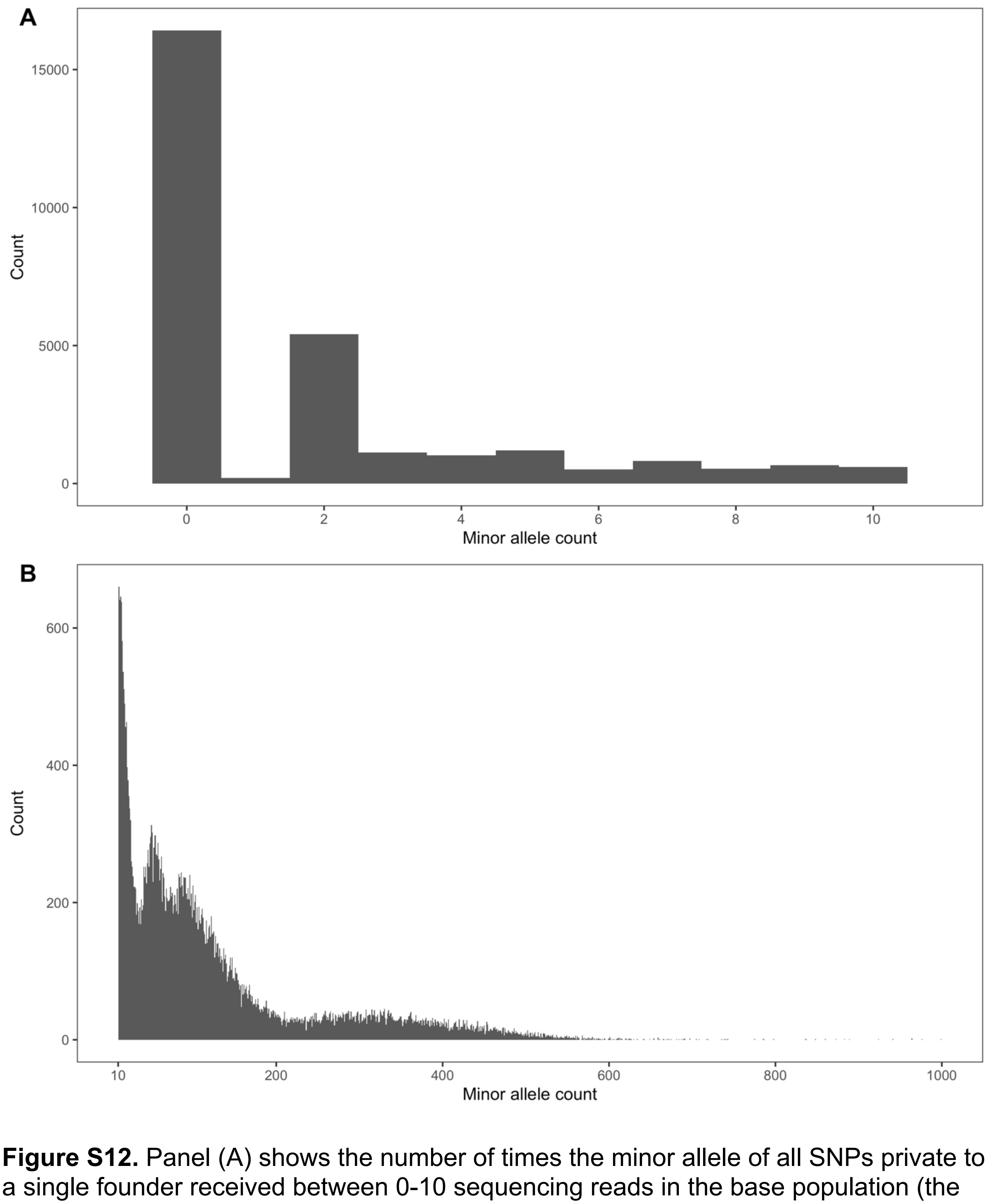

Figure S12. Panel (A) shows the number of times the minor allele of all SNPs private to a single founder received between 0-10 sequencing reads in the base population (the Minor allele count), which was sequenced to a depth of 2226X. Panel (B) is similar but the range is from 10-1000 sequencing reads in the base population.

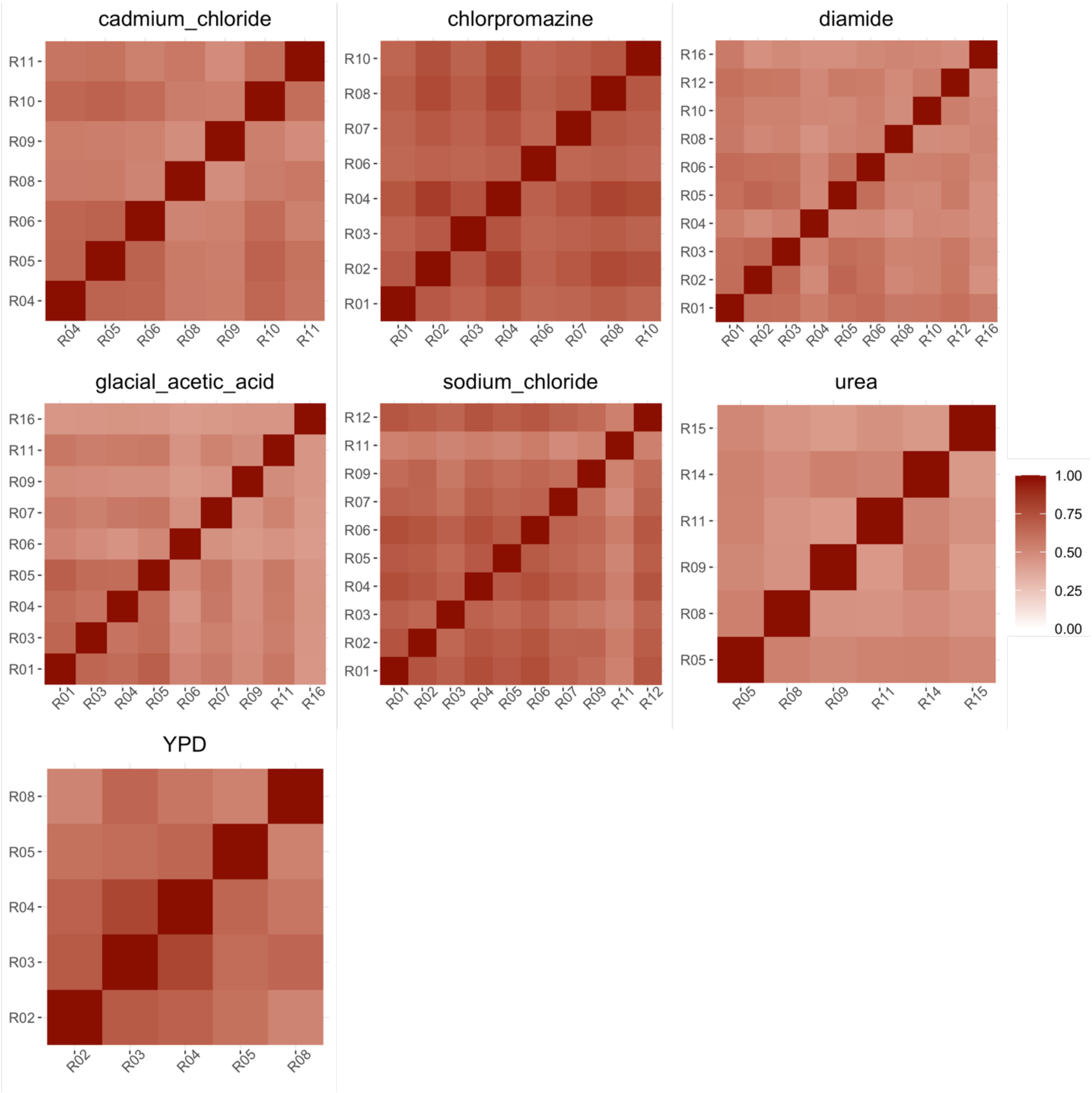

Figure S13. Heat maps of the Spearman correlations within chemical treatments.

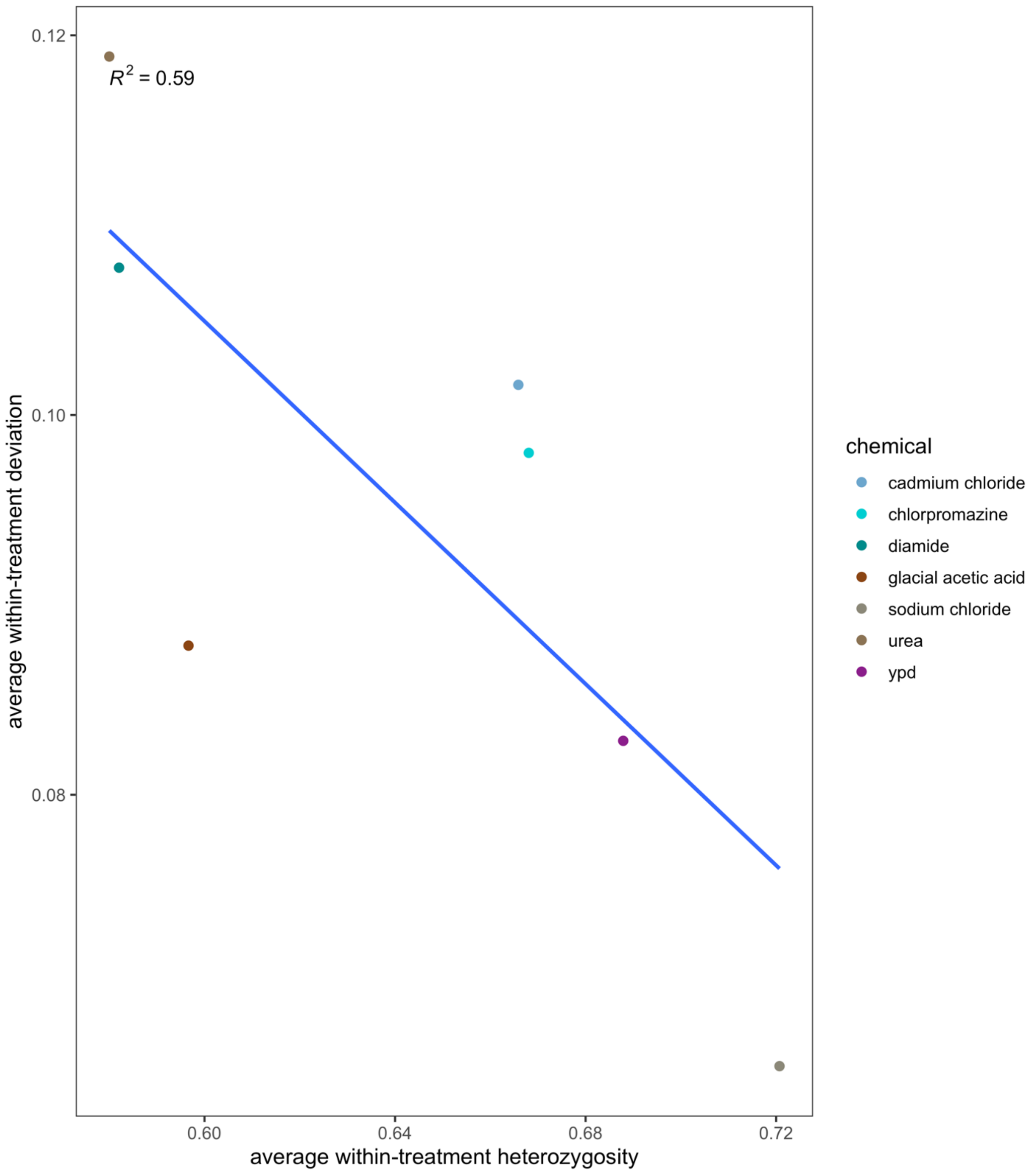

Figure S14. The correlation between the average heterozygosity and the mean within treatment absolute deviation of the frequency of the MIH of a single replicate from the average of the remaining replicates.

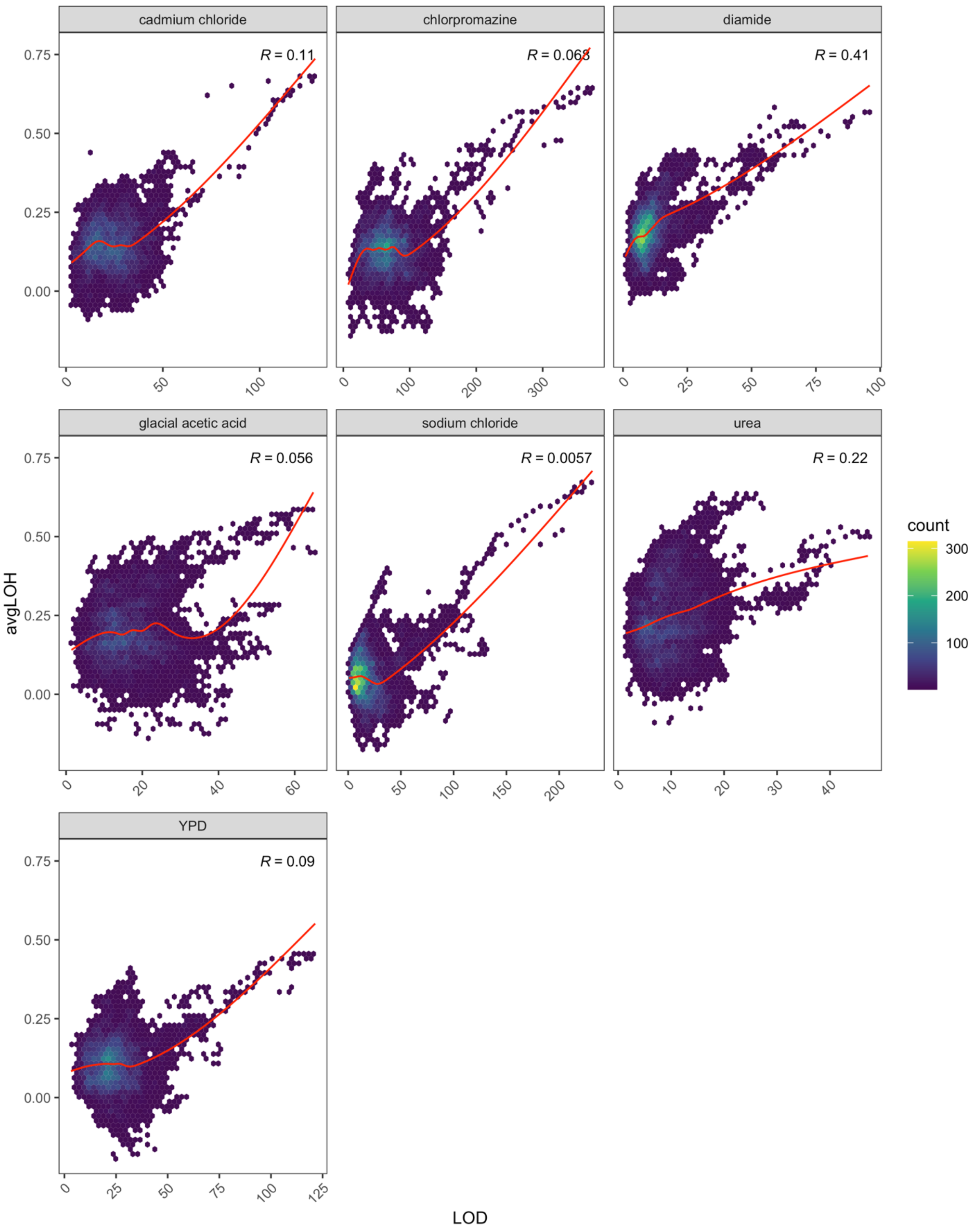

Figure S15. The correlation between LOD score and the average loss-of-heterozygosity per site genome-wide.

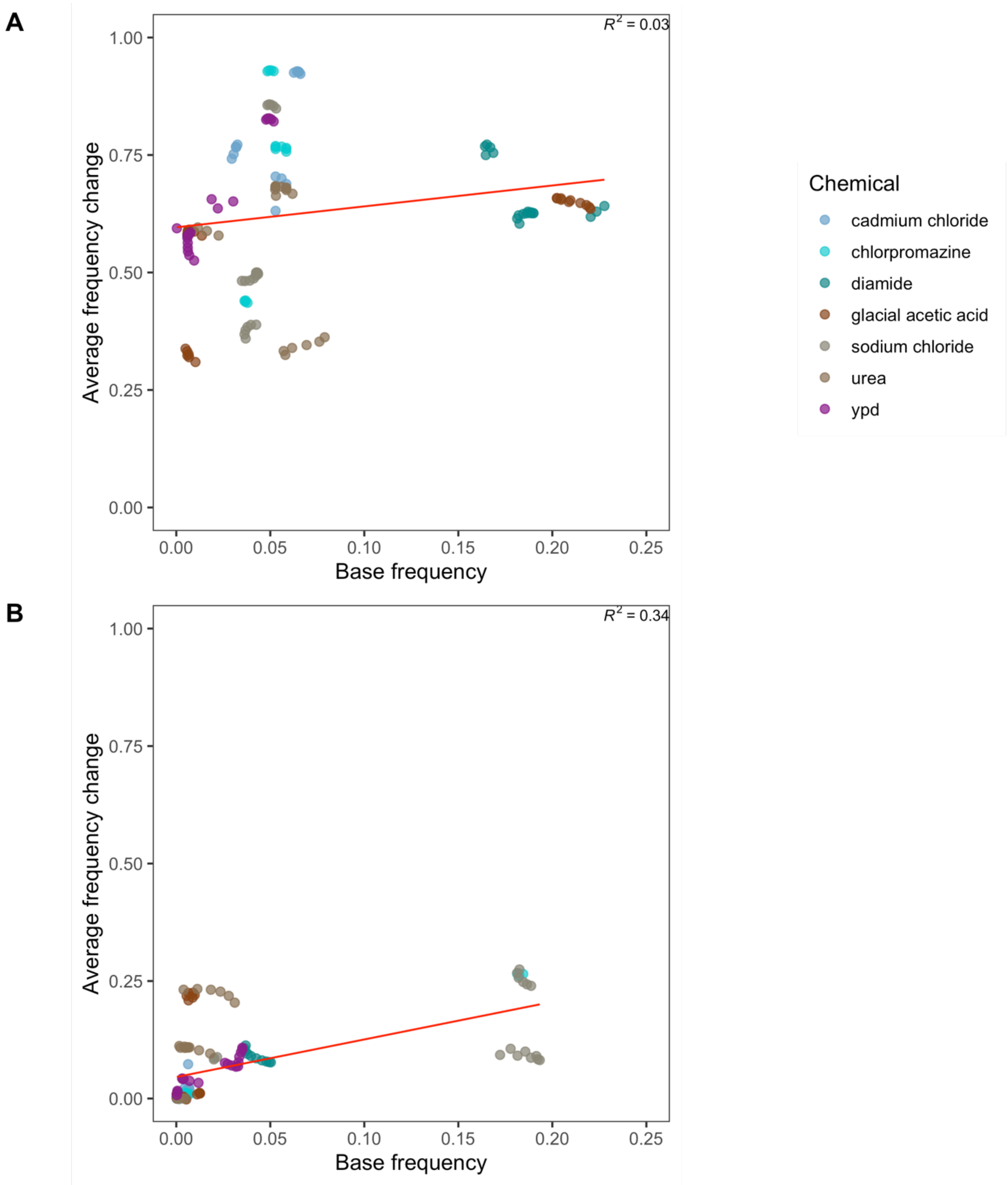

Figure S16. Panel (A) shows the average frequency change of the most increased haplotype while panel (B) shows the next most increased haplotype at each of the 21 major peaks detected regressed onto the initial frequency of the respective haplotypes in the base population.

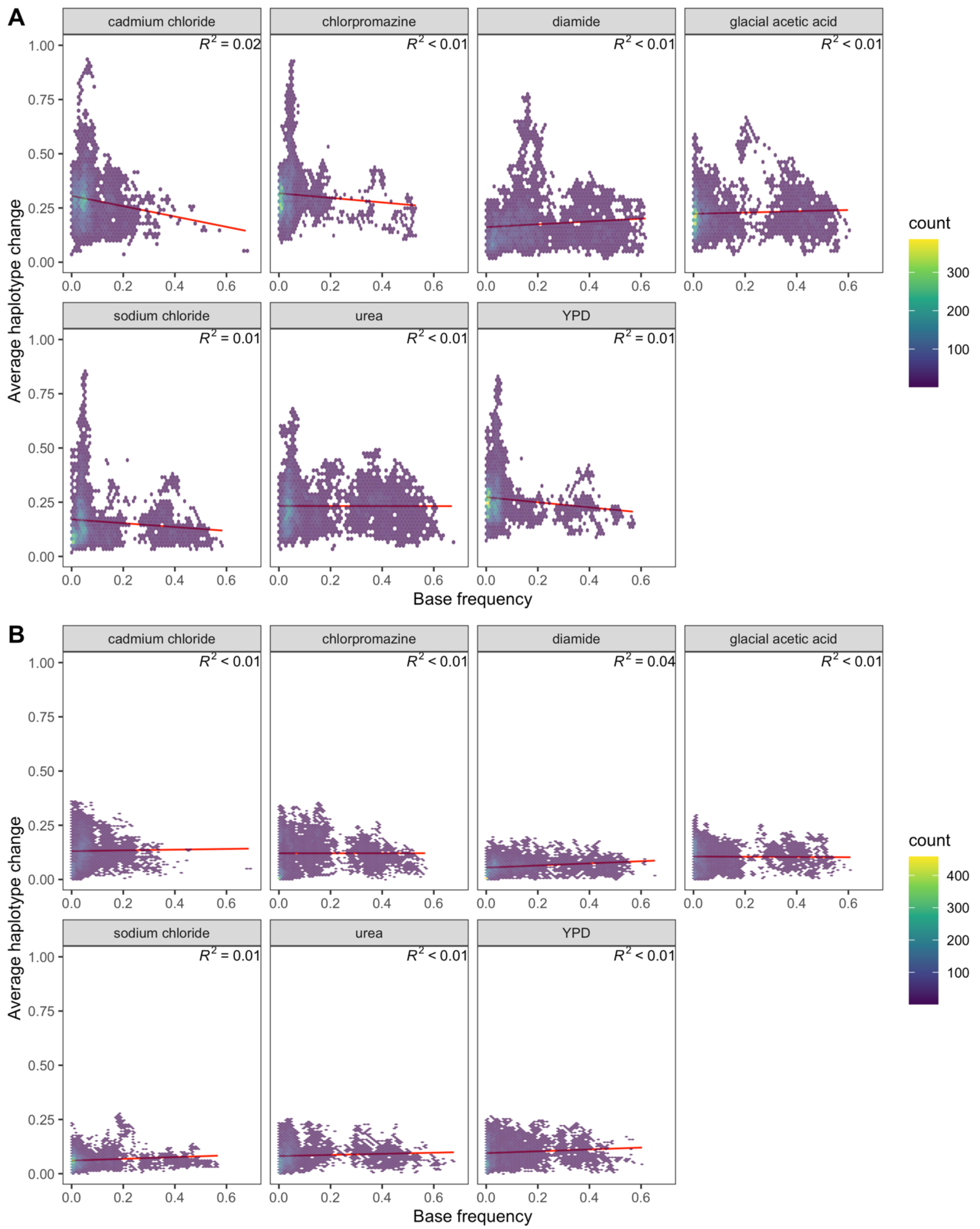

Figure S17. Panel (A) shows the average frequency change of the most increased haplotype while panel (B) shows the next most increased haplotype per-site genome-wide regressed onto the initial frequency of the respective haplotypes in the base population.

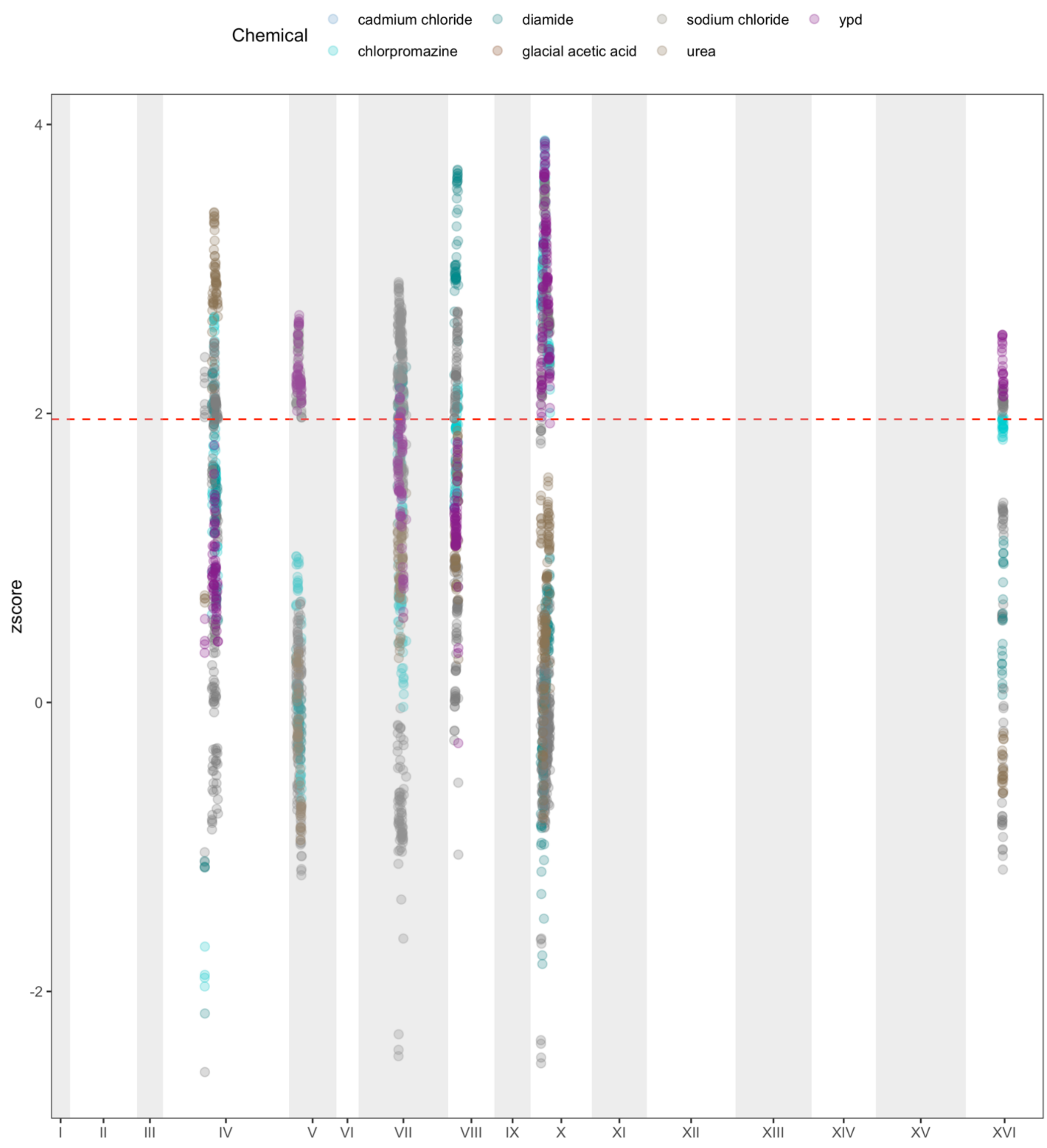

Figure S18. Only regions in which two or more chemicals had a z-score >= 1.96 were plotted to find regions that may represent instances of pleiotropy.

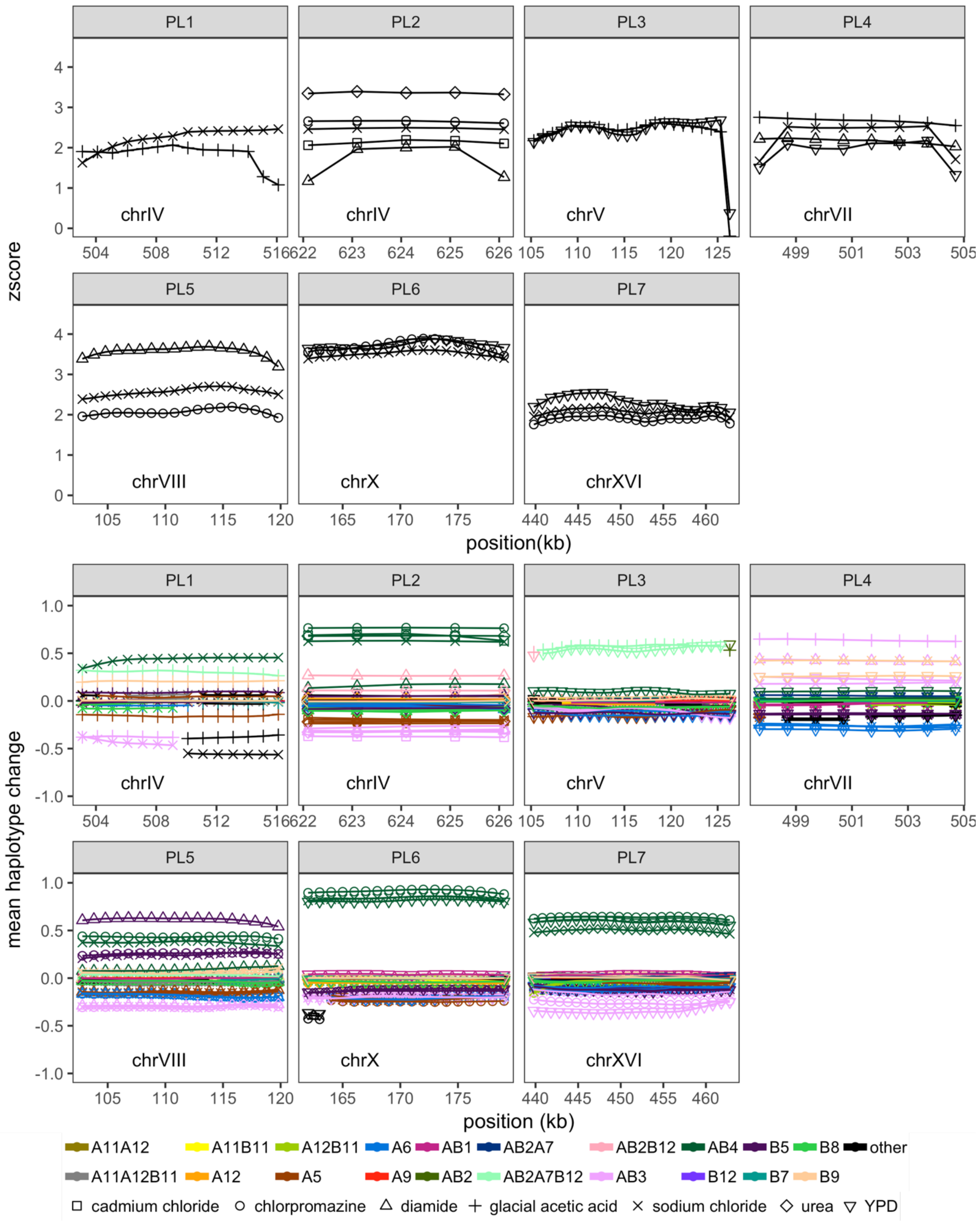

Figure S19. Potentially pleiotropic regions (labeled by peak ID) are shown. The z-score of transformed LOD scores is shown in panel (A), while panel (B) shows the mean haplotype frequency change across each region.

Supplemental Results

Candidate genes and candidate causative variants for the 21 leading factors

To identify major effect candidate genes and/or potentially causative variants, we examined the top three peaks from each condition, and for each peak define a 2.5-LOD support interval. Across all ten treatments, the average size of the 2.5-LOD support interval was 6kb and contained on average 5 genes (Table S6).

Under eight of the twenty-one total peaks examined (CD1, CD2, CD3, DI1, DI2, DI3, NC1, and NC3), we identified a single gene (*PCA1, VTC5, AFT2, QCR10, YIRCdelta6, SIL1, ENA1,* and *TRK1*, respectively; bolded in Table S6) that has previously been either shown *a priori* to respond to the same chemical stressor as used in this study or to be the only annotated feature under the peak. Based on an *a posteriori* examination of the function of each gene under the remaining 13 peaks, 4 (CP1, CP2, NC2, and UR2) had a single candidate gene (*VTC5, GPA1, GPA1*, and *ILT1,* respectively) with functions closely related to the chemical challenge (underlined in Table S6). The remaining nine peaks did not have strong *a priori* or *a posteriori* candidates. Detailed below is evidence that makes the genes listed above strong candidates for subsequent follow-up studies as well as potentially causal variants that could be functionally validated in future work across all 21 peaks.

Cadmium chloride tolerance has previously been shown to be mediated in large part by *PCA1*, a cadmium transporter that has been detected in the same chemical treatment in a previous X-QTL study (Ehrenreich et al. 2010) and that is known to confer tolerance to

cadmium chloride treatment (Shiraishi et al. 2000). A moderately sized in-frame insertion occurs in *PCA1* in four founder strains, including the Most Increased Haplotype (MIH), B8, which has a 36bp insertion towards the beginning of the protein, as does AB4 and B9, while A5 has a 72bp insertion. B8 is further distinguished from AB4 and B9 by 6 nonsynonymous SNPs, 4 of which are predicted to change the secondary structure of the protein. Previous work has shown that deletion of the candidate gene *YDR089W* (*VTC5*) leads to increased cadmium chloride resistance through an unknown mechanism (Ruotolo et al. 2008). *VTC5* may influence heavy metal tolerance through its' regulation of the production of polyphosphate molecules (Desfougères et al. 2016), which have been found to be involved in resistance to manganese and cadmium and are present in the vacuole of *S. cerevisiae (Andreeva et al. 2013; Trilisenko et al. 2017)* and can increase tolerance to cadmium in bacteria (Keasling and Hupf 1996). AB4, the MIH for this region, has a single nonsynonymous SNP in a highly conserved region predicted to change the secondary structure of the protein. This SNP is shared by founder A7- however, A7 is at the lower limit of our ability to detect haplotypes and may not be present at this locus. Deletion of *AFT2,* an iron-regulated transcription factor, has been shown to sensitize yeast to cadmium exposure, possibly due to cadmium interfering with iron homeostasis (Ruotolo et al. 2008; Thorsen et al. 2009). A variant private to the MIH establishes a *YAP1* binding site upstream of the TSS in *AFT2* (Figure 4). *YAP1* itself has previously been shown to induce the expression of several genes involved in tolerance to cadmium chloride (Wemmie et al. 1994).

Chlorpromazine is known to have a multitude of effects in budding yeast, including inducing oxidative stress, altering membrane integrity, decreasing cellular lipid levels (Muhieddine et al. 2019), and inhibiting intracellular protein trafficking (Nierras and Warner 1999). *VTC5* (Figure S8A) is also a strong candidate gene for this drug, as a reduced intracellular pH has been shown to interfere with chlorpromazine's toxicity (Ahyayauch et al. 2003), and *VTC5* has a role in maintaining the pH of the vacuole (Desfougères et al. 2016). The MIH for this region is the same as for the same region detected with cadmium chloride treatment. Another strong candidate is *GPA1*, is a gene with a known role in the response to mating pheromone (Miyajima et al. 1987) as well as involvement in the MAPK pathway (Metodiev et al. 2002), which interacts with a Phosphatidylinositol 3-Kinase to increase cellular levels of phosphatidylinositol 3-Phosphate. The MIH, AB4, has a coding SNP at a highly conserved location predicted to change the secondary structure of the protein. This SNP is shared with founder B5-both founder haplotypes increase significantly in frequency at this locus, with AB4 increasing ~12.5-fold and B5 increasing by ~2.5-fold as compared to initial frequencies in the base population.

For diamide tolerance, deletion of the candidate gene *SIL1* (Figure S8B) leads to increased resistance (Siegenthaler et al. 2017). *SIL1* has been shown to affect protein folding in the ER by reducing a known *HSP70* chaperone, *KAR2 (Wang et al. 2014)*. The MIH, B5, has two nonsynonymous SNPs, one of which is predicted to change the secondary structure of the protein and is in a highly conserved region. Deletion of *QCR10,* a subunit of the mitochondrial respiratory chain, has also been shown to confer

sensitivity to diamide treatment (Thorpe et al. 2004). B5, the MIH, has a single private SNP upstream of the TSS that may establish a *CST6* binding site which the other founders lack. *CST6* has been shown to be involved in oxidative stress tolerance (Liu et al. 2016). The last candidate gene, *YIRCdelta6*, is a Ty1 retrotransposon that is present in all founders; however, the MIH, A5, has a SNP which deletes a downstream binding site for Stb5p. Deletion of this gene has previously been shown to specifically decrease levels of Ty1 transposition (Griffith et al. 2003).

One of the peaks detected for glacial acetic acid tolerance includes *URA3*, a gene that was knocked out in all founder strains to enable subsequent genetic manipulations. The deletion inserted a kanamycin resistance cassette at this locus. As kanamycin was used at high concentrations to prevent bacterial contamination in all cultures, we speculate that a *cis* factor or a mutation introduced during the deletion resulted in higher levels of kanamycin resistance expression in one of founders AB2, A7, or B12 (these 3 founders are indistinguishable in this region) and perhaps higher growth rates. Alternatively, the MIH at this locus has a binding site for Msn2p just upstream of the TSS of *GEA2*- this site is missing in the remaining founders. Msn2p is a transcriptional activator known to be involved in the response to acid stress (Causton et al. 2001; Mira et al. 2010). Only a single gene under GA2, *RPN14*, has a private coding SNP- this SNP is predicted to change the secondary structure of the protein. *RPN14* is a protein folding chaperone that helps to assemble the 19S regulatory particle of the proteasome (Roelofs et al. 2009; Saeki et al. 2009).

For salt tolerance, Ena1p is a well-known P-ATPase sodium pump involved in sodium efflux (Haro et al. 1991)(Figure S10A). The *ENA* gene region contains three genes *ENA1*, *ENA2*, and *ENA5*, with our base population segregating a complex structural variant. In 10 of 18 of the founders the *ENA* locus has been inverted with only the *ENA1* gene present, while the other founders have all three known *ENA* genes present in the reference orientation. Counter-intuitively, one of the founders with only *ENA1* (AB4) is the most increased in frequency for this candidate region, so the lack of *ENA2* and *ENA5* alone is not sufficient to explain adaptation. A potential explanation lies in the fact that the AB4 founder additionally lacks a predicted binding site for the transcriptional repressor (Mig1p), which is known to repress genes in the presence of glucose (Nehlin and Ronne 1990)- only one other founder (A5) lacks this binding site. If this hypothesis is correct the allele responding to selection is due to an epistatic interaction between two mutations. Finally, there is a nonsynonymous SNP in *ENA1* that distinguishes founders AB4 and A5, but it is unclear if this SNP in concert with the loss of a Mig1p binding site can explain the haplotype change. Another candidate gene for sodium chloride tolerance is *TRK1*, a high-affinity potassium transporter (Gaber et al. 1988), deletion of which leads to increased sensitivity to sodium ions, potentially due to the ability of Trk1p to discriminate between potassium and sodium ions (Haro et al. 1993). The MIH, AB4, has two insertions that lead to the addition of an aspartic acid and two more lysine residues in the CDS which the rest of the founders lack. AB4 also has 3 nonsynonymous SNPs, all predicted to change the secondary structure of the protein, and 2 synonymous SNPs, one of which lies in a highly conserved region. *GPA1* has

also been predicted to have a role in the cellular response to high osmolarity (Hohmann 2002) and shares the same MIH as that detected in chlorpromazine treatment.

Nitrogen catabolite repression (NCR) occurs when a preferred nitrogen source is present in the media (such as ammonium and asparagine), leading to the repression of genes involved in the utilization of non-preferred nitrogen sources (such as proline and urea). In the presence of a preferred nitrogen source, the transcription factors Gat1p and Gln3p are sequestered in the cytoplasm by Ure2p. Once a preferred nitrogen source has become limiting, Gln3p and Gat1p are dephosphorylated and localize to the nucleus, where they activate transcription of genes needed to use non-preferred nitrogen sources (reviewed in (Hofman-Bang 1999)). One of the urea resistance peaks (UR2) contain a gene (*ILT1*) shown to have transcriptional profiles similar to other known NCR genes when exposed to different nitrogen sources (Boer et al. 2007), making them good candidates for this trait. The MIH at *ILT1* has an Hsf1p binding site (Hsf1p responds to highly diverse stressors) and is missing a Gat1p binding site the other founders all have, which may be beneficial if Ilt1p, an integral membrane protein, is involved in the uptake of urea.

For growth in rich media, there are no clear-cut candidates within YP1, although there are abundant potentially functional private variants in the MIH, including 5 nonsynonymous SNPs (all of which are predicted to change the secondary structure of their cognate protein) and 6 synonymous SNPs. YP2 contains the previously described *TRK1* gene as well as *URA2*, with the same MIH as detected in sodium chloride treatment. Ura2p is a bifunctional protein that catalyzes the first two steps in the *de*

novo biosynthesis of pyrimidines (Lue and Kaplan 1969; Souciet et al. 1982). The MIH, AB4 has two nonsynonymous SNPs (one of which is predicted to change the secondary structure of the protein) and three silent SNPs- all five of these coding SNPs are in highly conserved regions. The MIH, AB4, for YP3 has 3 private SNPs present just downstream of *YPR015C*, a gene previously shown to be involved in anaerobic growth (Samanfar et al. 2013).

Candidate genes and candidate causative variants for the regions exhibiting pleiotropy

Despite pleiotropy not being universal there were several regions of the genome that show strong across treatment correlations in LOD scores for subsets of chemicals (Figure 9). To identify the specific genomic regions exhibiting pleiotropy we calculated z-scores from the log-transformed LOD scores and regions having z-scores >=1.96 for at least two drugs were plotted (Figure S19). There are seven such strong regions genome-wide (Figure S19), including: two regions on chromosome IV (one shared between glacial acetic acid and sodium chloride, the other shared between urea, chlorpromazine, sodium chloride, diamide, and cadmium chloride), a region on chromosome V (shared between glacial acetic acid and YPD), a region on chromosome VII (shared between YPD, diamide, glacial acetic acid, and sodium chloride), a region on chromosome VIII (shared between chlorpromazine, sodium chloride, and diamide), a region on chromosome X (shared between chlorpromazine, sodium chloride, and YPD; see Figure 9), and a region on chromosome XVI (shared between YPD, sodium chloride, and chlorpromazine). For all seven potentially pleiotropic regions, Table S7 shows genes contained within the overlapping peaks shown in Figure S20.

For PL1, the MIH is different for glacial acetic acid and sodium chloride, which hints that different genes within this region may be causal. *MRH1* is a good candidate for glacial acetic acid tolerance as deletion has been shown to confer sensitivity to this stressor (Takabatake et al. 2015)- there is a single synonymous SNP private to the MIH at a highly conserved region. Alternatively, *LYS14*, which regulates lysine biosynthesis, is a good candidate for sodium chloride tolerance as the MIH has 3 nonsynonymous SNPs, 2 of which are predicted to change the secondary structure of the protein and 2 of which are highly conserved, 3 synonymous SNPs, 2 of which are also highly conserved, and a *MSN2* binding site the other founders lack. Msn2p is a transcriptional activator known to be involved in the response to multiple different stressors (Gasch et al. 2000). For PL2, diamide has a different MIH than the other four chemicals with peaks detected in this region (all of which have AB4 as the MIH). At this locus, the most likely candidate is *VTC5*, which, as described above, is a candidate gene for both cadmium chloride and chlorpromazine tolerance. For PL3, the most likely candidate is *PMP2*, a gene that previously has been shown to be involved in acetic acid tolerance (Mira et al. 2010) and that regulates the activity of Pma1p (Navarre and Goffeau 1998), which is an H(+)-ATPase at the plasmid membrane. At PL4, the MIH for diamide and glacial acetic acid is AB3, while the MIH for sodium chloride and YPD is B9. Five genes are contained within the detected interval, including *SWC4*, a component of a histone acetyltransferase complex, and *CUL3*, an E3 ubiquitin ligase involved in RNA polymerase II degradation. In founder B9, there is a nonsynonymous SNP at a highly conserved sequence in both *SWC4* and *CUL3*, while founder AB3 has a single SNP in the promoters of both *SWC4* and *CUL3*, with a single synonymous SNP in *CUL3* at a

highly conserved sequence. The portions of *EFM5* and *TFG2* contained within this interval do not have private SNPs unique to either founder.

For the three chemicals detected in PL5, the MIH for diamide is B5, while the MIH for chlorpromazine and sodium chloride is AB4. Good candidate genes for these drugs include *QCR10* for diamide and *GPA1* for chlorpromazine and sodium chloride, as described above. For PL6, *TRK1, URA2,* and *PBS2* are all good candidates- all harbor multiple SNPs private to AB4. As described above, Ura2p is part of the *de novo* pyrimidine biosynthesis pathway, while Trk1p is a potassium transporter. It is possible that the *de novo* synthesis of pyrimidine provides a benefit under these specific conditions, although, as *TRK1* has previously been shown to affect sensitivity to sodium ions, this may be the better candidate. Pbs2p mutants have previously been shown to have a defective cell wall and increased susceptibility to hyperosmotic stress (Gopalbhai et al. 2003), which makes it a good candidate for both chlorpromazine and sodium chloride tolerance, respectively. For the final pleiotropic peak, PL7, there are three candidate genes, including *GRX5, PDR12,* and *SUR1*. *GRX5* has previously been shown to be involved in the oxidative stress and osmotic stress response (Chabrier-Roselló et al. 2010; Chakrabortee et al. 2016), making it a good candidate for all three drugs (chlorpromazine, sodium chloride, and YPD). *PDR12* is a multidrug transporter, while *SUR1* has also been shown to be involved in the oxidative stress response (Helsen et al. 2020) and is required for the biosynthesis of sphingolipids (reviewed in (Dickson and Lester 1999)). The MIH at this region is AB4, which, at *GRX5,* has only a single SNP in the promoter region, while at *PDR12* there are two synonymous SNPs,

both in highly conserved sequences, while at *SUR1* there is a single synonymous SNP at a highly conserved sequence.

Supplemental Notes

Note S1. We discovered that there were two classes of potential ‘cheaters’ that had emerged during evolution. One, the aneuploid haploids, turned out to be haploid at all but the mating type locus. Upon closer examination of sequencing coverage, we found evidence that either a nondisjunction event occurred during meiosis causing chromosomes harboring both mating type loci (a/@) to be retained in an otherwise haploid background or a recombination event occurred in which the entirety of one mating type locus inserted into the opposite mating type locus. Either situation allowed these cells to escape diploid selection, as the markers used to select diploids were tightly linked to the mating type locus. Further, these haploid cheaters seemed as resistant to lysis as actual spores; we later found that only treatment with ether (which cannot be done safely in high throughput) can kill these cells without killing off all the spores as well (treatment with heat shock did not seem to work either, which would have been more amenable to high throughput). The second type of cheater looks like a single heterozygous diploid clone had come to dominate the population. However, we found that these populations do sporulate (unpublished results) and observed haploid spores (using fluorescence microscopy) that predominate after sporulation. Thus, it may be that these populations in fact represent instances in which one or two beneficial haplotypes are fixed across the genome. This is possible due to the highly polygenic nature of adaptation that we observed.

Supplementary Materials and Methods

Strains and media: The base population used in this study is a multi-parent population (MPP) derived from a full diallel cross of 18 highly characterized natural haploid founding strains (Cubillos et al. 2009) intercrossed for 12 generations to break up haplotype blocks. This population, 18F12v2, and the 18 founding strains are described in detail elsewhere (Linder et al. 2020). Non-selective propagation of yeast was carried out using rich YPD media (1% yeast extract, 2% peptone, and 2% dextrose (Fisher, Waltham, MA)) with 2% agar added for solid media. In some cases the YPD was supplemented with 100mg/mL ampicillin (YPDamp) or 200mg/mL of kanamycin (YPDk) to prevent bacterial contamination. During long-term evolution the YPDk media was spiked with 2 different doses of each chemical (a day 1 of the entire experiment dose and a day 2 onwards dose; see Table S1). Sporulation media consisted of 1% potassium acetate and a 1X dilution of a 10X amino acid stock (composed of 3.7g of CSM -lysine (Sunrise Scientific, Knoxville, TN) supplemented w/10mL of 10mg/mL lysine in 1L total volume), pH adjusted to 7 ('PA7'). Mating media consisted of YPDamp. Spore isolation solution ('SIS') consisted of 25U zymolyase 100T (Amsbio, Abingdon, United Kingdom) + 10mM DTT (dithiothreitol; Thermo, Waltham, MA) + 50mM EDTA (ethylenediaminetetraacetic, Fisher) + 100mM Tris-HCl, pH 7.2 up to 500uL. Spore dispersal solution ('SDS') consisted of 20U zymolyase 100T + 800ug lysozyme (Sigma, St. Louis, MO) + 1% Triton X-100 + 2% dextrose + 100mM PBS, pH 7.2 up to 400uL. Double selection media ('DSM') consisted of 200ug/mL nourseothricin sulfate ('clonNAT') + 600ug/mL hygromycin b + 100ug/mL ampicillin in YPD. Concentrations of cloNAT and hygromycin b are twice as high as normal as DSM was added in equal volume to the media used for mating (i.e. diluted 2x).

Long-term evolution of diploid budding yeast: The long-term evolution experiment was initiated from 6mL of 18F12v2 (hereafter the base population) thawed at RT, spun down, and resuspended in 20mL of YPDamp, split into two 50mL erlenmeyer flasks (covered) and incubated at 30°C at 275 RPM for 3h to ensure cells had entered exponential growth. Cells were harvested in YPD, counted using a haemocytometer, and 140uL of culture containing ~1.4 million cells was transferred into shallow 24-well plates (Phenix, Candler, NC; TCG-662160) containing 1.4mL YPDk supplemented with

15 different chemicals (as well as YPDk-only controls). These 24-well plates allow for a 1.5mL working volume with shaking with minimal cross-contamination. In total, 16 replicate populations were spiked into each of the 15 chemicals, along with a total of 10 YPDk-only replicate control populations. Wells containing only YPDk (no cells) were scattered across plates to monitor for signs of cross-contamination (6 negative control wells total). The total experiment thus consisted of eleven 24 well plates.

For transferring cultures to new media, the collection of 16 different YPDk + chemical plates were made in bulk and stored at -80°C for up to 6 months to minimize batch effects. Plates were thawed at 30°C for ~2h just prior to being used. Tables 1 and S1 list the chemicals used and their known effects in budding yeast, as well as the doses used in this experiment. Chemicals used included: chlorpromazine, cisplatin, fluconazole (all from Cayman, Ann Arbor, MI), diamide, sodium sulfite, dmso, urea (all from Fisher), ethanol, sodium chloride, nicotine (all from VWR, Radnor, PA), cadmium chloride, nicotinamide (both from Sigma), tunicamycin (Enzo Life Sciences, Farmingdale, NY), glacial acetic acid (J.T. Baker, Phillipsburg, NJ), and caffeine (ICN Biomedicals, Costa Mesa, CA).

The schematic of the long-term evolution regimen is shown in Figure 1. On the first Tuesday of the entire experiment only, a lower dose of most chemicals was used to acclimate cells to the chemical challenge (to allow cells to physiologically adapt). For the remainder of the experiment, an increased dose was used to maintain a high degree of selective pressure. On Tuesdays, Wednesdays, and Thursdays, 10x serial dilutions of cells were transferred to newly thawed chemical plates using a custom-built liquid-handling robot using 1000uL Tecan Freedom EVO Tips (Phenix, TRXF-HTR1000CS, filtered). The robot is contained in an acrylic cover with a large HEPA-filtered blower placed on top to maintain laminar flow and ensure that cultures could be transferred aseptically. Plates were covered in adhesive membranes (VWR, 60941-086) and transferred to a plate-holding device that was placed into a shaker-incubator. Cultures were incubated at 30°C for 24h at 175 RPM. During plate transfers, it is of note that the adhesive membranes were pierced directly by the robot without need for removing the membranes beforehand to minimize the chances of cross-contamination. In addition, every Thursday, the OD630 of all cultures were read in a shallow, clear 96-well plate

(Phenix, MPA3370), and glycerol stocks (an equal volume of 40% glycerol added to the culture) were created for three copies of all cultures and stored at -80°C to maintain a fossil record of evolution.

On Fridays, cells were transferred to deep 24-well plates (Phenix, MP-2061), spun down, washed once with 1mL of sterile milli Q water (smq H2O), then resuspended in 1mL of sporulation media. Cultures were incubated for 3d (over the weekend) at 30°C at 200 RPM. All spin downs of plates with adhesive membranes were carried out at 1500 RPM, as faster speeds caused membranes to tear. On Mondays, a random subset of cultures were examined to ensure at least 50% of the culture had sporulated, after which the deep 24-well plates were spun down and pellets resuspended in 500uL of SIS. Cultures were transferred to a deep 96-well plate (Phenix, M-0564), sealed with a plate seal, vortexed on a plate vortexer for 5m at 1200 RPM, then incubated at 30°C for 1h at 200 RPM to spheroplast cells. Cultures were then spun down and resuspended in 1% Tween 20 to lyse unsporulated cells. Cultures were again spun down, resuspended in 400uL of SDS, and transferred to another deep 96-well plate loaded with 300uL of 400um silica beads. Plates were sealed (Phenix, SMX-DW96), tetrads were disrupted and sister spores dispersed by shaking in the GenoGrinder 2000 at a setting of 1500 strokes per minute for 20m. To transfer disrupted spores to a new plate we used a chimney PCR plate (LightLabs, Aurora, CO; A-3004-C) with holes burned into the base using a soldering iron. The top of the chimney plate was forced into the deep 96-well plate and the base inserted into a second deep 96-well plate (Phenix, MDZ96-22SV) and spun until the centrifuge reached 1500 RPM**.** This process was then repeated again to minimize the carry-over of beads. Cells were pelleted, washed in YPDamp, resuspended in 250uL of YPDamp, and allowed to mate for 3.5h at 30°C at 25 RPM. 250uL of DSM was then added to all cultures to select for successfully mated diploids and cells were incubated overnight at 175 RPM at 30°C. On Tuesday, 140uL of cells were transferred into chemical plates to begin the cycle again. In total, evolution was carried out for 12 weeks, with an estimated 18 mitotic and 1 meiotic generation occurring each week for a total of 216 mitotic and 12 meiotic generations.

Revival of frozen stock to resume evolution: Periodically, evolution would need to be 'rebooted' from a previously archived week. This occurred due to technical issues with our instruments or other catastrophic cross-contamination events. To reboot evolution, glycerol stock plates were taken from the -80°C freezer on a Monday, spun down briefly (up to 1500 RPM), placed on the robot deck, and allowed to thaw completely at RT. Cultures were then transferred to deep 96-well plates, spun down, washed with 330uL of smq H2O, spun down again, then resuspended in 500uL of YPDamp and incubated O/N at 175 RPM at 30°C to allow the populations to recover. Cultures were then serial transferred into their respective chemical stressor in shallow 24-well plates for 3 days before sporulating on Friday. As the glycerol stocks were made on a Thursday, a reboot skips a meiosis cycle. *This strategy was necessitated as we observed that the rate of sporulation increased the longer we allowed cultures to divide mitotically with serial dilutions after being revived from a glycerol stock*. We have not seen this claim elsewhere in the literature, but it is a very replicable result.

Whole genome sequencing of the evolved populations: Archived week 12 evolved populations were thawed and incubated in deep 96-well plates in 960uL of fresh YPDamp O/N at 30°C at 175 RPM. Cells were then harvested, washed once with buffer TE, and genomic DNA extracted using a modified 96-well DNA extraction protocol. Spheroplasting was carried out at 37°C for 30m with zymolyase-containing buffer, followed by incubation in lysis buffer and proteinase K at 50°C for 60m (68°C for 10m at the end to kill the proteinase activity). Cell lysates were then incubated in 5M potassium acetate at 4°C for 30m. An additional 100uL of sigma water was added and samples were spun down and the supernatant transferred to a clean 96-well PCR plate. An additional centrifugation step was carried out, followed by transferring the DNA-containing supernatant to an equal volume of RT 100% isopropanol. Samples were then spun down, washed with RT 80% ethanol, spun down again, air dried, and finally purified DNA was resuspended in 50uL of warm buffer EB. DNA was quantified using the Qubit 1.0, followed by preparing the samples for Illumina sequencing using the Nextera Flex kit with modifications. Reactions were carried out at 1/5th of the normal volume as detailed in our previous study (Linder et al. 2020). Libraries were sequenced

on the HiSeq4000 with PE100 reads. Coverage per sample ranged from 5x to 867x, with a mean coverage of 67x (Figure S2).

Isolation of haploid clones: Three archived week 11 evolved populations (cadmium chloride R04, diamide R01, and sodium chloride R02) as well as the base population were inoculated directly into 2mL of YPDamp in culture tubes and incubated O/N at 175 RPM. Cells were then spun down at 2500 RPM for 5m, resuspended in 1mL of smqH_2O, transferred to 1.5mL eppendorf tubes, spun down at 7500 RPM for 5m, resuspended in 1mL of PA7, transferred to 24-well plates (Phenix, MP-2061) and incubated at 30°C at 200 RPM for 4d. 1mL of sporulated culture was transferred to separate eppendorf tubes, spun down at 7500 RPM for 5m, resuspended in 500uL of SIS, vortexed at full speed for 1m and transferred to a 96-well plate (Phenix, M-0564) to incubate for 1h at 30°C at 200 RPM to spheroplast cells. Cells were then transferred to eppendorf tubes, spun down at 7500 RPM for 5m, resuspended in 500uL of 1% Tween 20 (sterile), vortexed for 1m at full speed, spun down at 7500 RPM for 5m, and resuspended in 1mL of SDS. Processed spores were then transferred to eppendorf tubes with 500uL of sterile acid-washed 400um silica beads, bead milled until tetrad disruption for 10m total, then spun down at 2000xg for 2m to get rid of the foam. Supernatant was transferred to a separate tube, spun down at 10,000 RPM for 5m, and resuspended in 500uL of 0.1M PBS, pH7.2 + 0.015% IGEPAL. Samples were vortexed at high speed for 30s, then sonicated on ice for 20s at 30% amplitude using a tip sonicator (Sonics vibracell Ultrasonic processor) in order to completely disrupt tetrads separate sister spores. For the three week 11 populations, an additional sonication at 40% amplitude for 30s was carried out, while for the base population an additional sonication at 40% amplitude for 10s was carried out. Spores were examined under a microscope to confirm tetrad disruption. Serial dilutions of spores were then plated onto YPD + cloNAT plates to select for Mat *a* haploids and incubated at 30°C for 2d. Haploid clones were transferred to 700uL of YPDamp + cloNAT using sterile toothpicks in deep 96-well plates and incubated O/N at 175 RPM at 30°C (96 clones were picked from the base population, 48 clones from each of the week 11 populations). 200uL from all cultures were then transferred to shallow 96-well plates, with the remaining cultures covered with an adhesive membrane and stored at 4°C. Haploid clones were pinned

onto omnitrays supplemented with either mating-type tester 1 or mating-type tester 2 as described in (Linder et al. 2020) as well as a YPD-only control. Plates for incubated for 3d at 30°C and 50uL of confirmed Mat *a* haploids were transferred from the source plates stored at 4°C to deep 96-well plates with 950uL of YDPamp O/N at 30°C at 175 RPM (48 haploid clones were selected from the base population and 24 haploid clones were selected from the three week 11 populations). 140uL from all cultures were archived at -80°C, with the remaining cultures used as input for 96-well gDNA extractions and subsequent library prep as described above. Libraries were sequenced on the HiSeq4000 with PE100 reads.

Estimation of daily bottlenecks during a week of the long-term evolution regimen: To estimate bottlenecks and the number of divisions experienced by our populations during a typical week of evolution, a single sexual (see below) YPD population was sampled daily, starting Monday and ending Friday during week 11 of evolution (YPD replicate R10 - see Figure S1A/B). Sampling consisted of making serial dilutions in 0.1M PBS, pH 7.2, vortexing the dilutions vigorously, and plating out the dilution series onto YPDA plates shaken with beads to inhibit the clumping of cells. Plates were incubated for 2-3d at 30°C and imaged using a geldoc imager with the lids off. Colonies were automatically counted using ImageJ as detailed in (Stolze et al. 2019). The adjustable watershed add-on was used with tolerance set to 0.1. Particle size was set to 0.015" and circularity to 0.3. On Monday, samples were taken at three time-points (Figure S1C): a) post 3-days of sporulation while cells were still in sporulation media before any processing had occurred (with colonies measuring the number of viable tetrads), b) after spores had been dispersed and isolated, but just before mating (with colonies measuring the number of viable spores), and c) after 3.5-hours of mating before the addition of DSM or cell divisions would have occurred (measuring the number of viable diploid cells). Not surprisingly the maximal amount of bottle-necking experienced by the populations over the course of the week is observed in counts of diploid cells immediately after mating, as this time-point integrates over several previous steps in which spores are killed (due to breaking the ascus, vigorous dispersion, and/or vegetative cell killing steps) and/or are unable to mate or result in inviable diploids. This weekly nadir in census population size is important, as it is closely related to the

effective population size we were able to maintain during our experiment. We estimate that through much of the weekly cycle we maintain tens to hundreds of millions of cells, with a single weekly bottleneck in the high hundreds of thousands of diploid individuals.

Tracking cultures through time via weekly OD630 measurements: To keep track of which populations went extinct over the course of the experiment, OD630 measurements were taken every Thursday of all cultures in a clear, shallow 96-well plate. These weekly measurements consisted of using the liquid-handling robot to transfer 140uL from each shallow 24-well plate with cultures incubated between 23-25h (at the same time as serial transfers to fresh chemical plates were carried out). As a side-effect of measuring the total number of cells through a typical week of evolution (described above), we are able to estimate that cultures likely had not reached saturation at this point as saturated cultures of *S. cerevisiae* normally reach concentrations of over $5x10^8$ cells/mL (Chan et al. 2013). Thus, these OD630 measurements were taken while cultures were still growing and could be averaged across replicates and across time-points (to reduce the noisiness of the data) to correlate OD630 with the average number of well-behaved, outbred sexual populations that evolved from a chemical treatment.

Estimation of fitness gains after 12 weeks of evolution: To estimate the fitness gains of week 12 populations, a single evolved week 12 replicate population from each chemical was thawed, (along with the ancestral, week 0 population) transferred to a 96 deep-well plate and recovered O/N in 700uL of YPD supplemented with 100ug/mL ampicillin. To reacclimate populations to their respective chemicals of interest, populations were cultured in conditions nearly identical to the evolutionary regimen (the ancestral population was passaged only in YPD). Briefly, serial transfers of 140uL from each culture were made into a 24-well plate with the day 1 dose of their respective chemicals, followed by 24h of incubation at 30°C at 175 RPM, serial transfer of 140uL to the day 2+ dose of their respective chemicals, incubation at 30°C at 175 RPM for another day, followed by serial transfer of 50-200uL of culture to one (for our initial attempt) or eight (for our second attempt) fresh day 2+ chemical plates, which were incubated over a 24h period at 30°C at 175 RPM. Initially, we tried to measure the OD630 of a single plate over the 24h period with continuous shaking at 30°C in a plate

reader, but discovered that several populations tended to form large clumps on the bottom of the wells, causing sudden spikes and dips in OD630 readings and making it impossible to derive accurate growth curves from this data. Therefore, we repeated the experiment as described above, except that during the final day of incubation, plates were removed at several time-points over the 24h period, including: 2.5h, 8h, 9.5h, 12h, 14h, 16h, and 24h, to estimate the growth rate of each of the week 12 populations and the ancestral population in YPD. The entirety of each well was transferred to a 96 deep-well plate, spun down, supernatant was removed, and pellets were stored at 4°C overnight. The next day, all pellets were resuspended in 0.1M PBS, pH 7.2 and kept at 4°C. Samples were selected for further analysis from cultures that had not yet reached saturation but were likely no longer in lag phase, including the 2.5h time-point to be used as the baseline and one or two later time-points. Samples were sonicated briefly on ice to break up clumps in 0.1M PBS, pH 7.2 containing 0.015% NP40 (settings were 30% of maximum amplitude- about 4-6 volts, for 20s, continuous sonication using a Sonics Vibra cell sonicator). Serial dilutions of cells were plated onto YPDA, incubated for 3d at 30°C, then colonies were counted either manually when possible or using ImageJ with the same parameters as described above. To estimate fitness in the base population, we assumed cultures initially barely survive the daily 10X dilution (with the exception of the YPD-only condition) at the start of the experiment, as our initial doses of each chemical were chosen to be close to the maximum possible tolerated whilst avoiding extinction. We also observe early generation cultures decreasing in viable cell counts over time, but bouncing back by the end of the first week of evolution. Under this assumption, the population growth rate per hour for week zero, derived from the equation $N_t = N_0 e^{rt}$, is $r_0 = \frac{1}{24e}$. The growth rate at week 12 can similarly be estimated as $r_{12} = ((log_{10}N - log_{10}N_0)/e)/(t - t_0)$, which is the same as the slope from the regression of log10 counts on time in hours divided by *e*. From this, the change in population growth rate (as a proxy for fitness gain) is $r_{12}/r_0 = 24 *$slope estimate obtained from the 12-week evolved population. For estimating change in population growth rate for the YPD-only condition, the growth rate at week 0 (r_0) was estimated in the same way, using the same time-points, as for week 12.

Haplotype calling in Illumina sequenced evolved populations: A custom in-house haplotype caller was used to impute sliding window founder haplotype frequencies genome-wide, as described in (Linder et al. 2020). Briefly, we slide through the genome in 1kb steps, considering a 60kb window for each step. Due to the repetitive nature of the ends of chromosomes (Blackburn and Gall 1978), accurate haplotype frequency estimates are difficult to obtain, and so these regions are excluded. In total, we estimate haplotype frequencies at 11,604 loci spaced every 1kb throughout the genome. The 60kb window was decided by trial and error in our previous work and results in highly accurate haplotype frequency estimates with absolute per haplotype frequency errors of ~0.01 at the coverages employed in this study (Linder et al. 2020). The decision to use a 1kb step size balanced resolution with computational speed- at a 1kb step size the average absolute change in haplotype frequency between adjacent positions in founder AB3 is ~$4.5x10^{-7}$. Figure S3 shows the distribution of founder AB3 haplotype changes between all adjacent intervals estimated for the 55 populations classified as outbred sexuals and that clustered together from the seven chemicals used in downstream analyses (see below).

Only samples with genome-wide coverage greater than or equal to 5x were used for downstream analyses, while the 55 sexual populations that are the focus of this paper received a minimum of 38x genome-wide coverage. Due to the unique mosaic-like genome structure of many natural yeast isolates (Liti et al. 2009; Schacherer et al. 2009; Peter et al. 2018) there are many tens to hundreds of kilobase sized regions of the genome over which two or more of the 18 founder haplotypes cannot be distinguished from one another. In the case where two (or more) founder haplotypes cannot be distinguished from one another we are unable to estimate the frequency of those two founders for that region, but the sum of their frequencies is estimated as well as any other haplotype. In our previous work we thus estimated the frequency of f founders that cannot be distinguished for some window as the sum of their frequencies divided by f. When looking at regions important in adaptation, in some cases, this averaging approach is misleading, so here we took the different approach of defining new “synthetic founders”. For example, for a region at which founders A11 and A12 cannot be distinguished, we create a founder called ‘A11A12’ for that window and track

its frequency. This results in a more accurate picture of how regions are evolving, at the price of tracking a variable number of founder haplotypes for each region of the genome. We found that plotting the haplotype frequencies for the 23 most common haplotype combinations over all replicate populations and all chemical treatments (and an appropriate color palette) allowed us to visualize evolutionary change more easily. Over all windows, replicates, and treatments the 23 most common synthetic haplotype combinations account for an average of 94.3% of the observed haplotype frequencies.

Peak calling: We created a Chi-square test statistic to identify regions of the genome most changed due to selection within any given chemical treatment. At any given position we identified the *K* haplotypes (including possibly synthetic haplotypes) with a starting frequency of at least 1% in the base population. At that position for the *R* replicate evolved populations within the kth haplotype we calculate a normalized deviate from the expected change in allele frequency as

$$\Delta_k = \frac{\underline{\delta_k} - 0}{\sqrt{var(\underline{\delta_k})}},$$

where $\underline{\delta_k}$ is the average change in arcsin square root transformed haplotype frequency between the base and the R evolved replicate populations. Since $\Delta_k$is distributed as a unit normal, the sum of Δ_k^2 over the K haplotypes is distributed as a Chi-squared with *k* degrees of freedom. Although $var(\ \underline{\delta_k}\ )$ is unknown, we assume it is constant over haplotypes and estimate it as:

$$var(\ \underline{\delta_k}\ ) = [\underline{var(\delta_k)} + R * [\epsilon_B^2 + \epsilon_E^2]]/R,$$

where $\epsilon_{B/E}$ is the average error in the haplotype frequency estimates in the Base or Evolved populations, which we estimate as 0.004 or 0.01 respectively (Linder et al. 2020). That is, the average variance between experimental replicates plus the variance due to haplotype estimation error in the base and evolved populations. The different haplotype frequency errors are due to their being estimated from >2000x coverage sequencing in the base population as opposed to an average 53x in the evolved populations (with a range of 9x to 145x, Figure S2). However, it is important to note that the 55 evolved populations that are the focus of this paper were sequenced to an average of 112x (with a range of 38x to 867x). Further, we show in (Linder et al. 2020)

that the error in haplotype frequency estimation is not strongly affected by sequence coverage, consistent with other such reports in the literature (Tilk et al. 2019). For each of the *k* degree of freedom Chi-square tests suggested above, we obtain a p-value and represent the resulting support for change as a -log10(p)-value. Since much of the genome for all chemicals is above the nominal significance threshold suggested by the Chi-square test, we attempted to call local peaks using an algorithm that finds local minima and maxima in a vector with an adjustable threshold (Friedland, 2017).

Per-site heterozygosity deviation: The per-site haplotype heterozygosity was calculated at each of the 11,604 loci for the base and each evolved population as one minus the sum of the squared haplotype frequencies. We then calculated the average reduction in per-site heterozygosity by summing the differences in heterozygosity per-site between the base and each evolved population and dividing by the number of loci.

Classification of evolved replicate populations: We observed different types of evolved replicates after 12 weeks of evolution (Figure 2). Closer analysis led us to believe that some populations had been invaded by asexual 'cheaters' that rose to a high population frequency. We believe this was driven by individuals exploiting a strategy in which they did not sporulate and/or randomly mate, while being able to survive our stringent spore isolation protocol. The observed ~600-fold reduction in the number of mated spores by Monday evening relative to the number of diploids going into sporulation on Friday makes it clear there was a huge fitness cost to "playing by the rules". We observed two types of cheater populations. One cheater type was characterized by very low per-site heterozygosity and appeared haploid at all but the mating type locus (where heterozygosity was maintained), with relative sequence coverage supporting haploids having either a second copy of the entire chromosome III or just the mating type locus itself. The other cheater type appears to have fixed a single, heterozygous highly recombinant diploid clone (see Note S1). In both cases, it is possible the clone that came to dominate the population was able to both exploit our scheme for enforcing outcrossing, and perhaps had a mutation of large effect that allowed it to survive the chemical challenge. The remaining populations appear to have evolved in a manner typical of sexual outbreds, with considerable genome-wide heterozygosity and raw haplotype frequencies varying in frequency across the genome.

All evolved populations were thus classified into one of three classes: aneuploid haploid, clonal diploid, or outbred sexual. Per-site heterozygosity and genome-wide haplotype frequency profiles were used as metrics for classification. Specifically, populations with per-site heterozygosity profiles close to 0 with a single haplotype fixed genome-wide (except at the mating-type locus) were classified as aneuploid haploids. Populations with a bimodal per-site heterozygosity profile (with peaks centered close to 0 and 0.5) and with per-site haplotype frequencies either fixed for a single haplotype or split evenly between two haplotypes were classified as clonal diploid. The remaining populations were characterized by per-site heterozygosity profiles with a unimodal distribution and negative skew, while haplotype frequencies were highly heterogeneous throughout the genome and were classified as outbred sexual.

Determining recombination rate in budding yeast and D. melanogaster: Two closely related metrics for estimating the recombination rate in *S. cerevisiae* vs *D. melanogaster* are used. The first estimates the average recombination rate per generation per Mb. This is calculated as $90 * (1/18)/12 = 0.42$, due to an estimated 90 crossovers occurring in budding yeast during meiosis with a single meiosis every 18 mitotic generations and a genome size of approximately 12Mb. For *D. melanogaster*, the sex-averaged recombination rate is calculated as $5 * (1/2)/120 = 0.02$, due to an estimated 5 crossovers occurring per generation in females only and a genome size of approximately 120Mb. The per-gene recombination rate is estimated similarly, with size of the genome replaced by the number of genes- approximately 14,000 in *D. melanogaster* for a recombination rate of ~0.0002 per generation per gene as compared to approximately 6,275 genes in budding yeast for a recombination rate of ~0.0008 per generation per gene.

Determining sequence-level conservation at potentially functional variants: Sequence-level conservation information was taken from the UCSC genome browser based on a phylogenetic hidden Markov model (phastCons) comparing seven species of the genus *Saccharomyces*. Sequences were called as highly conserved if the level of conservation was at least 60%.

Detecting chromosomal or segmental duplications: To scan for the presence of large-scale duplications within the 55 evolved populations that passed all filters,

coverage was determined in 2kb non-overlapping intervals throughout the genome in the base and evolved populations. The relative coverage of each site was computed and the ratio of relative coverage of the evolved populations over the base population was calculated to determine the normalized fold-coverage at each site. Sites with normalized fold-coverage greater than or equal to 1.25 (which signifies that the relative coverage in the evolved population is at least 25% greater than in the base population at a particular site) were used as input for a Hidden Markov Model to predict the bounds of large, duplicated regions, which was carried out in R using a custom script.

Detecting de novo single nucleotide variants: To scan for the presence of *de novo* mutations in all evolved populations, SNPs found in our evolved populations were considered as potential candidate *de novo* SNVs if they passed a series of filters, including: excluding SNPs present in any of the original founders or base population, including clones directly derived from the base population (Linder et al. 2020), excluding sites with less than 10x sequencing coverage, and only including SNVs at which at least 20% of the reads were called as the mutant allele. Further, to filter out spurious mutations that may have resulted as artifacts of the culturing conditions in general, mutations that showed up in more than five different chemical treatments were excluded. SNPeff (Cingolani, Platts, et al. 2012) and SNPsift (Cingolani, Patel, et al. 2012) were used to predict the impact of all *de novo* mutations detected that occurred in coding regions of annotated genes. Intergenic SNVs were then further annotated based on genomic overlap with features other than genes using the saccharomyces_cerevisiae_R64-2-1_20150113.gff file downloaded from the SGD website. Intergenic SNVs that did not overlap any known annotated genomic features were further analyzed by using them as input for the R package motifbreakR (Coetzee et al. 2015), which predicts whether or not SNPs disrupt predicted transcription factor

binding sites. The list of *de novo* SNVs were finally filtered to only include the 55 evolved populations analyzed throughout this study.

Supplemental References

Ahyayauch H, Goñi FM, Bennouna M. 2003. pH-dependent effects of chlorpromazine on liposomes and erythrocyte membranes. *J. Liposome Res.* [Internet] 13:147–155. Available from: http://dx.doi.org/10.1081/lpr-120020317

Andreeva N, Ryazanova L, Dmitriev V, Kulakovskaya T, Kulaev I. 2013. Adaptation of Saccharomyces cerevisiae to toxic manganese concentration triggers changes in inorganic polyphosphates. *FEMS Yeast Res.* [Internet] 13:463–470. Available from: http://dx.doi.org/10.1111/1567-1364.12049

Boer VM, Tai SL, Vuralhan Z, Arifin Y, Walsh MC, Piper MDW, de Winde JH, Pronk JT, Daran J-M. 2007. Transcriptional responses of Saccharomyces cerevisiae to preferred and nonpreferred nitrogen sources in glucose-limited chemostat cultures. *FEMS Yeast Res.* [Internet] 7:604–620. Available from: http://dx.doi.org/10.1111/j.1567-1364.2007.00220.x

Causton HC, Ren B, Koh SS, Harbison CT, Kanin E, Jennings EG, Lee TI, True HL, Lander ES, Young RA. 2001. Remodeling of yeast genome expression in response to environmental changes. *Mol. Biol. Cell* [Internet] 12:323–337. Available from: http://dx.doi.org/10.1091/mbc.12.2.323

Chabrier-Roselló Y, Giesselman BR, De Jesús-Andino FJ, Foster TH, Mitra S, Haidaris CG. 2010. Inhibition of electron transport chain assembly and function promotes photodynamic killing of Candida. *J. Photochem. Photobiol. B* [Internet] 99:117–125. Available from: http://dx.doi.org/10.1016/j.jphotobiol.2010.03.005

Chakrabortee S, Byers JS, Jones S, Garcia DM, Bhullar B, Chang A, She R, Lee L, Fremin B, Lindquist S, et al. 2016. Intrinsically Disordered Proteins Drive Emergence and Inheritance of Biological Traits. *Cell* [Internet] 167:369–381.e12. Available from: http://dx.doi.org/10.1016/j.cell.2016.09.017

Desfougères Y, Gerasimaitė RU, Jessen HJ, Mayer A. 2016. Vtc5, a Novel Subunit of the Vacuolar Transporter Chaperone Complex, Regulates Polyphosphate Synthesis and Phosphate Homeostasis in Yeast. *J. Biol. Chem.* [Internet] 291:22262–22275. Available from: http://dx.doi.org/10.1074/jbc.M116.746784

Dickson RC, Lester RL. 1999. Metabolism and selected functions of sphingolipids in the yeast Saccharomyces cerevisiae. *Biochim. Biophys. Acta* [Internet] 1438:305–321. Available from: http://dx.doi.org/10.1016/s1388-1981(99)00068-2

Ehrenreich IM, Torabi N, Jia Y, Kent J, Martis S, Shapiro JA, Gresham D, Caudy AA, Kruglyak L. 2010. Dissection of genetically complex traits with extremely large pools of yeast segregants. *Nature* [Internet] 464:1039–1042. Available from: http://www.pubmedcentral.nih.gov/articlerender.fcgi?artid=PMC2862354

Gaber RF, Styles CA, Fink GR. 1988. TRK1 encodes a plasma membrane protein required for high-affinity potassium transport in Saccharomyces cerevisiae. *Mol. Cell. Biol.* [Internet] 8:2848–2859. Available from: http://dx.doi.org/10.1128/mcb.8.7.2848-2859.1988

Gasch AP, Spellman PT, Kao CM, Carmel-Harel O, Eisen MB, Storz G, Botstein D, Brown PO. 2000. Genomic expression programs in the response of yeast cells to environmental changes. *Mol. Biol. Cell* [Internet] 11:4241–4257. Available from:

https://www.ncbi.nlm.nih.gov/pubmed/11102521

Gopalbhai K, Jansen G, Beauregard G, Whiteway M, Dumas F, Wu C, Meloche S. 2003. Negative Regulation of MAPKK by Phosphorylation of a Conserved Serine Residue Equivalent to Ser212 of MEK1*. *J. Biol. Chem.* [Internet] 278:8118–8125. Available from: https://www.sciencedirect.com/science/article/pii/S002192582086428X

Griffith JL, Coleman LE, Raymond AS, Goodson SG, Pittard WS, Tsui C, Devine SE. 2003. Functional genomics reveals relationships between the retrovirus-like Ty1 element and its host Saccharomyces cerevisiae. *Genetics* [Internet] 164:867–879. Available from: https://www.ncbi.nlm.nih.gov/pubmed/12871900

Haro R, Banuelos MA, Quintero FJ, Rubio F, Rodriguez-Navarro A. 1993. Genetic basis of sodium exclusion and sodium tolerance in yeast. A model for plants. *Physiol. Plant.* [Internet] 89:868–874. Available from: http://doi.wiley.com/10.1111/j.1399-3054.1993.tb05298.x

Haro R, Garciadeblas B, Rodríguez-Navarro A. 1991. A novel P-type ATPase from yeast involved in sodium transport. *FEBS Lett.* [Internet] 291:189–191. Available from: http://dx.doi.org/10.1016/0014-5793(91)81280-l

Helsen J, Voordeckers K, Vanderwaeren L, Santermans T, Tsontaki M, Verstrepen KJ, Jelier R. 2020. Gene Loss Predictably Drives Evolutionary Adaptation. *Mol. Biol. Evol.* [Internet] 37:2989–3002. Available from: http://dx.doi.org/10.1093/molbev/msaa172

Hofman-Bang J. 1999. Nitrogen catabolite repression in Saccharomyces cerevisiae. *Mol. Biotechnol.* [Internet] 12:35–73. Available from: http://dx.doi.org/10.1385/MB:12:1:35

Hohmann S. 2002. Osmotic stress signaling and osmoadaptation in yeasts. *Microbiol. Mol. Biol. Rev.* [Internet] 66:300–372. Available from: http://dx.doi.org/10.1128/mmbr.66.2.300-372.2002

Keasling JD, Hupf GA. 1996. Genetic manipulation of polyphosphate metabolism affects cadmium tolerance in Escherichia coli. *Appl. Environ. Microbiol.* [Internet] 62:743–746. Available from: http://dx.doi.org/10.1128/aem.62.2.743-746.1996

Liu G, Bergenholm D, Nielsen J. 2016. Genome-Wide Mapping of Binding Sites Reveals Multiple Biological Functions of the Transcription Factor Cst6p in Saccharomyces cerevisiae. *MBio* [Internet] 7. Available from: http://dx.doi.org/10.1128/mBio.00559-16

Lue PF, Kaplan JG. 1969. The aspartate transcarbamylase and carbamoyl phosphate synthetase of yeast: a multi-functional enzyme complex. *Biochem. Biophys. Res. Commun.* [Internet] 34:426–433. Available from: http://dx.doi.org/10.1016/0006-291x(69)90399-4

Metodiev MV, Matheos D, Rose MD, Stone DE. 2002. Regulation of MAPK function by direct interaction with the mating-specific Galpha in yeast. *Science* [Internet] 296:1483–1486. Available from: http://dx.doi.org/10.1126/science.1070540

Mira NP, Palma M, Guerreiro JF, Sá-Correia I. 2010. Genome-wide identification of Saccharomyces cerevisiae genes required for tolerance to acetic acid. *Microb. Cell Fact.* [Internet] 9:79. Available from: http://dx.doi.org/10.1186/1475-2859-9-79

Miyajima I, Nakafuku M, Nakayama N, Brenner C, Miyajima A, Kaibuchi K, Arai K, Kaziro Y, Matsumoto K. 1987. GPA1, a haploid-specific essential gene, encodes a yeast homolog of mammalian G protein which may be involved in mating factor signal transduction. *Cell* [Internet] 50:1011–1019. Available from: http://dx.doi.org/10.1016/0092-8674(87)90167-x

Muhieddine D, Moughnié M, Abdel-Razzak Z. 2019. Short communication: Chlorpromazine causes a time-dependent decrease of lipids in Saccharomyces cerevisiae. *Interdiscip. Toxicol.* [Internet] 12:41–44. Available from: http://dx.doi.org/10.2478/intox-2019-0006

Navarre C, Goffeau A. 1998. The plasma membrane H(+)-ATPase of Saccharomyces cerevisiae is regulated by two small 38-residue isoproteolipids. *Folia Microbiol.* [Internet] 43:206–208. Available from: http://dx.doi.org/10.1007/BF02816516

Nehlin JO, Ronne H. 1990. Yeast MIG1 repressor is related to the mammalian early growth response and Wilms' tumour finger proteins. *EMBO J.* [Internet] 9:2891–2898. Available from: https://www.ncbi.nlm.nih.gov/pubmed/2167835

Nierras CR, Warner JR. 1999. Protein Kinase C Enables the Regulatory Circuit That Connects Membrane Synthesis to Ribosome Synthesis in Saccharomyces cerevisiae *. *J. Biol. Chem.* [Internet] 274:13235–13241. Available from: https://www.sciencedirect.com/science/article/pii/S0021925818368716

Roelofs J, Park S, Haas W, Tian G, McAllister FE, Huo Y, Lee B-H, Zhang F, Shi Y, Gygi SP, et al. 2009. Chaperone-mediated pathway of proteasome regulatory particle assembly. *Nature* [Internet] 459:861–865. Available from: http://dx.doi.org/10.1038/nature08063

Ruotolo R, Marchini G, Ottonello S. 2008. Membrane transporters and protein traffic networks differentially affecting metal tolerance: a genomic phenotyping study in yeast. *Genome Biol.* [Internet] 9:R67. Available from: http://dx.doi.org/10.1186/gb-2008-9-4-r67

Saeki Y, Toh-E A, Kudo T, Kawamura H, Tanaka K. 2009. Multiple proteasome-interacting proteins assist the assembly of the yeast 19S regulatory particle. *Cell* [Internet] 137:900–913. Available from: http://dx.doi.org/10.1016/j.cell.2009.05.005

Samanfar B, Omidi K, Hooshyar M, Laliberte B, Alamgir M, Seal AJ, Ahmed-Muhsin E, Viteri DF, Said K, Chalabian F, et al. 2013. Large-scale investigation of oxygen response mutants in Saccharomyces cerevisiae. *Mol. Biosyst.* [Internet] 9:1351–1359. Available from: http://dx.doi.org/10.1039/c3mb25516f

Shiraishi E, Inouhe M, Joho M, Tohoyama H. 2000. The cadmium-resistant gene, CAD2, which is a mutated putative copper-transporter gene (PCA1), controls the intracellular cadmium-level in the yeast S. cerevisiae. *Curr. Genet.* [Internet] 37:79–86. Available from: http://dx.doi.org/10.1007/s002940050013

Siegenthaler KD, Pareja KA, Wang J, Sevier CS. 2017. An unexpected role for the yeast nucleotide exchange factor Sil1 as a reductant acting on the molecular chaperone BiP. *Elife* [Internet] 6. Available from: http://dx.doi.org/10.7554/eLife.24141

Souciet J-L, Hubert J-C, Lacroute F. 1982. Cloning and restriction mapping of the yeastURA2 gene coding for the carbamyl phosphate synthetase aspartate-transcarbamylase complex. *Mol. Gen. Genet.* [Internet] 186:385–390. Available from: https://doi.org/10.1007/BF00729458

Takabatake A, Kawazoe N, Izawa S. 2015. Plasma membrane proteins Yro2 and Mrh1 are required for acetic acid tolerance in Saccharomyces cerevisiae. *Appl. Microbiol. Biotechnol.* [Internet] 99:2805–2814. Available from: http://dx.doi.org/10.1007/s00253-014-6278-2

Thorpe GW, Fong CS, Alic N, Higgins VJ, Dawes IW. 2004. Cells have distinct mechanisms to maintain protection against different reactive oxygen species: oxidative-stress-response genes. *Proc. Natl. Acad. Sci. U. S. A.* [Internet] 101:6564–6569. Available from: http://dx.doi.org/10.1073/pnas.0305888101

Thorsen M, Perrone GG, Kristiansson E, Traini M, Ye T, Dawes IW, Nerman O, Tamás MJ. 2009. Genetic basis of arsenite and cadmium tolerance in Saccharomyces cerevisiae. *BMC Genomics* [Internet] 10:105. Available from: http://dx.doi.org/10.1186/1471-2164-10-105

Trilisenko L, Kulakovskaya E, Kulakovskaya T. 2017. The cadmium tolerance in Saccharomyces cerevisiae depends on inorganic polyphosphate. *J. Basic Microbiol.* [Internet] 57:982–986. Available from: http://dx.doi.org/10.1002/jobm.201700257

Wang J, Pareja KA, Kaiser CA, Sevier CS. 2014. Redox signaling via the molecular chaperone BiP protects cells against endoplasmic reticulum-derived oxidative stress. *Elife* [Internet] 3:e03496. Available from: http://dx.doi.org/10.7554/eLife.03496

Wemmie JA, Wu AL, Harshman KD, Parker CS, Moye-Rowley WS. 1994. Transcriptional activation mediated by the yeast AP-1 protein is required for normal cadmium tolerance. *J. Biol. Chem.* [Internet] 269:14690–14697. Available from: https://www.ncbi.nlm.nih.gov/pubmed/818207
